# Lipoprotein(a) and Prostate Cancer: A Systematic Review of Observational, Genetic and Mendelian Randomization Evidence

**DOI:** 10.3390/ijms27188274

**Published:** 2026-09-17

**Authors:** Mikołaj Kisiała, Wojciech Bajurny, Jakub Karwacki, Aleksandra Marcinkowska, Mateusz Litarski, Michał Tulski, Dominik Zawadzki, Patryk Patrzałek, Adam Gurwin, Łukasz Nowak, Wojciech Krajewski, Tomasz Szydełko, Bartosz Małkiewicz

**Affiliations:** 1 Department of Minimally Invasive and Robotic Urology, University Center of Excellence in Urology, Wroclaw Medical University, 50-556 Wroclaw, Poland; 2 Department of Pathophysiology, Wroclaw Medical University, 50-368 Wroclaw, Poland

**Keywords:** prostate cancer, lipoprotein(a), biomarkers, Mendelian randomization, systematic review

## Abstract

Cancer is a major cause of morbidity worldwide. Prostate cancer (PCa) is one of the most commonly diagnosed malignancies in men and is clinically heterogeneous, highlighting the need for additional biomarkers that may improve risk stratification. Lipoprotein(a) [Lp(a)] is a genetically determined and increasingly available biomarker, but its relationship with PCa remains uncertain. This systematic review aimed to synthesize observational, genetic, and Mendelian randomization evidence on the associations of circulating Lp(a), LPA-related genetic variants, and genetically predicted Lp(a) with prostate cancer incidence, mortality, and clinicopathological features, and to assess the potential clinical implications of the available evidence. We conducted this review in accordance with PRISMA guidelines. PubMed, Scopus, and Web of Science databases were searched from inception to May 2026. Eligible studies were original, peer-reviewed studies in English that assessed circulating Lp(a), LPA-related genetic variants, or genetically predicted Lp(a) in relation to prostate cancer incidence, mortality, or clinicopathological features. Certainty of evidence was assessed using GRADE. Due to heterogeneity in study designs, exposure assessments, endpoints, and effect measures, results were synthesized narratively; no meta-analysis was performed. The search identified 489 records. After duplicate removal, 253 records were screened, 15 full-text reports were assessed, and 12 studies were included. These comprised prospective cohort or biobank-observational studies, observational studies, one genetic cohort study, and Mendelian randomization studies. Studies of circulating Lp(a) showed inconsistent findings for prostate cancer incidence and mortality. One cohort reported a positive association in a categorical analysis, whereas other studies found no association or a non-significant inverse association. The genetic cohort study found no robust association between LPAL2–LPA variants and prostate cancer incidence after correction for multiple testing. Mendelian randomization findings were mixed. Although several analyses yielded directionally positive or statistically significant estimates, several primary analyses were null or borderline, and estimates varied across methods and datasets. Overall, the Mendelian randomization evidence was of low certainty and does not establish a causal association between genetically predicted Lp(a) and PCa risk. Evidence for clinicopathological features was limited and inconsistent. Certainty of evidence was low for Mendelian randomization studies and very low for circulating Lp(a), genetic cohort evidence, and clinicopathological outcomes. Current evidence regarding Lp(a), LPA genetic variants, and genetically predicted Lp(a) in PCa remains heterogeneous and clinically inconclusive. Although some studies reported positive associations, the overall evidence linking Lp(a) to prostate cancer is inconsistent and of low certainty, and does not currently support a clear association.

## 1. Introduction

Cancer remains a major cause of morbidity and mortality worldwide, with prostate cancer (PCa) representing the second most commonly diagnosed malignancy in men globally [1]. PCa is a clinically heterogeneous disease, ranging from indolent tumors suitable for active surveillance to aggressive disease requiring radical or systemic treatment. Consequently, there remains a need to better understand the biological processes that contribute to clinically significant prostate cancer (csPCa), disease aggressiveness, and progression. Increasing attention has therefore been directed toward molecular and metabolic pathways that are not directly reflected by prostate-specific antigen (PSA), including alterations in lipid metabolism and related processes [2,3,4,5,6].

Dysregulated lipid metabolism is a relevant feature of PCa. Clinical lipidomic studies have identified tumor-associated changes in the lipidome of PCa cells, while experimental studies suggest that low-density lipoprotein (LDL)-cholesterol increases PCa cell viability, proliferation, and migration [7,8]. Increased fatty acid uptake has also been demonstrated in PCa, and its pharmacological suppression showed therapeutic effects in experimental models [9]. Cholesteryl ester accumulation has also been linked to PTEN loss, PI3K/AKT activation, and aggressive PCa features [10]. Lipoprotein(a) [Lp(a)] is an established cardiovascular biomarker that has attracted increasing interest in oncology [11]. Emerging evidence suggests that Lp(a) may be associated with several cancer types, including pancreatic cancer [12], lung cancer [13], and PCa [14]. Lp(a) is an LDL-like particle and may exert opposing effects on tumor biology: its oxidized phospholipid cargo and apo(a)-related growth-factor-like properties provide plausible pro-inflammatory and pro-proliferative mechanisms that could favor carcinogenesis, although a direct carcinogenic effect of intact Lp(a) has not been demonstrated [15,16,17,18,19,20]. Conversely, apo(a) and its kringle-containing fragments have shown anti-angiogenic activity, including inhibition of endothelial-cell migration and proliferation through focal adhesion kinase (FAK) and c-Src/ERK signaling, while apo(a)-related effects on fibrinolysis may inhibit plasmin-dependent extracellular-matrix remodeling and cell motility [21,22,23,24,25,26]. Because much of this evidence derives from non-prostatic systems, experimental tumor models, or isolated apolipoprotein(a) fragments, the net effect of Lp(a) on PCa initiation and progression remains uncertain.

Lp(a) concentrations are largely genetically determined and relatively stable throughout life [27]. Because Lp(a) measurement is recommended at least once in adulthood for cardiovascular risk assessment and is increasingly being incorporated into preventive care pathways, including national health-check programs in some countries, its widespread clinical measurability makes Lp(a) an accessible candidate exposure for investigating potential associations with diseases beyond cardiovascular medicine; however, its clinical utility as a PCa biomarker has not been established [28,29]. The potential role of Lp(a) in prostate carcinogenesis remains unresolved. Several studies have suggested that higher circulating or genetically predicted Lp(a) may be associated with increased PCa risk and with adverse clinicopathological features, raising the possibility that Lp(a) could contribute to PCa development [14,30,31]. Together with the established alterations in lipid and cholesterol metabolism in prostate cancer, these observations provide a biological rationale for investigating the relationship between Lp(a) and PCa.

To our knowledge, no prior systematic review has synthesized the evidence on Lp(a) and PCa. The available literature is limited and heterogeneous, and it remains unclear whether reported associations represent a reproducible biological relationship, a causal effect, or findings of potential clinical relevance. We therefore systematically reviewed circulating Lp(a), LPA-related genetic, and Mendelian randomization (MR) evidence in relation to PCa incidence, mortality, and clinicopathological characteristics. Particular attention was given to the consistency of findings across study designs, evidence concerning aggressive disease phenotypes, the strength of causal inference, and the extent to which the available data support—or do not support—further evaluation of Lp(a) as a potential PCa biomarker.

## 2. Methods

### 2.1. Search Strategy

We prepared this systematic review in accordance with the PRISMA guidelines. The protocol for this study was prepared and published in the PROSPERO database of systematic review protocols (CRD420261326782). A literature search covering the period from database inception to 20 May 2026 was conducted, with the last search performed on 20 May 2026. We searched the following scientific databases: PubMed (Medline), Scopus (Elsevier), Web of Science (Clarivate). No database filters were applied. During screening, we narrowed down the inclusion criteria to peer-reviewed and English-only studies. Searches were applied to title/abstract/keyword and controlled vocabulary with appropriate modifications for each database. The PubMed search query was: (“Lipoprotein(a)”[mh] OR “Lipoprotein(a)”[tiab] OR “lipoprotein a”[tiab] OR “lipoprotein(a)”[tiab] OR “Lp(a)”[tiab] OR “LPA”[tiab]) AND (“Prostatic Neoplasms”[Mesh] OR “prostate”[tiab] OR “prostate cancer”[tiab] OR “prostatic neoplasm*”[tiab] OR “prostate neoplasm*”[tiab] OR “prostate carcinoma”[tiab]). Full search queries for each database are available in the Supplementary Material S1.

### 2.2. Eligibility Criteria

The review question and eligibility criteria were structured according to the PECO framework (Population-Exposure-Comparator-Outcome) [32]. The PECO framework was used to define the eligibility criteria across the different evidence streams included in this review, encompassing observational studies of circulating Lp(a), studies of LPA-related genetic variants, and MR studies of genetically predicted Lp(a). Accordingly, the exposure and comparator definitions were adapted to the specific study design while maintaining PCa-related outcomes as the common outcome domain.

PECO:

Population: Adult men diagnosed with PCa or at risk of PCa.

Exposure: Circulating Lp(a) concentration, LPA-related genetic variants, or genetically predicted Lp(a).

Comparator: Men with lower or reference Lp(a) concentrations, men without PCa, or men carrying reference genetic variants associated with lower or typical Lp(a) levels.

Outcome: Any measure assessing the association of LPA-related genetic variants or circulating Lp(a) concentrations with PCa incidence, mortality, or clinicopathological features.

We included papers that investigated Lp(a) and its effect on PCa incidence or clinicopathological features. We excluded studies if they did not assess Lp(a), LPA variants, or genetically predicted Lp(a), did not report PCa outcomes, or assessed only other lipid biomarkers, such as low-density lipoprotein cholesterol (LDL-C), high-density lipoprotein cholesterol (HDL-C), triglycerides, total cholesterol, apolipoprotein A, apolipoprotein B, proprotein convertase subtilisin/kexin type 9 (PCSK9), or angiopoietin-like protein 3 (ANGPTL3), without data on Lp(a). We excluded reviews, meta-analyses, editorials, letters, comments, case reports, animal studies, in vitro studies, grey literature, and conference abstracts. All retrieved records were imported into Rayyan and duplicates were removed using dedicated duplicate removal function in the app [33]. Three reviewers (M.K., W.B., J.K.) independently screened titles and abstracts against the eligibility criteria. Disagreements were resolved through discussion; if consensus could not be reached, the senior author (B.M.) was consulted. Articles eligible for full-text review were screened by three independent reviewers, and conflicts were resolved as above. The initial search yielded 489 records; after removing duplicates, 253 remained. After initial screening, 15 records were eligible for full-text screening, and 12 reports were deemed eligible and were included in the review.

### 2.3. Data Items

Data extraction was performed by two independent reviewers (M.K. and W.B.) and discrepancies were resolved by discussion or consultation with the senior author (B.M.). We extracted the following data: author and year, country of origin, study design, data source, study period, population, sample size, participant characteristics, exposure type, Lp(a) measurement, exposure coding, genetic instrument details, outcomes assessed, outcome ascertainment, time points, effect estimate for incidence and mortality, effect estimate for clinicopathological features, definition of aggressive or csPCa, adjusted covariates (observational studies), MR assumptions/sensitivity, and key results (Supplementary Material S9).

### 2.4. Synthesis Methods

The feasibility of quantitative synthesis was assessed within design- and outcome-specific groups. Although a highest-versus-lowest category estimate could be theoretically calculated for some studies assessing Lp(a) concentrations and PCa incidence, this would combine quartiles with quintiles defined by substantially different Lp(a) ranges and assays; moreover, mg/L and nmol/L cannot be reliably converted using a fixed factor, and the available results did not support a common linear dose–response model. Pooling was not feasible for PCa-specific mortality, conventional genetic associations, or clinicopathological outcomes because these groups contained only one directly comparable estimate or used incompatible populations, outcome definitions, and effect measures. MR estimates were also unsuitable for pooling because several studies reused UK Biobank exposure data and overlapping PRACTICAL or FinnGen outcome datasets, violating statistical independence and potentially producing spuriously narrow confidence intervals. Thus, results were synthesized narratively and presented in tables. The synthesis was first structured by outcome domain (PCa incidence and mortality versus clinicopathological features) and, within each outcome domain, by evidence type or study design (circulating Lp(a) measurements, genetic association studies, and MR studies). Within each group, findings were compared according to the direction and magnitude of reported effect estimates, statistical precision, and consistency across studies or analytical methods. Where findings differed, they were presented comparatively and interpreted in relation to differences in study design, exposure and outcome definitions, analytical approaches, and methodological limitations. Effect estimates were extracted as reported. No formal statistical heterogeneity or sensitivity analyses were performed because quantitative pooling was not appropriate.

### 2.5. Risk of Bias and Certainty Assessment

Risk of bias (RoB) was assessed using design-specific tools: ROBINS-E for observational exposure studies (Supplementary Material S2–S5, QUIPS for prognostic factor studies assessing clinicopathological outcomes (Supplementary Material S6), Q-Genie for the genetic cohort study (Supplementary Material S7), and a RoB tool adapted from Mamluk et al. [34] for MR studies, with modifications tailored to the objectives of this review (see Supplementary Material S8 and S10). RoB assessments were performed independently by two reviewers (W.B., J.K.). Disagreements were resolved through discussion or, when necessary, consultation with a third reviewer (M.K.). RoB data were reported in tables and in Supplementary Material. The assessment considered the availability of protocols or analysis plans, consistency between methods and reported outcomes, completeness of PCa-related results, reporting of significant and non-significant findings, and availability of sufficient extractable data. The assessment was guided by a structured checklist adapted from the reporting-bias domains of risk-of-bias tools. No new formal tool was developed. Disagreements were resolved by discussion or consultation with a third reviewer. Study investigators were not contacted for additional unpublished data.

Certainty of evidence was assessed using the GRADE approach [35], separately for each main outcome and synthesis group. The assessed domains included risk of bias, inconsistency, indirectness, imprecision, and publication or reporting bias. Observational evidence was initially rated with low certainty and downgraded due to serious concerns. For MR studies, additional considerations included instrument strength, pleiotropy, population stratification, sample overlap, and consistency of sensitivity analyses. Overall certainty was classified as high, moderate, low, or very low according to standard GRADE criteria. No review-specific minimal important difference threshold was defined due to heterogeneity in study designs, exposure coding, outcomes, and effect measures. Imprecision was judged based on confidence interval width and whether intervals crossed the null or included both potentially increased and decreased risk. Two reviewers independently assessed certainty, with disagreements resolved by discussion or consultation with a third reviewer. Study investigators were not contacted, and no automation tool was used to assign final GRADE judgments. Certainty ratings are reported in structured tables and Supplementary Material S11.

## 3. Results

### 3.1. Study Characteristics

**Figure 1:**
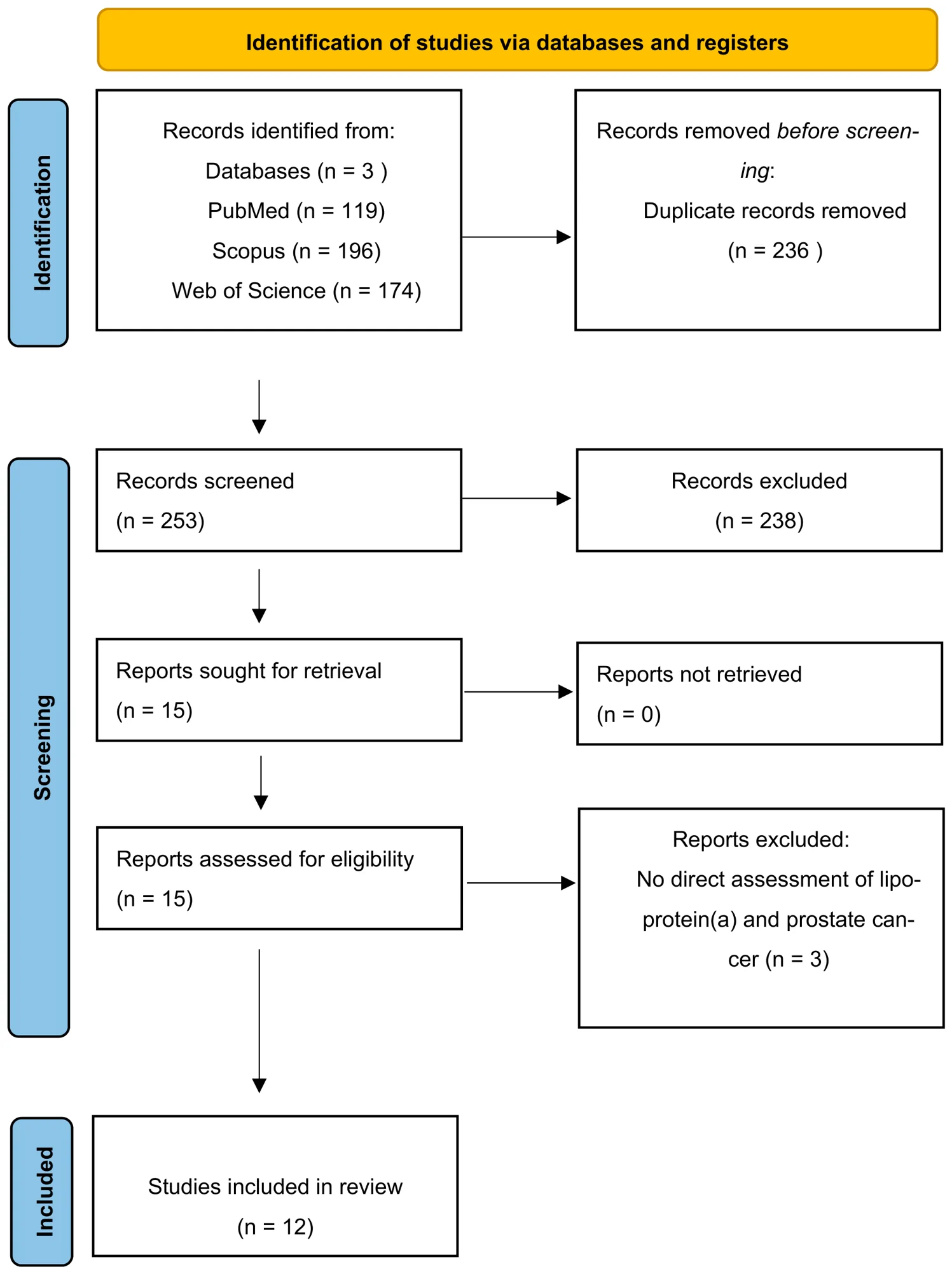
PRISMA flow diagram illustrating the identification, screening, eligibility assessment, and inclusion of studies in the systematic review.

**Figure 2:**
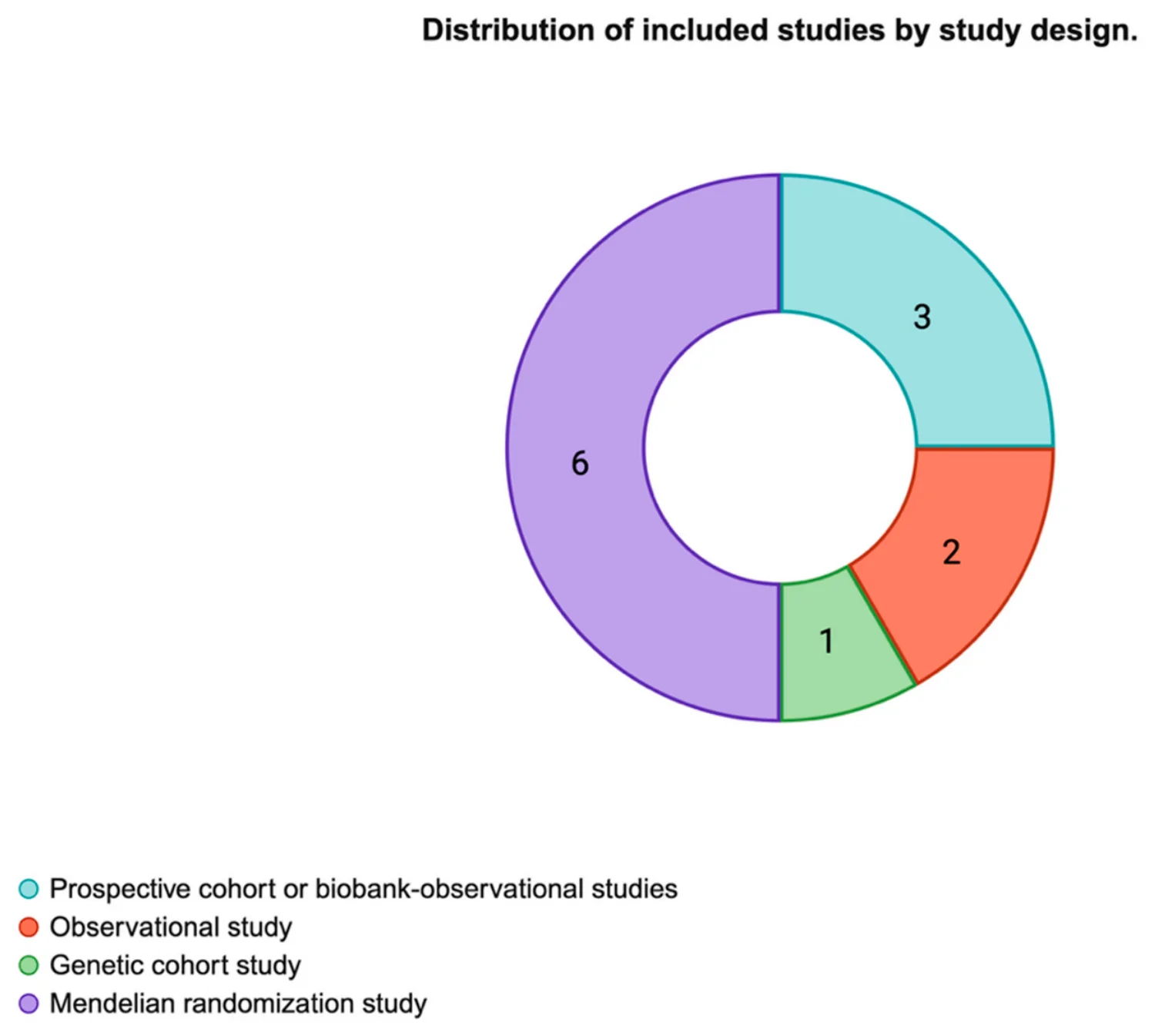
Distribution of included studies by study design. Created in BioRender. Kisiała, M. (2026) https://BioRender.com/j8n0c4w (accessed on 20 July 2026).

The electronic database search identified 489 records. Potential duplicate records were identified using Rayyan’s duplicate-detection function, verified by the reviewers, and removed before screening. After removal of 236 duplicate records, 253 unique records remained for title and abstract screening. After title and abstract screening, 15 records were selected and sought for retrieval; 15 studies were accessible and underwent full-text screening (Figure 1). Twelve studies (Supplementary Material S2) met the inclusion criteria and are presented in the review. Included studies measured circulating Lp(a), LPA genetic variants, and genetically proxied Lp(a) in relation to PCa. The included evidence originated from several study settings. Observational studies on serum concentrations of Lp(a) and PCa were conducted in Germany [36], France [30], the United Kingdom and Sweden [37], China [14], and Canada [38]. One genetic cohort study was conducted in Japan [39]. MR studies were mainly based on large, publicly available, or consortium-level genetic datasets, including the UK Biobank, PRACTICAL, FinnGen, the Global Lipids Genetics Consortium, and IEU Open GWAS resources. These studies were conducted in China [40,41,42,43], the UK and Denmark [44] UK, USA, Norway, and Greece [31]. The methodology of included studies comprised prospective cohort or biobank-observational studies [30,36,37], observational studies with measured circulating Lp(a) [14,38], a genetic cohort study [39], and MR studies [31,40,41,42,43,44] (Figure 2). PCa incidence and mortality were evaluated in 10 studies [30,31,36,37,39,40,41,42,43,44], and clinicopathological adverse features were evaluated in 3 studies [14,31,38].

### 3.2. Incidence and Mortality of Prostate Cancer

#### 3.2.1. Serum-Based Lp(a) Measurements

Three studies assessed circulating Lp(a) concentrations in relation to PCa incidence or mortality (Table 1; Supplementary Table S1). Collectively, the findings were inconsistent and did not demonstrate a clear association between circulating Lp(a) and PCa outcomes. Katzke et al. reported a higher PCa incidence when comparing the highest with the lowest Lp(a) quartile (adjusted HR 1.43, 95% CI 1.02–2.03); however, the quartile-median trend was not statistically significant (*p* = 0.085), and the continuous analysis per doubling of Lp(a) was borderline (HR 1.06, 95% CI 1.00–1.13; *p* = 0.051) [36]. Perez-Cornago et al. found no clear association with PCa incidence (HR 1.02, 95% CI 0.99–1.05) and no significant association with PCa mortality; the continuous mortality model yielded an HR of 1.03 (95% CI 0.91–1.16) per 1-standard deviation (SD) higher Lp(a) [37]. Marrer et al. reported a non-significant, directionally inverse association for the highest versus lowest Lp(a) quartile (HR 0.84, 95% CI 0.49–1.45), which remained similar after excluding cancers diagnosed during the first two years of follow-up (HR 0.87, 95% CI 0.50–1.51) [30]. The observational evidence for circulating Lp(a) was inconsistent and rated as very low certainty.

#### 3.2.2. Mendelian Randomization Studies

Six MR studies evaluated genetically predicted Lp(a) in relation to PCa incidence and mortality. Findings were mixed across studies and analytical approaches. The direction and statistical significance of the estimates varied by method, genetic instrument, and dataset (Table 2; Supplementary Table S2). Some effect estimates suggested a modest increase in PCa risk per genetically predicted increase in Lp(a) [40,41,42,43]. However, the primary inverse-variance weighted (IVW) analyses in Ioannidou et al. and Fang et al. were null, with ORs of 1.066 (95% CI 0.909–1.249; *p* = 0.431) and 1.06 (95% CI 0.95–1.20; *p* = 0.305), respectively [31,44]. Several studies reported statistically significant associations using weighted-median, multivariable MR, LPA-restricted instruments, or validation datasets [31,40,42,43,44]. Positive primary estimates were reported by Chen et al. (OR 1.061, 95% CI 1.018–1.105; *p* = 0.005), Lin et al. (OR approximately 1.12; *p* = 0.038), and Li et al. (OR 1.12, 95% CI 1.01–1.25; *p* = 0.038). One study also reported colocalization evidence, which also supported the association between higher genetically predicted Lp(a) and PCa risk [41]. However, the interpretation of these results must be carried out with caution, as it is limited by several factors including potential pleiotropy, instrument selection, methodological heterogeneity, and overlap between datasets. In summary, the MR evidence provides limited and low-certainty evidence of a possible positive relationship between genetically predicted Lp(a) and PCa risk.

#### 3.2.3. Genetic Studies

One prospective genetic cohort study assessed variants in the LPAL2–LPA region in relation to PCa incidence. Although several suggestive associations were observed, none remained statistically significant after correction for multiple testing. Overall, this study provided no robust evidence of an association between LPA-region genetic variation and PCa incidence [39].

### 3.3. Clinicopathological Features

#### 3.3.1. Serum-Based Lp(a) Measurements

Two observational studies assessed circulating Lp(a) in relation to clinicopathological features of PCa with inconsistent findings. One study reported associations between higher Lp(a) concentrations and several adverse disease characteristics, including high-risk or more advanced disease, whereas the other found no significant difference in Lp(a) concentrations between men with localized high-grade PCa and at-risk controls [14,38]. The evidence for an association between circulating Lp(a) and adverse clinicopathological features was limited, inconsistent, and rated as very low certainty.

#### 3.3.2. Mendelian Randomization Studies

One MR study assessed genetically predicted Lp(a) in relation to advanced PCa (metastatic disease, Gleason score ≥8, PSA > 100 ng/mL, or PCa death) [31]. The main univariable estimates were not statistically significant, but analyses using LPA-restricted instruments showed a stronger positive association (weighted-median OR, 1.388; 95% CI, 1.213–1.590; *p* = 2.14 × 10^−6^). In multivariable MR, the association was borderline and remained directionally positive. Although analyses using LPA-restricted instruments suggested a positive association, the main univariable estimates were not statistically significant, and the multivariable estimate was borderline significant. Overall, the evidence for an association between genetically predicted Lp(a) and advanced PCa was limited and of very low certainty.

#### 3.3.3. Risk of Bias and Certainty

Risk of bias varied across study designs (Table 3). Among observational studies of circulating Lp(a), Bergeron et al. [38] was judged to have a high overall risk of bias, whereas Katzke et al. [36], Marrer et al. [30], and Perez-Cornago et al. [37] were rated as having some concerns, mainly related to confounding, exposure measurement, participant selection, and selective reporting (Supplementary Materials S2–S5). The clinicopathological study by Wang et al. [14] had a moderate overall risk of bias, with particular concern regarding statistical analysis and reporting (Supplementary Material S6). The genetic cohort study by Mieno et al. [39] was classified as good quality using Q-Genie (Supplementary Material S7). Among MR studies, Fang et al. [44], An et al. [41], and Li et al. [42] were rated as having low overall risk of bias, while Ioannidou et al. [31], Chen et al. [40], and Lin et al. [43] were judged to have a moderate risk because of concerns involving instrument validity, pleiotropy, population structure, or selection bias (Supplementary Material S8).

The certainty of evidence was low or very low for all outcomes. MR evidence for PCa incidence and mortality was rated as low certainty, whereas evidence from circulating Lp(a) studies and the genetic cohort study was rated as very low certainty. Evidence concerning clinicopathological features was also rated as very low certainty for both circulating and genetically predicted Lp(a). These judgments reflected the small number of studies, heterogeneity in design and exposure assessment, inconsistent findings, imprecision, and methodological limitations. No outcome was supported by moderate- or high-certainty evidence (Table 4).

## 4. Discussion

To our knowledge, this is the first systematic review to evaluate the relationship between Lp(a) and PCa. Overall, the available evidence does not demonstrate a consistent relationship between Lp(a) and PCa incidence, mortality, or adverse clinicopathological features. For circulating Lp(a), the only significant association with PCa incidence was observed by Katzke et al. [36] when the highest and lowest quartiles were compared. However, the trend across quartiles and the continuous analysis were non-significant or borderline. The prospective case–cohort design of this study and prediagnostic blood samples established the temporal sequence between exposure and outcome, and the validity was supported by registry ascertainment. However, the lack of a clear dose–response pattern weakens the evidence. The study also examined several biomarker–outcome associations without adjustment for multiple testing, so the isolated quartile finding may have occurred by chance.

Marrer et al. [30] found no association between Lp(a) and PCa incidence in the PRIME study. The highest Lp(a) category showed a non-significant tendency towards lower risk. The study design included prediagnostic fasting samples, annual follow-up, and histological confirmation of cases. Interpretation was limited by the small number of PCa cases and the restriction to middle-aged French men. In addition, the assay may have been affected by the size of apolipoprotein A isoforms. The largest and most informative analysis was conducted by Perez-Cornago et al. [37] on the UK Biobank database. This study examined both PCa incidence and PCa-specific mortality, used repeat Lp(a) measurements to reduce regression-dilution bias, and accounted for multiple testing. Lp(a) was not associated with either outcome. These results provide the strongest evidence against a clinically important association with overall PCa incidence. Nevertheless, the non-representative composition of the UK Biobank and potential selection bias may limit generalisability, while PSA testing patterns may have introduced detection bias. The relatively short follow-up and small number of PCa deaths also limited the assessment of PCa mortality.

In conclusion, the EPIC–Heidelberg finding was not supported by a dose–response relationship or replicated in PRIME or UK Biobank. Therefore, the available evidence does not indicate a clinically important association between circulating Lp(a) and overall PCa incidence. A weak or non-linear association cannot be excluded. Evidence on PCa-specific mortality remains limited because only UK Biobank examined this outcome directly, with relatively few deaths; thus, no conclusions can be drawn.

Beyond incidence and mortality, two studies examined circulating Lp(a) in relation to clinicopathological severity, although their designs and comparisons differed substantially. Wang and Zhang [14] assessed pathological features among patients with PCa, whereas Bergeron et al. [38] compared men with localized high-grade PCa with an at-risk control group. These two studies, however, differed in design and endpoints. The study by Wang and Zhang [14], which identified an association, provided a more robust analysis of clinicopathological features, including International Society of Urological Pathology (ISUP) grade, lymph-node involvement, and distant metastases, and had a larger study population. The study also adjusted its results for coronary artery disease and lipid-lowering drugs to account for cardiovascular diseases, which are correlated with advanced PCa at diagnosis [45]. However, the study was based on a cohort from a single institution, was subject to potential selection bias due to the inclusion of patients with complete records, and was retrospective in design. Causality also cannot be inferred due to the cross-sectional design. On the other hand, the study by Bergeron et al., which did not report an association between Lp(a) concentration and PCa, included a PCa-free control group and individual matching but had only 35 samples from patients with PCa and 35 controls. Despite the a priori power calculation, the paper did not state the assumed effect size or variability. Moreover, the SDs were large—108 nmol/L in the PCa group and 70 nmol/L in the control group—precluding detection of any effect smaller than a large one. Although the control group was considered “PCa-free,” it was at risk of PCa because of elevated PSA and/or a family history of PCa, which may itself have affected Lp(a) levels and diluted the association. Most importantly, the study did not adjust for ethnicity, smoking, diet, cardiovascular disease, diabetes, or inflammation.

Thus, given the methodological limitations of the available studies and the scarce body of evidence, neither the presence nor the absence of a correlation between Lp(a) concentrations and adverse clinicopathological features of PCa can be supported.

Only one available study directly examined associations between genetic variation in the LPAL2–LPA region and PCa incidence [39]. Four variants were nominally associated with incidence, but none remained significant after correction for multiple testing. These findings may therefore represent chance associations and require independent replication. Interpretation is further limited by the small number of PCa cases and the absence of a PCa-mortality analysis. The study examined only common variants identified in a Japanese population and did not capture KIV-2 copy-number variation, which is a major genetic determinant of Lp(a). Moreover, circulating Lp(a) was not measured, and the selected variants were not validated as instruments for Lp(a) in this cohort. The observed genetic associations therefore cannot be attributed specifically to changes in Lp(a). Broader coverage of ancestry-specific LPA variants would provide a more complete assessment of the locus. Variants from other genomic regions could increase power, but only if their associations with Lp(a) were independently validated and potential pleiotropy was carefully examined. The evidence is insufficient to support LPA genotyping for assessing PCa risk.

MR studies addressed the related yet distinct question of whether lifelong genetically predicted Lp(a) levels influence PCa risk. The MR evidence suggests a possible increase in PCa risk with genetically predicted Lp(a), but the association was not consistently supported by the most robust analyses. Ioannidou et al. [31] provided the most comprehensive Lp(a)-focused assessment by using large male-specific datasets, multivariable adjustment for correlated lipids, and several pleiotropy-sensitive methods; nevertheless, the primary analysis was null, and significance was reached after changing the estimator, instrument set, or adjustment model, which suggested that the result was dependent on the chosen method. Similarly, Fang et al. [44] used strong methodological triangulation for PCSK9, including gene-expression and colocalization analyses, but direct Lp(a) analysis was secondary and positive only in selected sensitivity and multivariable models. Thus, these convincing findings should not be interpreted as equally strong evidence for a direct effect of Lp(a) on PCa risk. Chen et al. and Li et al. [40,42] identified positive associations in broad biomarker screens and applied several sensitivity analyses, but their findings on Lp(a) and PCa risk did not survive correction for multiple testing; Chen et al. also did not use multivariable MR to separate Lp(a) from correlated lipid pathways. An et al. [41] provided the strongest specific support for an Lp(a)–PCa relationship through discovery and validation analyses, Bayesian methods, reverse-direction testing, meta-analysis, and LPA–PCa colocalization; however, the Bayesian estimate in the discovery cohort was null, and its datasets overlapped with those used in other papers. The apparent positive pattern may partly reflect the reuse of overlapping datasets and the sensitivity of the estimates to analytical choices. Therefore, MR evidence on Lp(a) and PCa risk does not establish a causal relationship, although a small positive effect cannot be ruled out.

Most MR studies focused on PCa incidence, while evidence concerning adverse clinicopathological features was limited to a single study [31]. The primary analyses for advanced disease were null. The multivariable estimate was marginally significant, and the isolated positive sensitivity analyses varied by analysis. The study also used cancer-free patients as controls rather than patients with non-advanced PCa; it also did not include locally advanced PCa characteristics such as extraprostatic extension (EPE) or seminal vesicle invasion (SVI) as endpoints, and used few Lp(a) genetic instruments, which limited precision. No colocalization analysis was performed, so the study could not confirm that the same LPA variant influenced both Lp(a) levels and adverse PCa features. We conclude that, based on the current MR evidence, which consists of only one study, it is not possible to draw a firm conclusion regarding the relationship between genetically predicted Lp(a) and adverse PCa features.

Viewed together, the evidence on PCa incidence is more consistent than the isolated positive findings suggest. Circulating studies assessed Lp(a) measured during adulthood, the conventional genetic study examined selected LPAL2–LPA variants, and MR estimated lifelong genetically predicted exposure. Despite these differences, the most reliable analyses were predominantly null, providing little support for a moderate linear effect of Lp(a) on PCa incidence. The apparent disagreement concerning clinicopathological features is more difficult to interpret because the circulating studies assessed disease severity among patients with established PCa, whereas MR compared advanced cases with cancer-free controls. The latter design combined susceptibility to PCa with the probability of advanced disease and did not directly evaluate progression among cases. Consequently, the circulating and MR findings are not directly contradictory, but neither establishes that Lp(a) causally influences PCa progression. Current evidence therefore does not support using circulating Lp(a) or LPA genotyping for PCa screening or risk stratification.

The biological effects of Lp(a) in PCa may be complex and potentially opposing, reflecting the distinct biological properties of its LDL-like component, apolipoprotein A, and oxidized phospholipids (OxPLs). Lp(a)-associated OxPLs exert well-established pro-inflammatory effects, providing a plausible mechanism through which elevated Lp(a) could contribute to a tumor-supportive inflammatory microenvironment [15,16,17]. However, this mechanism remains indirect in PCa: although inflammatory activity of Lp(a)-associated OxPLs is well documented, there is currently no direct evidence that physiological concentrations of intact Lp(a) induce pro-tumorigenic inflammatory signaling within prostate tissue. Similarly, because altered cholesterol metabolism contributes to PCa biology, the LDL-like component of Lp(a) could theoretically interact with lipid-dependent tumor pathways, but a specific role for Lp(a) as a cholesterol source for PCa cells remains speculative [11].

In contrast, the anti-angiogenic properties of apolipoprotein A and its kringle-containing fragments are better supported by experimental evidence. Apolipoprotein A fragments inhibit endothelial tube formation, migration, and proliferation [21], with proposed molecular mechanisms involving suppression of FAK signaling and inhibition of c-Src/ERK activation [22,23,24]. Apolipoprotein A-related interference with plasmin-dependent extracellular-matrix remodeling and cell motility represents another potentially relevant pathway [25,26]. Nevertheless, most of these observations derive from endothelial models, non-prostatic tumor models, or isolated apolipoprotein A fragments and not intact circulating Lp(a); therefore, they cannot be directly extrapolated to PCa. Direct carcinogenic effects of apolipoprotein A have not yet been demonstrated, and much of the mechanistic relationship between Lp(a) and cancer remains unresolved.

This review was limited by the small number of eligible studies and substantial heterogeneity in study designs, populations, exposure definitions, outcome assessments, covariate adjustments, and statistical methods, which precluded meta-analysis. Furthermore, restricting inclusion to peer-reviewed English-language articles may have led to the omission of relevant evidence. Some studies may also have been missed despite searching three major databases.

Evidence on clinicopathological outcomes was derived from a few small or single-center studies. Some MR analyses yielded directionally positive estimates; however, results were inconsistent between primary and sensitivity analyses and may have been influenced by pleiotropy, instrument selection, population structure, and dataset overlap. The predominantly European ancestry of the study populations also limits generalizability to populations with different Lp(a) concentrations and genetic backgrounds.

Future studies should involve large, prospective cohorts with standardized measurements of Lp(a) and clearly defined relevant endpoints, such as EPE, SVI, lymph-node involvement, distant metastasis, biochemical recurrence, and mortality specific to PCa. Special attention should be paid to longitudinal outcomes, as these may be more effective in differentiating between disease incidence and progression and in assessing prognosis. Further MR studies should make use of well-powered, independent datasets with enough power to identify the relatively small effect sizes suggested by the existing literature. The power calculations should take into account the proportion of variance in Lp(a) that is explained by the genetic instruments, and steps should be taken to minimize the overlap between the exposure and outcome datasets. Replication in independent datasets would also help to determine whether the positive findings seen in some secondary or restricted-instrument analyses can be reproduced. Lastly, since most of the available genetic evidence has come from populations of European ancestry, future observational and genetic studies should include additional diverse populations, especially those of non-European ancestry, in order to evaluate the consistency and generalizability of these associations across different genetic backgrounds and Lp(a) distributions. Moreover, mechanistic studies should distinguish the effects of intact Lp(a) from those of its individual components. Comparative experiments using intact Lp(a), apolipoprotein A, kringle fragments, OxPL-modified or OxPL-depleted Lp(a), and LDL in PCa cell lines, patient-derived organoids, and tumor–endothelial or tumor–immune co-culture models could assess inflammatory signaling, proliferation, lipid uptake, angiogenesis, extracellular matrix invasion, and cell migration. Such studies would help determine whether the epidemiological associations observed for Lp(a) reflect a direct effect of the intact particle, biological activity of specific apo(a)/OxPL components, or indirect systemic processes. This distinction is particularly important because existing preclinical evidence more consistently supports anti-angiogenic effects of apolipoprotein A fragments than a direct pro-carcinogenic action of intact Lp(a). The limited evidence suggests that Lp(a), if associated with PCa, may be more closely linked to aggressive or progressive disease than to overall incidence. However, the small number of studies and inconsistent definitions of advanced disease prevent firm conclusions.

In conclusion, the current evidence on the relationship between Lp(a) and PCa remains limited and inconsistent, and is insufficient to establish a clear role for Lp(a) in PCa development, progression, or related outcomes.

## Figures and Tables

**Table 1:** Summary of observational evidence on circulating Lp(a) and prostate cancer outcomes.

Study	Population	Factors Analyzed	Lp(a) Measurement	Adjustment Variables	Key Estimate	Overall Finding
Katzke et al. [36]	**Heidelberg cohort.** Case–cohort within 25,546 EPIC–Heidelberg participants: random subcohort n = 2739, including 1273 men, and 554 incident PCa cases. The exact Lp(a)-specific analytical number was not reported	PCa incidence	Serum based Lp(a) measurements	Age; height; waist circumference; BMI; lifetime alcohol consumption; red-meat and fibre intake; detailed smoking history; educational level; physical activity; diabetes; hypertension; and lipid-lowering medication	Q4 vs. Q1: adjusted HR 1.43 (95% CI 1.02–2.03)	Higher risk in the highest Lp(a) quartile, but the trend and continuous analyses were not statistically significant.
Perez-Cornago et al. [37]	**UK-Biobank**. Overall cohort: 211,754 men. The Lp(a) analyses included 4440 incident PCa cases and 240 PCa deaths among 163,568 men with available Lp(a) data	PCa incidence and mortality	Serum based Lp(a) measurements	Stratified by age and recruitment region; adjusted for Townsend deprivation score, ethnicity, height, living with a partner, BMI, smoking, physical activity, alcohol consumption, and diabetes	Incidence: HR 1.02 (95% CI 0.99–1.05)	No clear association with PCa incidence or PCa mortality.
Marrer et al. [30]	**PRIME-study cohort**. 5196 men, including 126 incident PCa cases	PCa incidence	Serum based Lp(a) measurements	PCa model was adjusted for centre, age, smoking, and alcohol consumption. Additional analyses considered education, employment status, BMI, systolic blood pressure, physical activity, history of dyslipidaemia, and lipid-lowering medication	Q4 vs. Q1: HR 0.84 (95% CI 0.49–1.45)	No significant association. Estimates were directionally inverse.

**Table 2:** Summary of Mendelian randomization evidence on genetically predicted Lp(a) and prostate cancer risk.

Study	Lp(a) Definition	Main IVW Estimate	Population and Database	Genetic Instrument	Adjusted Variables	Overall Finding
Ioannidou et al. [31]	Inverse-rank-normalised serum Lp(a); OR per 1-SD increase in genetically predicted Lp(a)	OR 1.07 (95% CI 0.91–1.25), *p* = 0.431	Lp(a): 167,020. Total PCa: 79,166 cases/61,106 controls; advanced PCa: 15,167/58,308; early-onset PCa: 6988/44,256. European ancestry. **UK Biobank—GWAS**	10 independent genome-wide significant SNPs; sensitivity analyses—28 externally identified SNPs and four cis-LPA variants	Exposure GWAS adjusted for age, age^2^, sex terms and 20 principal components. MVMR included HDL-C, LDL-C and triglycerides; additional models included BMI, liver enzymes or creatinine. Pleiotropy-sensitive estimators and exclusion of aspirin-associated variants were applied	Primary IVW analysis was null, but several alternative and multivariable analyses supported a modest positive association.
Fang et al. [44]	Inverse-rank-normalised Lp(a); OR per 1-SD increase	OR 1.06 (95% CI 0.95–1.20), *p* = 0.305	Lp(a): 167,020. PCa: 79,194 cases/61,112 controls. European ancestry. UK-**Biobank—GWAS**	15 independent genome-wide significant SNPs; additional analysis used the cis-LPA variant rs73596816	MVMR adjusted Lp(a) for LDL-C	Primary IVW analysis was null; weighted-median and multivariable analyses suggested a modest positive association.
Chen et al. [40]	Continuous genetically predicted Lp(a)	OR 1.06 (95% CI 1.02–1.11), *p* = 0.005	Lp(a): 273,896. European ancestry. **GWAS**	22 Lp(a)-associated SNPs before harmonisation; 11–20 instruments across cancer outcomes	-	Positive association between genetically predicted Lp(a) and PCa risk.
Lin et al. [43]	Continuous Lp(a); OR per 1-SD increase	OR 1.12, *p* = 0.038	Lp(a): 363,228. PCa: 15,199 cases/131,266 controls. European ancestry. **Uk biobank, Finngen**	13 independent genome-wide significant SNPs	-	Positive association in the reported forward MR analysis.
Li et al. [42]	Continuous Lp(a)	OR 1.12 (95% CI 1.01–1.25), *p* = 0.038	Lp(a): 363,228. PCa: 15,199 cases/131,266 controls. European ancestry. **Uk biobank, Finngen**	13 independent genome-wide significant SNPs; confounder-associated variants and MR-PRESSO outliers were screened	-	A modest positive association was supported across several MR methods.
An et al. [41]	Continuous Lp(a)	OR 1.12 (95% CI 1.00–1.24), *p* = 0.040	Lp(a): 363,228. Discovery: 15,199 cases/131,266 controls; validation: 79,148/61,106. European ancestry. **Uk biobank, Finngen**	20 independent SNPs in the discovery analysis	-	Directionally positive findings, with statistical significance varying across MR methods.

**Table 3:** Risk of bias assessment of included studies using design-specific tools.

ROBINS-E																										
Study	D1		D2				D3			D4			D5			D6			D7				Overall			
Bergeron et al. [38]	High		High				High			Some concerns			Some concerns			Low			Some concerns				High			
Katzke et al. [36]	Some concerns		Some concerns				Some concerns			Low			Some concerns			Some concerns			Low				Some concerns			
Marrer et al. [30]	Some concerns		Some concerns				Low			Some concerns			Low			Low			Some concerns				Some concerns			
Perez-Cornago et al. [37]	Some concerns		Some concerns				Some concerns			Low			Some concerns			Low			Some concerns				Some concerns			
**MR risk-of-bias tool adapted from Mamluk et al.**																										
Study	D1			D2				D3						D4			D5						Overall			
Ioannidou et al. [31]	Moderate risk			Low risk				Low risk						Moderate risk			Low risk						Moderate risk			
Fang et al. [44]	Low risk			Low risk				Low risk						Low risk			Low risk						Low risk			
Chen et al. [40]	Low risk			Moderate risk				Low risk						Moderate risk			Moderate risk						Moderate risk			
An et al. [41]	Low risk			Low risk				Low risk						Low risk			Low risk						Low risk			
Li et al. [42]	Low risk			Low risk				Low risk						Low risk			Low risk						Low risk			
Lin et al. [43]	Moderate risk			Moderate risk				Low risk						Moderate risk			Moderate risk						Moderate risk			
Q-Genie																										
Study	D1	D2			D3		D4		D5			D6		D7		D8		D9		D10		D11		Overall		
Mieno et al. [39]	6	6			5		5		4			5		5		5		6		5		6		58/77		
**QUIPS**																										
Study	D1					D2					D3				D4		D5				D6				Overall	
Wang et al. [14]	Moderate					Moderate					Moderate				Low		Low				High				Moderate	

ROBINS-E: D1- bias due to confounding; D2—bias arising from measurement of the exposure; D3—bias in selection of participants into the study or analysis; D4—bias due to post-exposure interventions; D5—bias due to missing data; D6—bias arising from measurement of the outcome; D7—bias in selection of the reported result. MR risk-of-bias tool adapted from Mamluk et al.: D1—weak instrument bias; D2—genetic confounding; D3—non-genetic confounding; D4—horizontal pleiotropy; D5—selection bias. Q-Genie: D1—rationale for the study; D2—selection and definition of the outcome of interest; D3—selection and comparability of comparison groups; D4—technical classification of the genetic exposure; D5—non-technical classification of the genetic exposure; D6—other sources of bias; D7—sample size and power; D8—a priori planning of analyses; D9—statistical methods and control for confounding; D10—testing of assumptions and inferences for genetic analyses; D11—appropriateness of inferences drawn from results. Each domain is scored from 1 to 7, with higher scores indicating better methodological quality. For studies with control groups, total scores ≤35 indicate poor quality, >35 to ≤45 indicate moderate quality, and >45 indicate good quality. For studies without control groups, total scores ≤32 indicate poor quality, >32 to ≤40 indicate moderate quality, and >40 indicate good quality. QUIPS: D1—study participation; D2—study attrition; D3—prognostic factor measurement; D4—outcome measurement; D5—study confounding; D6—statistical analysis and reporting.

**Table 4:** GRADE certainty assessment for evidence on Lp(a) exposure and prostate cancer outcomes.

Effect of Lp(a) Exposure on PCa	Evidence Group	Included Studies	Certainty
Incidence and mortality	Serum-based Lp(a) measurement	Katzke et al. [36], Perez-Cornago et al. [37], Marrer et al. [30]	Very low
Incidence	Mendellian randomization study	Ioannidou et al. [31], Fang et al. [44], Chen et al. [40], Lin et al. [43], Li et al. [42], An et al. [41]	Low
Incidence and mortality	Genetic study	Mieno et al. [39]	Very low
Clinicopathological features	Serum-based Lp(a) measurement	Wang et al. [14], Bergeron et al. [38]	Very low
Clinicopathological features	Mendellian randomization study	Ioannidou et al. [31]	Very low

## Data Availability

Extraction data is available in supplementary files.

## Supplementary Materials

The following supporting information can be downloaded at: https://www.mdpi.com/article/10.3390/ijms27188274/s1.
