# Supplementary material for: Lipoprotein(a) and Prostate Cancer: A Systematic Review of Observational, Genetic and Mendelian Randomization Evidence
Source: Int J Mol Sci. 2026 Sep 17;27(18):8274. doi: 10.3390/ijms27188274 (PMC13607466; doi:10.3390/ijms27188274)
Supplement: Supplementary file 1 [file ijms-27-08274-s001.zip › Supplementary Material S1.pdf]

Lp(a) search query

20.03.2026 – last re-run 20.05.2026

PubMed search query 20.03.2026 – last re-run 20.05.2026

("Lipoprotein(a)"[mh] OR "Lipoprotein(a)"[tiab] OR "lipoprotein a"[tiab] OR "lipoprotein(a)"[tiab] OR “Lp(a)”[tiab] OR “LPA”[tiab]) AND ("Prostatic Neoplasms"[Mesh] OR “prostate”[tiab] OR "prostate cancer"[tiab] OR "prostatic neoplasm*"[tiab] OR "prostate neoplasm*"[tiab] OR "prostate carcinoma"[tiab])

N=119

Scopus search query

(INDEXTERMS("Lipoprotein(a)") OR TITLE-ABS-KEY("Lipoprotein(a)" OR "lipoprotein a" OR "lipoprotein(a)" OR "Lp(a)" OR "LPA")) AND (INDEXTERMS("Prostatic Neoplasms") OR TITLE-ABS-KEY(prostate OR "prostate cancer" OR "prostatic neoplasm*" OR "prostate neoplasm*" OR "prostate carcinoma"))

N=196

Web of Science Core Collection search query

(AK=("Lipoprotein(a)") OR KP=("Lipoprotein(a)") OR TS=("Lipoprotein(a)" OR "lipoprotein a" OR "lipoprotein(a)" OR "Lp(a)" OR "LPA")) AND (AK=("Prostatic Neoplasms") OR KP=("Prostatic Neoplasms") OR TS=(prostate OR "prostate cancer" OR "prostatic neoplasm*" OR "prostate neoplasm*" OR "prostate carcinoma"))

N=174
