# Supplementary material for: Lipoprotein(a) and Prostate Cancer: A Systematic Review of Observational, Genetic and Mendelian Randomization Evidence
Source: Int J Mol Sci. 2026 Sep 17;27(18):8274. doi: 10.3390/ijms27188274 (PMC13607466; doi:10.3390/ijms27188274)
Supplement: Supplementary file 1 [file ijms-27-08274-s001.zip › Supplementary Material S10.pdf]

Modified risk-of-bias assessment tool for mendelian randomization studies adopted from Mamluk et al.

Bias domain	Question	High risk	Some concerns	Low risk
Weak instrument bias	Strength of association between the genetic instrument and Lp(a). Was instrument strength reported: F-statistic, genome-wide significance or other information supporting instrument relevance?	F-statistic <10. Weak or poorly justified association between SNPs and Lp(a). Instruments not clearly associated with Lp(a).	F-statistic missing or close to 10. Instrument strength incompletely reported, but variants are plausibly associated with Lp(a) or selected from prior GWAS.	F-statistic clearly >10, or variants are genome-wide significant and strongly associated with Lp(a). Independent SNPs or clearly justified genetic instruments used.
Genetic confounding bias	Reported test on association between confounders and IV<br><br>(testing the assumption that the instrument is associated with your outcome only via your exposure)	Clear evidence that instruments are associated with potential confounders.	Tests for confounder associations not presented or<br>Yes presented AND there is some degree of association	Presented and no obvious association
'Other' confounding bias	Included covariates in the MR analysis in a way that could introduce bias?	Inappropriate adjustment for variables that may be downstream of Lp(a). Colliders, or mediators without justification.	Adjustment strategy unclear, incompletely reported or includes covariates which role as confounders is uncertain.	No inappropriate covariate adjustment or multivariable MR adjustment is clearly justified and limited to relevant correlated lipid traits or other prespecified factors.
Additional direct effects between IV and outcome (exclusion restriction assumption)	Presence of horizontal pleiotropy for genetic instruments. Could the genetic variants affect prostate cancer through pathways other than Lp(a)?	Genetic instruments have no clear biological mechanism for Lp(a). No pleiotropy assessment. Evidence of horizontal pleiotropy.	Genetic instruments have plausible biological relationship with Lp(a), but pleiotropic pathways are possible. Limited pleiotropy testing.	Instruments are LPA region/cis variants or analyses include adequate pleiotropy checks: MR-Egger intercept, weighted median approach, MR-PRESSO, multivariable MR, sensitivity analyses, colocalization. No evidence of horizontal pleiotropy.
Bias due to selection of participants	Homogenous population or similar ancestry<br>If no<br><br>Stratified by ethnicity or adjusted for population stratification (yes/no)	Non-homogenous population (e.g. black and white together, etc.)	population described as homogenous (e.g. whites only) BUT not corrected for ancestry informative markers like principal components derived from GWAS.	population described as homogenous (e.g. whites only) AND corrected for ancestry informative markers like principal components derived from GWAS.

Assumptions: Assumption is that these studies when at low risk are equivalent to a low risk RCT

Abbreviations: IV - instrumental variable; MR - Mendelian randomization; Lp(a) - lipoprotein(a); PCa - prostate cancer; SNP - single nucleotide polymorphism. "Some concerns" corresponds to moderate risk in the original tool adapted from Mamluk et al.
