# Supplementary material for: Lipoprotein(a) and Prostate Cancer: A Systematic Review of Observational, Genetic and Mendelian Randomization Evidence
Source: Int J Mol Sci. 2026 Sep 17;27(18):8274. doi: 10.3390/ijms27188274 (PMC13607466; doi:10.3390/ijms27188274)
Supplement: Supplementary file 1 [file ijms-27-08274-s001.zip › Supplementary Material S11.pdf]

GRADE certainty assessment - full

Lp(a) exposure on prostate cancer	Evidence subgroup	Outcome assessed	Studies	Main evidence summary	Main GRADE concerns	Final certainty	Rationale
Incidence and mortality	Serum Lp(a) measurement	PCa incidence and PCa mortality	Katzke et al., Perez-Cornago et al., Marrer et al.	Katzke et al. reported higher PCa incidence in the highest vs lowest Lp(a) quartile. Perez-Cornago et al. reported little evidence of association with PCa incidence or death. Marrer et al. showed a non-significant inverse direction for PCa in the highest quartile. Overall direction was inconsistent and PCa-specific mortality evidence was sparse.	• RoB: serious - residual confounding, multiple biomarker comparisons<br>• Inconsistency: serious - positive, null, inverse directions.<br>• Indirectness: not serious to serious - prospective cohorts but different populations and exposure handling.<br>• Imprecision: serious - borderline or wide CIs; little data on PCa mortality.<br>• Reporting bias: undetected, cannot be excluded.	Very low	Low because evidence is observational. Downgraded for inconsistency and imprecision, with additional concern for residual confounding and multiple testing. Confidence in a true association is very limited.
Incidence and mortality	Mendellian randomization studies	Genetically predicted Lp(a) and PCa incidence/mortality generally not directly assessed	Ioannidou et al., Fang et al., Chen et al., Lin; Li; An	Most MR analyses have shown positive correlation between genetically predicted Lp(a) and PCa risk, with modest effect sizes. Stronger signals were reported in weighted-median, validation, or colocalization analyses. Several estimates were small in magnitude or did not remain significant under stricter correction in all studies.	• RoB: serious to not serious depending on study; concerns include weak instruments, pleiotropy, sample overlap, population stratification.<br>• Inconsistency: some concern - generally positive direction, but method-dependent.<br>• Indirectness: some concern - genetically predicted Lp(a), not measured circulating Lp(a); some studies had broader biomarker or drug-target aims.<br>• Imprecision: some concern - small effects; some CIs close to zero.<br>• Reporting bias - cannot be excluded.	Low	Low for MR evidence. Not downgraded to very low because direction was broadly concordant and several studies used sensitivity analyses. Certainty remains limited by MR assumptions and small effects.
Incidence and mortality	Genetic studies	LPAL2-LPA region - PCa incidence and mortality	Mieno et al.	No significant association was found between the selected LPAL2-LPA region SNPs and overall cancer incidence and mortality after correction.	• RoB: some concern - genetic cohort design; possible limited PCa events.<br>• Inconsistency: cannot assess - single study.<br>• Indirectness: serious - SNPs in region may not fully capture Lp(a) concentration or causal Lp(a) effect.<br>• Imprecision: serious - single study; suggestive estimates; multiple comparisons<br>• Reporting bias: cannot assess.	Very low	Low and downgraded for single-study evidence, indirectness of genetic proxies, and imprecision, multiple testing.
Clinicopathological features	Serum-based Lp(a) measurement	High-risk PCa, risk group based on PCa, stage, metastasis, localized high-grade PCa	Wang et al., Bergeron et al.	Wang reported higher Lp(a) in high-risk PCa and associations with adverse clinicopathological features after adjustment. Bergeron found no significant difference in Lp(a) between men with localized Gleason 8-9 PCa and risk matched controls. Evidence is limited to few studies with different populations and outcome definitions.	• RoB: serious - observational/single-center designs, residual confounding, selection of controls, patients).<br>• Inconsistency: serious - positive vs null findings.<br>• Indirectness: serious - different definitions of aggressive/high-grade disease and clinical settings.<br>• Imprecision: serious - few studies, one small matched cohort<br>• Reporting bias: cannot be excluded.	Very low	Low and downgraded for inconsistency. Evidence supports only a theoretical association with adverse features.
Clinicopathological features	Mendellian randomization studies	Aggressive or advanced PCa	Ioannidou et al.	Evidence for advanced PCa was weaker than for total PCa. The direction was generally positive, with stronger results in LPA-restricted analyses, but multivariable MR estimates were borderline and confidence intervals were close to zerol.	• RoB: some concern - unmeasured pleiotropy cannot be excluded.<br>• Inconsistency: not assessable across many studies for this subgroup, model-dependent within study.<br>• Indirectness: some concern - genetically predicted Lp(a), advanced PCa definitions.<br>• Imprecision: serious -borderline estimates; limited subgroup evidence.<br>• Reporting bias: not assessable.	Very low	Low and downgraded for imprecision and model-dependent subgroup evidence. The association with advanced or aggressive PCa should be considered very uncertain.
