# Supplementary material for: Lipoprotein(a) and Prostate Cancer: A Systematic Review of Observational, Genetic and Mendelian Randomization Evidence
Source: Int J Mol Sci. 2026 Sep 17;27(18):8274. doi: 10.3390/ijms27188274 (PMC13607466; doi:10.3390/ijms27188274)
Supplement: Supplementary file 1 [file ijms-27-08274-s001.zip › Supplementary Material S2.pdf]

The Risk Of Bias In Non-randomized Studies – of Exposures (ROBINS-E) assessment tool
(for follow-up studies)

Template for completion

Version 24 March 2024

This work is licensed under a Creative Commons Attribution-NonCommercial-NoDerivatives 4.0 International License.

The ROBINS-E tool

At planning stage: list confounding factors and consider appropriateness criteria

P1. List the important confounding factors relevant to all or most studies on this topic. Specify whether these are particular to specific exposures-outcome combinations.

Age, ethnicity or genetic ancestry, family history and inherited susceptibility to prostate cancer, socioeconomic position, access to health care, PSA testing and screening behaviour, BMI and adiposity, diabetes and metabolic health, smoking, alcohol intake, physical activity, diet, lipid lowering medication, cardiovascular disease and health care contact, liver disease, renal disease, inflammation, and endogenous hormone related factors. For case control biomarker studies, additional concerns include indication for prostate biopsy, negative biopsy status among controls, timing of blood sampling in relation to diagnosis, and differences between source biobanks.

P2. Will the review use the ROBINS-E assessment of appropriateness (important aspects of "study sensitivity")?

No

If Yes, complete sections Addressing appropriateness, Parts I and II in Appendix 1.

For each study result: preliminary considerations

A. Specify the result being assessed for risk of bias

A1. Specify the numerical result being assessed

Comparison of plasma Lp(a) concentration between 35 men with localized Gleason 8 or 9 prostate cancer and 35 at risk cancer free men matched by age and BMI. Main Lp(a) result from Table 1 and Figure 1: mean 84.5 nmol per L in cases compared with 57.1 nmol per L in at risk controls; median 23.0 nmol per L in cases compared with 25.0 nmol per L in controls; unpaired test p= 0.64 and paired Wilcoxon test p = 0.68. The assessment focuses on the paired comparison of Lp(a) levels and prostate cancer status.

B. Decide whether to proceed with a risk-of-bias assessment

	Response options	Comments
B1. Did the authors make any attempt to control for confounding?	Y	The study used individual matching by age and BMI. Paired tests were performed after matching. However, there was no multivariable adjustment for other important confounders.
B2. **If N/PN to B1**: Is there sufficient potential for confounding that an unadjusted result should not be considered further?	NA	Not applicable because the authors made an attempt to control for confounding through matching.
B3. Was the method of measuring exposure inappropriate?	N	Plasma Lp(a) was measured using a Roche Cobas analytical platform. Samples were processed using institutional standard operating procedures, stored at minus 80 degrees C, and thawed once on the assay day. This is a technically appropriate method for measuring circulating Lp(a), although it does not solve the timing concern.
B4. Was the method of measuring the outcome inappropriate?	N	The outcome classification was appropriate for the study design. Cases had localized high grade prostate cancer with Gleason 8 or 9 on biopsy. Controls had a negative prostate biopsy and remained cancer free for at least 3.2 years after biopsy and blood sampling.

If the answer to any of B2, B3 or B4 is 'Yes' or 'Probably yes', the result should be considered to be at very high risk of bias and no further assessment is required. Otherwise, proceed to section C.

C. Specify the analysis in the current study for which results are being assessed for risk of bias

C1. Specify the outcome to which this result relates.

Localized high grade prostate cancer status, defined as Gleason 8 or 9 prostate cancer, compared with at risk cancer free status.

C2. Specify the participant group on which this result was based.

70 men from 2 clinical biobanks in Quebec, Canada: 35 men with high grade localized prostate cancer from the URO 1 biobank and 35 cancer free men at risk of prostate cancer from the BIOCaPPE GRePEC biobank. Controls were investigated because of elevated PSA or positive family history, had a negative prostate biopsy, and remained cancer free for at least 3.2 years after biopsy and blood sampling. Cases and controls were individually matched by age and BMI category.

C3 to C8: Describe the exposure measurement(s) used to produce this result.

C3. What is the exposure being measured and how was it measured or assessed?	Plasma Lp(a) concentration measured from EDTA plasma samples. Lp(a) was measured on a Roche Cobas analytical platform at the Core laboratory of the Hopital de l Enfant Jesus in Quebec City. Values below the reportable range of 10 nmol/L were assigned a value of 3 nmol/L. The analysis compared Lp(a) levels as a continuous biomarker between matched groups.
C4. Was exposure analysed as a quantitative (rather than a categorical) variable?	Y
C5. Did repeated measurements of exposure over time (for each participant) contribute to the analysis that produced this result?	N
C6. **If Y/PY to C5**, was a single estimate of each participant’s exposure level derived from the repeated measurements of exposure over time?	NA
C7. **If N/PN to C6**, was the analysis based on splitting participants’ follow up time according to exposure status and/or magnitude?	NA
C8. **If Y/PY to C7**, were changes in exposure status and/or magnitude likely to be related to factors that are predictive of the outcome?	NA

C9. **If N/PN to C7**, how were repeat measurements used?

Not applicable. Lp(a) was measured once and no repeated measurements over time contributed to the comparison.

Y = Yes; PY = Probably yes; PN = Probably no; N = No; NA = Not applicable

C10. Specify the relationship analysed to produce this result. For example, this may be a quadratic relationship of cumulative exposure with the log odds of the outcome, or a risk ratio for the outcome comparing exposed with unexposed individuals.

Paired comparison of plasma Lp(a) concentration between men with localized Gleason 8 or 9 prostate cancer and age and BMI matched at risk cancer free men. The main result was a non significant paired Wilcoxon test for Lp(a), p = 0.68, with medians of 23.0 and 25.0 nmol/L in cases and controls, respectively.

D: Specify the causal effect of exposure being estimated by this result

D1. Specify the population of interest	Describe eligible participants (to whom the causal effect applies). These may be different from the study participants on whom the result was based (specified in C2). Such differences may give rise to selection biases.	Adult men at risk of prostate cancer who are comparable to the study participants and who could undergo prostate cancer evaluation. The review question of interest is whether higher usual Lp(a) concentrations are linked to prostate cancer risk or to adverse prostate cancer features.
Specification of the exposure metric of interest		
D2. Specify the exposure	This is the factor whose causal effect on the outcome of interest is the subject of the study result being assessed. It may be thought of as the 'true' exposure of interest. It is distinct from the method with which exposure was measured.	Higher usual circulating Lp(a) concentration before the development or clinical detection of prostate cancer.
D3. Specify the exposure window	The exposure window of interest is the exposure period for which the result being assessed estimates the effect of exposure on the outcome. Specification of the exposure window is judged by the ROBINS-E user, who should aim to define a window that is both meaningful in answering the review question and broadly in line with when the study measured exposure. Specification should include both the time of onset and period of exposure. For example, it may be lifetime exposure (from birth or from conception), during ages 50-55, the period from first employment in a particular occupation, time from birth to age 10, or during pregnancy.<br><br>The specified exposure window is used to determine whether exposure data adequately reflect exposure during the window. Exposure before the start of the exposure window is addressed during the assessment of risk of bias due to confounding	The exposure window of interest is usual circulating Lp(a) before prostate cancer diagnosis or before confirmation of cancer free status. In this study, Lp(a) was measured at the time of clinical evaluation or near diagnosis rather than clearly before disease development.
D4. Specify how exposure over time	This may, for example, be ever/never exposed, cumulative exposure, average exposure, or peak exposure during the exposure period, for each participant. Alternatively, there may be only a	Usual circulating plasma Lp(a) concentration, analysed as a continuous biomarker in the matched group comparison. Because Lp(a) is largely genetically determined, a single measurement may approximate usual

should be summarized	single exposure event, or the exposure may be time invariant (such as a genetic variant or family history).	concentration, but the timing of measurement remains a major interpretive limitation.

E. Evaluation of confounding factors

Complete a row for each important confounding factor listed in advance (subsection (i)). In addition, consider any further confounding factors that are either relevant to the setting of this particular study or which the study authors identified as potentially important (subsection (ii)).

"Important" confounding factors are those for which, in the context of this study, adjustment is expected to lead to an important change in the estimated effect of the exposure.

(i) Important confounding factors listed in advance						
Confounding factor	Measured variable(s) for this factor, if any	Was this variable (or were these variables) controlled for in the analysis? (Y / N)	If this confounding factor was controlled for, was it measured validly and reliably by this variable (or these variables)?* (NA / Y / PY / PN / N / NI)	If this confounding factor was not controlled for, is there evidence that controlling for it was unnecessary?** (NA / Y / PY / PN / N)	Is failure to adjust for this confounding factor expected to bias the effect estimate towards benefit or harm of (higher) exposure?*** (Benefit of (higher) exposure / Harm of (higher) exposure / Insufficient information available)	Comments
Age	Age in years	Y	Y	NA	Insufficient information	Cases and controls were individually matched by age. Almost all pairs had the same age, with two pairs differing by four and five years.
BMI and adiposity	BMI category and measured BMI	Y	Y	NA	Insufficient information	Cases and controls were individually matched by BMI category. The maximum BMI difference within a pair was 3.25 kg per m2.
Ethnicity or genetic ancestry	Not reported	N	NA	N	Insufficient information	Genetic ancestry is important for Lp(a) concentration. The paper did not report ancestry or control for it.

Family history and inherited susceptibility	Positive family history was part of control eligibility for some controls	N	NA	N	Insufficient information	Controls were selected partly because of positive family history or elevated PSA, but family history was not described for cases or adjusted in the analysis.
PSA testing and biopsy indication	Elevated PSA status and negative biopsy in controls; PSA level measured in both groups	N	NA	N	Insufficient information	Control selection depended on elevated PSA or family history, followed by negative biopsy. PSA and biopsy indication may be related to diagnosis and could be linked to lipid metabolism or medical surveillance. The analysis did not adjust for this selection mechanism.
Socioeconomic position and access to care	Not reported	N	NA	N	Insufficient information	Access to screening, biopsy, and specialist care may influence prostate cancer diagnosis. No direct adjustment was reported.
Smoking	Not reported	N	NA	N	Insufficient information	Smoking may be associated with metabolic markers and prostate cancer outcomes. It was not reported or adjusted.

Alcohol intake	Not reported	N	NA	N	Insufficient information	Alcohol intake may influence lipid markers and general health. It was not reported or adjusted.
Diabetes and metabolic health	Not reported	N	NA	N	Insufficient information	Diabetes and metabolic health may influence cancer detection and lipid markers. No adjustment was reported.
Lipid lowering medication	Not reported as an analysis variable	N	NA	N	Insufficient information	The article discusses lipid lowering drugs as relevant to prostate cancer and lipid markers, but use of statins or other lipid lowering medication was not reported as a covariate in the comparison.
Hormone related medication	Use of 5 alpha reductase inhibitors and alpha blockers	N	NA	N	Insufficient information	Six men with prostate cancer were taking 5 alpha reductase inhibitors, and alpha blocker use was also reported. These variables were not incorporated into the Lp(a) group comparison.

Renal disease, liver disease, and inflammation	Not reported	N	NA	N	Insufficient information	These conditions can affect lipid biomarkers or cancer related health status. They were not reported or controlled.

(ii) Additional confounding factors relevant to the setting of this particular study, or identified by study authors and considered to be important, or which were identified since the protocol was written						
Confounding factor	Measured variable(s) for this factor, if any	Was this variable (or were these variables) controlled for in the analysis? (Y / N)	If this confounding factor was controlled for, was it measured validly and reliably by this variable (or these variables)?* (NA / Y / PY / PN / N / NI)	If this confounding factor was not controlled for, is there evidence that controlling for it was unnecessary?** (NA / Y / PY / PN / N)	Is failure to adjust for this confounding factor expected to bias the effect estimate towards benefit or harm of (higher) exposure?*** (Benefit of (higher) exposure / Harm of (higher) exposure / Insufficient information available)	Comments
Source biobank	URO 1 for cases and BIOCaPPE GRePEC for controls	N	NA	N	Insufficient information	Cases and controls came from different clinical biobanks. Differences in recruitment, sample availability, referral pathways, and clinical context could influence biomarker distributions.
Timing of blood sampling in relation to disease	Blood sampling date between 2010 and 2019; diagnosis status at sampling	N	NA	N	Insufficient information	For cases, Lp(a) was measured in men already diagnosed with localized high grade prostate cancer or during the diagnostic period. This weakens causal interpretation and raises concern about reverse causation.

Fasting status	Fasting status reported	N	NA	PY	Insufficient information	All samples were fasting except one participant in each group. This is unlikely to materially affect Lp(a), but it was not adjusted.
Sample handling and storage period	EDTA plasma, storage at minus 80 degrees C, thawed once	N	NA	PN	Insufficient information	Handling was described, but storage duration may have varied because blood was drawn from 2010 to 2019. There is no clear evidence that this caused bias in Lp(a).

* “Validity” refers to whether the confounding variable or variables accurately measure the confounding factor, while “reliability” refers to the precision of the measurement (more measurement error means less reliability).

** In the context of a particular study, variables need not be included in the analysis: (a) if they are measured validly and reliably and are not associated with the outcome, conditional on exposure (noting that lack of a statistically significant association is not evidence of a lack of association); (b) if they are measured validly and reliably and are not associated with exposure; (c) if they are measured validly and reliably and adjustment makes no or minimal difference to the estimated effect of the primary parameter; (d) because the confounder was addressed in the study design, for example by restricting to individuals with the same value of the confounder; (e) because a negative control demonstrates that there was unlikely to have been confounding due to this variable or that uncontrolled confounding was likely to be minimal; or (f) because external evidence suggests that controlling for the variable is not necessary in the context of the study being assessed..

For each study: risk of bias assessment

Domain 1: Risk of bias due to confounding

Domain 1, Variant (a): ***If N/PN to C5 or Y/PY to C6 or N/PN to C7*** *(only baseline confounding needs to be addressed)*

Signalling questions	Response options	Comments
1.1 Did the authors control for all the important confounding factors for which this was necessary?	SN	The authors matched on age and BMI, but did not control for several important confounders and selection factors, including ancestry, family history, PSA testing and biopsy indication, health care access, smoking, alcohol intake, diabetes, lipid lowering medication, and source biobank. Uncontrolled confounding is probably substantial for a causal interpretation.
1.2 **If Y/PY/WN to 1.1**: Were confounding factors that were controlled for (and for which control was necessary) measured validly and reliably by the variables available in this study?	WN	Age and BMI were measured adequately. However, only these factors were controlled. The main concern is not measurement error in these two variables, but absence of control for several other important factors.
1.3 **If Y/PY/WN to 1.1**: Did the authors control for any variables after the start of the exposure period being studied that could have been affected by the exposure?	NA	No variables after the start of the exposure period were controlled in a multivariable analysis. Matching was based on age and BMI.
1.4 Did the use of negative controls, or other considerations, suggest serious uncontrolled confounding?	PN	No negative control analysis was reported. The observed strong differences in PSA and differences in clinical source suggest that uncontrolled confounding and selection are plausible, but there is no formal negative control evidence.
Risk of bias (due to confounding) in the estimated effect of exposure on the outcome	High risk	Risk of bias due to confounding is high. Matching by age and BMI was useful, but insufficient for the review question because important factors related to Lp(a), prostate cancer diagnosis, and clinical evaluation were not addressed.
What is the predicted direction of bias due to confounding?	Insufficient information available	The direction of confounding is difficult to predict because control selection by elevated PSA or family history could influence the comparison in different ways.

Signalling questions	Response options	Comments
Is the risk of bias (due to confounding) sufficiently high, in the context of its likely direction and the magnitude of the estimated exposure effect, to threaten conclusions about whether the exposure has an important effect on the outcome?	Yes	The confounding concern is sufficiently important to threaten causal interpretation of the Lp(a) and prostate cancer status comparison.

Y = Yes; PY = Probably yes; PN = Probably no; N = No; SY = Strong yes; WY = Weak yes; SN = Strong no; WN = Weak no; NA = Not applicable; NI = No information

*Domain 1, variant (b): **If Y/PY to C7 and Y/PY to C8** (the analysis was based on splitting participants' follow up time according to exposure status and/or magnitude and changes in exposure status and/or magnitude likely to be related to factors that are predictive of the outcome, so both baseline and time-varying confounding need to be addressed)*

Signalling questions	Response options	Comments
1.1 Did the authors use an analysis method that was appropriate to control for time-varying as well as baseline confounding?	NA	Not applicable. The analysis used a single Lp(a) measurement and did not split follow up time according to changing exposure status.
1.2 **If Y/PY to 1.1**: Did the authors control for all the important baseline and time-varying confounding factors for which this was necessary?	NA	
1.3 **If Y/PY/WN to 1.2**: Were confounding factors that were controlled for (and for which control was necessary) measured validly and reliably by the variables available in this study?	NA	
1.4 **If N/PN/NI to 1.1**: Did the authors control for time-varying factors or other variables measured after the start of the exposure window being studied?	NA	
1.5 Did the use of negative controls, or other considerations, suggest uncontrolled confounding?	NA	
Risk of bias (due to confounding) in the estimated effect of exposure on the outcome	NA	
What is the predicted direction of bias due to confounding?	NA	
Is the risk of bias (due to confounding) sufficiently high, in the context of its likely direction and the magnitude of the estimated exposure effect, to threaten conclusions about whether the exposure has an important effect on the outcome?	NA	

Y = Yes; PY = Probably yes; PN = Probably no; N = No; SY = Strong yes; WY = Weak yes; SN = Strong no; WN = Weak no; NA = Not applicable; NI = No information

Domain 2: Risk of bias arising from measurement of the exposure

Domain 2, Variant (a): ***If N/PN to C5*** *(exposure was measured at a single point in time)*

Signalling questions	Response options	Comments
Mismeasurement or misclassification of the exposure.		
2.1 Does the measured exposure well-characterize the exposure metric specified to be of interest in this study? [*This was specified in the answers to D2, D3 and D4*]	WN	The Roche Cobas Lp(a) measurement probably reflects circulating Lp(a) at the time of blood sampling, but it does not clearly characterize usual prediagnostic Lp(a), which is the exposure metric most relevant to whether Lp(a) influences prostate cancer risk. For cases, blood was collected in men with known or newly diagnosed high grade prostate cancer.
2.2 Was the exposure likely to be measured with error, or misclassified?	WY	Technical measurement error was probably limited because samples were processed by standard procedures, stored at minus 80 degrees C, thawed once, and analysed on an established platform. Some error may arise from assigning values below 10 nmol/L to 3 nmol/L, and from limited detail about apo(a) isoform sensitivity.
Bias in the estimated effect of exposure arising from mismeasurement or misclassification of the exposure		
2.3 If SY/WY to 2.2: Could mismeasurement or misclassification of exposure have been differential (i.e. related to the outcome or risk of the outcome)?	WY	Differential exposure measurement is possible to a small extent because cases and controls came from different biobanks and may have differed in timing of blood collection and clinical context. However, the laboratory assay itself was unlikely to be influenced by knowledge of case status if samples were processed together.
2.4 If SY/WY to 2.2 and N/PN/WY to 2.3: Is non-differential measurement error likely to bias the estimated effect of exposure on outcome?	WY	Non differential measurement error and lower limit handling could reduce precision and bias comparisons towards no difference. The main issue is the timing of measurement rather than laboratory error.
Risk of bias (arising from measurement of exposure) in the estimated effect of exposure on the outcome	High risk	Risk of bias from exposure measurement is high for the causal review question because Lp(a) was measured at or near the diagnostic period rather than clearly before prostate cancer developed. Although Lp(a) is relatively stable, cancer related metabolic changes and clinical context cannot be excluded.
What is the predicted direction of bias arising from measurement of exposure?	Towards null	Laboratory error and high variability would probably reduce the ability to detect a true difference, but timing related bias could act in either direction.

Signalling questions	Response options	Comments
Is the risk of bias (arising from measurement of exposure) sufficiently high, in the context of its likely direction and the magnitude of the estimated exposure effect, to threaten conclusions about whether the exposure has an important effect on the outcome?	Yes	The timing and clinical context of exposure measurement are important enough to threaten causal interpretation.

Y = Yes; PY = Probably yes; PN = Probably no; N = No; SY = Strong yes; WY = Weak yes; SN = Strong no; WN = Weak no; NA = Not applicable; NI = No information

*Domain 2, Variant (b): **If Y/PY to C5 and Y/PY to C6** (each individual's exposure level was estimated from measurements made at multiple time points)*

Signalling questions	Response options	Comments
2.1 Does the measured exposure (derived from measurements at multiple time points) well-characterize the exposure metric specified to be of interest in this study? [*This was specified in the answers to D2, D3 and D4*]	NA	Not applicable. Lp(a) was measured at one time point only.
2.2 Was there error in measurement, or misclassification, of the exposure, at each single time point?	NA	
2.3 If SY/WY to 2.2: Could mismeasurement or misclassification of exposure have been differential (i.e. related to the outcome or risk of the outcome)?	NA	
2.4 If SY/WY to 2.2 and N/PN/WY to 2.3: Is the nature of the (non-differential) measurement error likely to bias the estimated effect of exposure on outcome?	NA	
Risk of bias (arising from measurement of exposure) in the estimated effect of exposure on the outcome	NA	
What is the predicted direction of bias arising from measurement of exposure?	NA	
Is the risk of bias (arising from measurement of exposure) sufficiently high, in the context of its likely direction and the magnitude of the estimated exposure effect, to threaten conclusions about whether the exposure has an important effect on the outcome?	NA	

Y = Yes; PY = Probably yes; SN = Strong no; WN = Weak no; NA = Not applicable; NI = No information

*Domain 2, Variant (c): **If Y/PY to C5, N/PN to C6 and Y/PY to C7** (the analysis was based on splitting participants' follow up time according to exposure status and/or magnitude):*

Signalling questions	Response options	Comments
2.1 Does the measured exposure (including changes over time) well-characterize the exposure metric specified to be of interest in this study? [*This was specified in the answers to D2, D3 and D4*]	NA	Not applicable. Lp(a) was measured at one time point only.
2.2 Was there error in measurement, or misclassification, of the exposure, at each single time point?	NA	
2.3 If SY/WY to 2.2: Could mismeasurement or misclassification of exposure have been differential (i.e. related to the outcome or risk of the outcome)?	NA	
2.4 If SY/WY to 2.2 and N/PN/WY to 2.3: Is the nature of the (non-differential) measurement error likely to bias the estimated effect of exposure on outcome?	NA	
Risk of bias (arising from measurement of exposure) in the estimated effect of exposure on the outcome	NA	
What is the predicted direction of bias arising from measurement of exposure?	NA	
Is the risk of bias (arising from measurement of exposure) sufficiently high, in the context of its likely direction and the magnitude of the estimated exposure effect, to threaten conclusions about whether the exposure has an important effect on the outcome?	NA	

Y = Yes; PY = Probably yes; SN = Strong no; WN = Weak no; NA = Not applicable; NI = No information

Domain 3: Risk of bias in selection of participants into the study (or into the analysis)

Signalling questions	Response options	Comments
3.1 Did follow-up begin at (or close to) the start of the exposure window for most participants? [*The exposure window is specified in D3*]	N	Follow up did not begin at the start of the exposure window. This was a case control biomarker comparison. Cases already had localized Gleason 8 or 9 prostate cancer, and controls were selected after negative biopsy and at least 3.2 years without cancer.
3.2 **If N/PN to 3.1**: Is the effect of exposure likely to be constant over the period of follow up analysed?	PN	For an etiologic question, the effect of Lp(a) cannot be assumed constant over the period before diagnosis, particularly because disease development, PSA testing, and clinical selection could occur before blood sampling.
3.3 Was selection of participants into the study (or into the analysis) based on participant characteristics observed after the start of the exposure window being studied? [*The exposure window is specified in D3*]	Y	Selection into the study was based on diagnosis status, biopsy findings, source biobank, and availability of plasma samples. These characteristics were observed after the start of the relevant prediagnostic exposure period.
3.4 **If Y/PY to 3.3**: Were these characteristics likely to be influenced by exposure or a cause of exposure?	PY	Diagnosis status and biopsy investigation may be influenced by causes of exposure such as ancestry, metabolic health, health care contact, family history, and PSA testing behaviour. Direct influence by Lp(a) is uncertain.
3.5 **If Y/PY to 3.4**: Were these characteristics likely to be influenced by the outcome or a cause of the outcome?	Y	The selection characteristics were directly determined by the outcome or by diagnostic processes leading to outcome classification. Cases were selected because they had Gleason 8 or 9 prostate cancer; controls because they had negative biopsy and remained cancer free.
3.6 **If N/PN to 3.2 or Y/PY to 3.5**: Is it likely that the analysis corrected for all of the potential selection biases identified in A and B above?	PN	The analysis did not correct for selection bias arising from different source biobanks, different case and control definitions, or clinical selection mechanisms.
3.7 **If N/PN to 3.2 or Y/PY to 3.5**: Did sensitivity analyses demonstrate that the likely impact of the potential selection biases identified in A or B above was minimal?	WN	No sensitivity analysis was reported to show that selection from different biobanks or control selection among at risk biopsy negative men had minimal impact.
Risk of bias (due to selection of participants into the study) in the estimated effect of exposure on the outcome	High risk	Risk of bias due to selection is high. The comparison is based on selected clinical biobank samples rather than a cohort sampled before disease onset, and cases and controls were selected using different clinical pathways.

Signalling questions	Response options	Comments
What is the predicted direction of bias<br>due to selection of participants into the study?	Insufficient information available	The direction of selection bias is uncertain. At risk controls may differ from the source population of cases in ways that could either increase or decrease Lp(a) differences.
Is the risk of bias (due to selection of participants into the study) sufficiently high, in the context of its likely direction and the magnitude of the estimated exposure effect, to threaten conclusions about whether the exposure has an important effect on the outcome?	Yes	Selection concerns are sufficiently important to threaten causal interpretation.

Y = Yes; PY = Probably yes; PN = Probably no; N = No; SN = Strong no; WN = Weak no; NA = Not applicable; NI = No information

Domain 4: Risk of bias due to post-exposure interventions

**Signalling questions**	**Response options**	**Comments**
4.1 Were there post-exposure interventions that were influenced by prior exposure during the follow-up period?	PY	PSA testing, prostate biopsy, diagnostic workup, 5 alpha reductase inhibitor use, alpha blocker use, and possibly lipid lowering therapy could have occurred during the relevant period before or around diagnosis. These factors could influence diagnosis or biomarker interpretation.
4.2 **If Y/PY to 4.1**: Is it likely that the analysis corrected for the effect of post-exposure interventions that were influenced by prior exposure?	PN	The comparison did not correct for diagnostic pathways, medication use, or treatment related factors. Only age and BMI matching were used.
Risk of bias (due post-exposure interventions) in the estimated effect of exposure on the outcome	Some concerns	There is some concern that diagnostic procedures and medication use around the exposure and outcome assessment period could influence group definition and interpretation. This concern overlaps with selection and confounding.
What is the predicted direction of bias due to confounding?	Insufficient information	The likely direction of bias cannot be predicted.
Is the risk of bias (due post-exposure interventions) sufficiently high, in the context of its likely direction and the magnitude of the estimated exposure effect, to threaten conclusions about whether the exposure has an important effect on the outcome?	Cannot tell	The domain contributes to concern, although the major threats are confounding, exposure timing, and selection.

Y = Yes; PY = Probably yes; PN = Probably no; N = No; NA = Not applicable; NI = No information

Domain 5: Risk of bias due to missing data

Signalling questions	Response options	Comments
5.1 Were complete data on exposure status available for all, or nearly all, participants?	Y	Lp(a) data were reported for all 70 included participants.
5.2 Were complete data on the outcome available for all, or nearly all, participants?	Y	Outcome status was reported for all included participants: 35 had Gleason 8 or 9 prostate cancer and 35 were at risk controls with negative biopsy and at least 3.2 years cancer free follow up.
5.3 Were complete data on confounding variables available for all, or nearly all, participants?	PN	Age and BMI were complete and used for matching. However, many important confounders were not measured or not reported, including ancestry, smoking, alcohol intake, diabetes, lipid lowering medication, and socioeconomic position.
5.4 **If N/PN/NI to 5.1, 5.2 or 5.3**: Is the result based on a complete case analysis?	PY	The analysis appears to be based on complete data for the selected matched sample. The paper does not fully describe how many eligible biobank participants were screened or excluded before the final 35 pairs were selected.
5.5 **If Y/PY/NI**: Was exclusion from the analysis because of missing data (in exposure, confounders or the outcome) likely to be related to the true value of the outcome?	NI	There is no information on whether exclusion before selection into the final matched sample was related to true outcome status or Lp(a).
5.6 **If N/PN to 5.5**: Were all or most predictors of missingness (in exposure, confounders or the outcome) included in the analysis model?	PN	Predictors of missingness or selection into the final matched sample were not included in a model.
5.7 **If N/PN to 5.4**: Was the analysis based on imputing missing values?	N	No imputation was reported.
5.8 **If Y/PY to 5.7**: Was imputation performed appropriately?	NA	Not applicable because no imputation was reported.
5.9 **If N/PN to 5.7**: Was an appropriate alternative method used to correct for bias due to missing data?	WN	No alternative method was used to correct for missing or unavailable data, but within the final selected sample exposure and outcome data were complete.
5.10 **If PN/N/NI to 5.1, 5.2 or 5.3**: Is there evidence that the result was not biased by missing data?	PN	There is limited evidence that missing data within the selected sample biased the result, but insufficient information on the sampling frame and sample availability before selection remains.

Signalling questions	Response options	Comments
Risk of bias (due to missing data) in the estimated effect of exposure on the outcome	Some concerns	Risk of bias due to missing data is judged as some concerns. Data were complete for included participants, but the report does not clearly describe eligible sample counts, unavailable samples, or exclusions before forming the matched groups.
What is the predicted direction of bias due to missing data?	Insufficient information available	The direction of any bias from missing or unavailable data cannot be predicted.
Is the risk of bias (due to missing data) sufficiently high, in the context of its likely direction and the magnitude of the estimated exposure effect, to threaten conclusions about whether the exposure has an important effect on the outcome?	Cannot tell	Missing data concerns alone are not the main threat, but incomplete reporting of the selection process adds uncertainty.

Y = Yes; PY = Probably yes; PN = Probably no; N = No; SY = Strong yes; WY = Weak yes; NA = Not applicable; NI = No information

Domain 6: Risk of bias arising from measurement of the outcome

**Signalling questions**	**Response options**	**Comments**
6.1 Could measurement or ascertainment of the outcome have differed between exposure groups or levels of exposure?	PN	Outcome classification was based on biopsy confirmed Gleason 8 or 9 prostate cancer for cases and negative prostate biopsy plus at least 3.2 years cancer free follow up for controls. The method is unlikely to differ by measured Lp(a), because Lp(a) was not part of the diagnostic workup.
6.2 Were outcome assessors aware of study participants' exposure history?	PN	Outcome status was established clinically before or independently of the research Lp(a) assay. Outcome assessors were unlikely to be influenced by Lp(a) levels.
6.3 **If Y/PY/NI to 6.2**: Could assessment of the outcome have been influenced by knowledge of participants' exposure history?	NA	Not applicable because outcome assessment was probably not influenced by knowledge of Lp(a).
Risk of bias (arising from measurement of outcomes) in the estimated effect of exposure on the outcome	Low risk	Outcome measurement was valid for distinguishing localized high grade prostate cancer from biopsy negative at risk controls. The control definition included follow up to reduce missed cancer classification.
What is the predicted direction of bias arising from measurement of outcomes?	Insufficient information available	No important outcome measurement bias is expected, although controls may still differ from the population that gave rise to cases.
Is the risk of bias (arising from measurement of outcomes) sufficiently high, in the context of its likely direction and the magnitude of the estimated exposure effect, to threaten conclusions about whether the exposure has an important effect on the outcome?	No	Outcome measurement is unlikely to threaten the conclusion.

Y = Yes; PY = Probably yes; PN = Probably no; N = No; SY = Strong yes; WY = Weak yes; NA = Not applicable; NI = No information

Domain 7: Risk of bias in selection of the reported result

Signalling questions	Response options	Comments
7.1 Was the result reported in accordance with an available, pre-determined analysis plan?	PY	The study objective and sample size calculation were reported. The analysis plan described group comparisons and correlation analyses, but a detailed prespecified protocol for all biomarkers and all comparisons was not provided in the article.
7.2 **If N/PN/NI to 7.1**: Is the reported effect estimate likely to be selected, based on desirability of the magnitude (or statistical significance) of the estimated effect of exposure on outcome, from multiple *exposure measurements* within the exposure domain?	PN	The Lp(a) result was clearly reported even though it was not statistically significant. Table 1 and Figure 1 provide the group comparison.
7.3 Is the reported effect estimate likely to be selected, based on desirability of the magnitude (or statistical significance) of the estimated effect of exposure on outcome, from multiple *outcome measurements* within the outcome domain?	PN	The prostate cancer group definition was central to the study aim and does not appear to have been selected based on the Lp(a) result.
7.4 Is the reported effect estimate likely to be selected, based on desirability of the magnitude (or statistical significance) of the estimated effect of exposure on outcome, from multiple *analyses* of the exposure-outcome relationship?	PY	The study examined multiple lipid markers, PCSK9, ANGPTL3, Lp(a), PSA correlations, and subgroup correlation matrices. No adjustment for multiple comparisons was reported for these exploratory analyses.
7.5 Is the reported effect estimate likely to be selected, based on the basis of desirability of the results (e.g. statistical significance), from different *subgroups*?	PY	The main Lp(a) comparison was straightforward, but the study included several analytical choices including paired and unpaired tests, multiple biomarkers, and multiple correlation analyses. The risk of selective emphasis is therefore possible.
Risk of bias (due to selection of the reported result) in the estimated effect of exposure on the outcome	Some concerns	There are some concerns due to the exploratory nature of the analysis and multiple comparisons. Concern is reduced because the null Lp(a) comparison was reported transparently.
What is the predicted direction of bias due to selection of the reported result?	Insufficient information available	Any bias from selective reporting is difficult to predict.

Signalling questions	Response options	Comments
Is the risk of bias (due to selection of the reported result) sufficiently high, in the context of its likely direction and the magnitude of the estimated exposure effect, to threaten conclusions about whether the exposure has an important effect on the outcome?	No	Selective reporting concerns are not sufficient alone to invalidate the reported Lp(a) comparison.

Y = Yes; PY = Probably yes; PN = Probably no; N = No; NA = Not applicable; NI = No information

Overall risk of bias

	Response options	Comments
Overall risk of bias	High risk	Overall judgement: High risk of bias. The study has strengths, including careful sample handling, direct Lp(a) measurement, age and BMI matching, biopsy confirmed high grade prostate cancer, and biopsy negative controls with follow up. However, for the systematic review question about whether higher Lp(a) concentrations are linked to prostate cancer risk or adverse features, the evidence is limited by major concerns about temporality, reverse causation, selection of cases and controls from different biobanks, lack of adjustment for important confounders, small sample size, and exploratory analysis. The study is useful as a biomarker comparison, but it provides weak causal evidence about Lp(a) and prostate cancer risk.
What is the predicted direction of bias?	Insufficient information available	Laboratory measurement error would probably bias towards no difference, but selection, diagnostic pathway, and confounding could act in either direction.
Is the overall risk of bias sufficiently high, in the context of its likely direction and the magnitude of the estimated exposure effect, to threaten conclusions about whether the exposure has an important effect on the outcome?	Yes	The risk of bias is sufficiently high to affect conclusions about whether Lp(a) has an important effect on prostate cancer risk. The study should be interpreted as exploratory supportive evidence only, not as strong etiologic evidence.

This work is licensed under a Creative Commons Attribution-NonCommercial-NoDerivatives 4.0 International License.
