# Supplementary material for: Lipoprotein(a) and Prostate Cancer: A Systematic Review of Observational, Genetic and Mendelian Randomization Evidence
Source: Int J Mol Sci. 2026 Sep 17;27(18):8274. doi: 10.3390/ijms27188274 (PMC13607466; doi:10.3390/ijms27188274)
Supplement: Supplementary file 1 [file ijms-27-08274-s001.zip › Supplementary Material S3.pdf]

The Risk Of Bias In Non-randomized Studies – of Exposures (ROBINS-E) assessment tool
(for follow-up studies)

Template for completion

Version 24 March 2024

This work is licensed under a Creative Commons Attribution-NonCommercial-NoDerivatives 4.0 International License.

The ROBINS-E tool

At planning stage: list confounding factors and consider appropriateness criteria

P1. List the important confounding factors relevant to all or most studies on this topic. Specify whether these are particular to specific exposures-outcome combinations.

The important confounding factors specified in advance are: age, sex where applicable (not applicable for prostate cancer analysis), ethnicity, family history, socioeconomic status, healthcare access, PSA testing behavior, BMI, waist circumference, smoking, alcohol intake, dietary pattern, physical activity, diabetes, metabolic health, hypertension, cardiovascular disease-related healthcare contact, lipid lowering medication, liver or kidney disease or other conditions that could affect circulating Lp(a). Confounding by PSA screening or healthcare access is particularly important for incident prostate cancer because it can influence detection. Ethnicity is important for Lp(a) because its distributions differ by ancestry.

P2. Will the review use the ROBINS-E assessment of appropriateness (important aspects of “study sensitivity”)?

No

If Yes, complete sections Addressing appropriateness, Parts I and II in Appendix 1.

For each study result: preliminary considerations

A. Specify the result being assessed for risk of bias

A1. Specify the numerical result being assessed

Adjusted Prentice-weighted Cox regression estimate for incident prostate cancer comparing highest versus lowest quartile of baseline circulating Lp(a) concentration: Q4 vs Q1 HR 1.43, 95% CI 1.02 to 2.03.

B. Decide whether to proceed with a risk-of-bias assessment

	Response options	Comments
B1. Did the authors make any attempt to control for confounding?	Y	The authors fitted Prentice-weighted Cox models and reported crude and multivariable-adjusted hazard ratios (HRs). The multivariable model adjusted for age/sex where applicable and multiple lifestyle, anthropometric, medical-history and medication variables.
B2. **If N/PN to B1**: Is there sufficient potential for confounding that an unadjusted result should not be considered further?	NA	Not applicable because the authors made an attempt to control for confounding.
B3. Was the method of measuring exposure inappropriate?	N	Lp(a) was measured in baseline prediagnostic serum using the Roche Cobas 6000 clinical chemistry system according to manufacturer protocols.
B4. Was the method of measuring the outcome inappropriate?	N	Incident prostate cancer was prospectively ascertained and verified using active follow-up plus pathology registry linkage and ICD-10 coding (C61).

If the answer to any of B2, B3 or B4 is 'Yes' or 'Probably yes', the result should be considered to be at very high risk of bias and no further assessment is required. Otherwise, proceed to section C.

C. Specify the analysis in the current study for which results are being assessed for risk of bias

C1. Specify the outcome to which this result relates.

Incident prostate cancer, defined as verified first incident prostate cancer during follow-up, coded ICD-10 (C61).

C2. Specify the participant group on which this result was based.

Men from the EPIC-Heidelberg prospective cohort included in the case-cohort analysis: incident prostate cancer cases diagnosed up to the end of 2012 and the randomly selected reference pool with available baseline blood samples.

C3 to C8: Describe the exposure measurement(s) used to produce this result.

C3. What is the exposure being measured and how was it measured or assessed?	Baseline circulating serum Lp(a) concentration measured from prediagnostic serum samples. Samples were sent to Scandinavian Health Ltd. laboratories and measured using the Roche Cobas 6000 analytical system. Lp(a) was analysed categorically in quartiles - highest quartile versus lowest quartile.
C4. Was exposure analysed as a quantitative (rather than a categorical) variable?	N
C5. Did repeated measurements of exposure over time (for each participant) contribute to the analysis that produced this result?	N
C6. **If Y/PY to C5**, was a single estimate of each participant's exposure level derived from the repeated measurements of exposure over time?	NA
C7. **If N/PN to C6**, was the analysis based on splitting participants' follow up time according to exposure status and/or magnitude?	NA
C8. **If Y/PY to C7**, were changes in exposure status and/or magnitude likely to be related to factors that are predictive of the outcome?	NA
C9. **If N/PN to C7**, how were repeat measurements used?	NA - no repeated exposure measurements contributed to this result.

Y = Yes; PY = Probably yes; PN = Probably no; N = No; NA = Not applicable

C10. Specify the relationship analysed to produce this result. For example, this may be a quadratic relationship of cumulative exposure with the log odds of the outcome, or a risk ratio for the outcome comparing exposed with unexposed individuals.

Prentice-weighted Cox proportional hazards model for estimating the hazard ratio for incident prostate cancer comparing men in the highest quartile of baseline Lp(a) concentration with men in the lowest quartile, using age as the time scale. The assessed result is the multivariable-adjusted Q4 vs Q1 HR 1.43 (95% CI 1.02 to 2.03).

D: Specify the causal effect of exposure being estimated by this result

D1. Specify the population of interest	Describe eligible participants (to whom the causal effect applies). These may be different from the study participants on whom the result was based (specified in C2). Such differences may give rise to selection biases.	Adult men free of prostate cancer at baseline in a general-population prospective cohort setting. Most directly applicable to middle-aged men similar to the EPIC-Heidelberg source population. The wider systematic review population is adult men in whom circulating Lp(a) may influence prostate cancer risk or adverse prostate cancer features.
Specification of the exposure metric of interest		
D2. Specify the exposure	This is the factor whose causal effect on the outcome of interest is the subject of the study result being assessed. It may be thought of as the 'true' exposure of interest. It is distinct from the method with which exposure was measured.	Higher circulating Lp(a) concentration.
D3. Specify the exposure window	The exposure window of interest is the exposure period for which the result being assessed estimates the effect of exposure on the outcome. Specification of the exposure window is judged by the ROBINS-E user, who should aim to define a window that is both meaningful in answering the review question and broadly in line with when the study measured exposure. Specification should include both the time of onset and period of exposure. For example, it may be lifetime exposure (from birth or from conception), during ages 50-55, the period from first employment in a particular occupation, time from birth to age 10, or during pregnancy.<br><br>The specified exposure window is used to determine whether exposure data adequately reflect exposure during the window. Exposure before the start of the exposure window is addressed during the assessment of risk of bias due to confounding	Prediagnostic circulating Lp(a) level at baseline representing usual long-term Lp(a) concentration before the occurrence of prostate cancer. The study measured exposure once at blood draw before diagnosis, because Lp(a) is largely genetically determined and relatively stable.

D4. Specify how exposure over time should be summarized	This may, for example, be ever/never exposed, cumulative exposure, average exposure, or peak exposure during the exposure period, for each participant. Alternatively, there may be only a single exposure event, or the exposure may be time invariant (such as a genetic variant or family history).	Usual circulating Lp(a) summarized by the baseline serum concentration and categorized into quartiles. The causal contrast of interest for this result is high versus low Lp(a) - highest quartile versus lowest quartile.

E. Evaluation of confounding factors

Complete a row for each important confounding factor listed in advance (subsection (i)). In addition, consider any further confounding factors that are either relevant to the setting of this particular study or which the study authors identified as potentially important (subsection (ii)).

"Important" confounding factors are those for which, in the context of this study, adjustment is expected to lead to an important change in the estimated effect of the exposure.

(i) Important confounding factors listed in advance

Confounding factor	Measured variable(s) for this factor, if any	Was this variable (or were these variables) controlled for in the analysis? (Y / N)	If this confounding factor was controlled for, was it measured validly and reliably by this variable (or these variables)?* (NA / Y / PY / PN / N / NI)	If this confounding factor was not controlled for, is there evidence that controlling for it was unnecessary?** (NA / Y / PY / PN / N)	Is failure to adjust for this confounding factor expected to bias the effect estimate towards benefit or harm of (higher) exposure?*** (Benefit of (higher) exposure / Harm of (higher) exposure / Insufficient information available)	Comments
Age	Age at recruitment, age used as underlying time scale in Cox model	Y	Y	NA	Insufficient information available	Strong prostate cancer risk factor.
Ethnicity	Not reported	N	NA	PN	Insufficient information available	Important for Lp(a) distribution and prostate cancer risk. Source population was a German cohort, which may reduce but does not eliminate this concern.
Family history	Not reported or adjusted	N	NA	N	Insufficient information available	Important prostate cancer risk factor, no

						evidence in the paper that adjustment was unnecessary.
BMI	BMI and waist circumference measured at baseline	Y	PY	NA	Insufficient information available	Anthropometry was measured by trained staff at recruitment. Residual measurement error likely limited.
Smoking	Smoking status and detailed smoking history variables	Y	PY	NA	Insufficient information available	Controlled using questionnaires, self-report may have some error but likely acceptable.
Alcohol intake and diet	Lifetime alcohol consumption, red meat intake, fibre intake	Y	PY	NA	Insufficient information available	Dietary variables were collected at baseline, some measurement error is likely but not expected to be substantial.
Physical activity	Physical activity category at baseline	Y	PY	NA	Insufficient information available	Controlled, however self reported physical activity can be imprecise.
Socioeconomic status / healthcare access	School level	Y	WN	NA	Insufficient information available	Education is a partial proxy and does not fully reflect healthcare access or PSA testing behavior.

Diabetes/ metabolic health	Self reported diabetes, hypertension also included	Y	PY	NA	Insufficient information available	Controlled in the multivariable model, self-report may have limited error
Lipid-lowering medication	Self reported use of lipid-lowering drugs at baseline	Y	PY	NA	Insufficient information available	Controlled in the main model and excluded in sensitivity analysis. Baseline use only.
PSA screening intensity	No direct measure of PSA testing or other screening behavior	N	NA	N	Risk of (higher) exposure	Potentially important confounder related with detection bias. If men with higher Lp(a) or CVD risk had more healthcare contact and PSA testing, prostate cancer incidence could be higher among those with higher Lp(a).

(ii) Additional confounding factors relevant to the setting of this particular study, or identified by study authors and considered to be important, or which were identified since the protocol was written						
Confounding factor	Measured variable(s) for this factor, if any	Was this variable (or were these variables) controlled for in the analysis? (Y / N)	If this confounding factor was controlled for, was it measured validly and reliably by this variable (or these variables)?* (NA / Y / PY / PN / N / NI)	If this confounding factor was not controlled for, is there evidence that controlling for it was unnecessary?** (NA / Y / PY / PN / N)	Is failure to adjust for this confounding factor expected to bias the effect estimate towards benefit or harm of (higher) exposure?*** (Benefit of (higher) exposure / Harm of (higher) exposure / Insufficient information available)	Comments

Liver or kidney insufficiency	Self reported chronic liver or kidney insufficiency excluded in sensitivity analyses	N (primary); sensitivity exclusions performed	PY	PY	Insufficient information available	These conditions can affect lipid biomarkers. Excluding prevalent liver or kidney insufficiency reportedly had negligible effects on estimates, reducing concern.
Baseline hypertension or healthcare contact due to CVD	Self reported hypertension, lipid-lowering drug use	Y	PY	NA	Risk of (higher) exposure	Partly controlled. Residual confounding due to healthcare contact may remain if cardiovascular-risk monitoring increased PSA testing.
Sex hormones/ androgen-related factors	Not measured	N	NA	NI	Insufficient information available	Potentially relevant to prostate cancer patophysiology and possibly lipid parameters, but no direct study data were available.

* “Validity” refers to whether the confounding variable or variables accurately measure the confounding factor, while “reliability” refers to the precision of the measurement (more measurement error means less reliability).

** In the context of a particular study, variables need not be included in the analysis: (a) if they are measured validly and reliably and are not associated with the outcome, conditional on exposure (noting that lack of a statistically significant association is not evidence of a lack of association); (b) if they are measured validly and reliably and are not associated with exposure; (c) if they are measured validly and reliably and adjustment makes no or minimal difference to the estimated effect of the primary parameter; (d) because the confounder was addressed in the study design, for example by restricting to individuals with the same value of the confounder; (e) because a negative control demonstrates that there was unlikely to have been confounding due to this variable or that uncontrolled confounding was likely to be minimal; or (f) because external evidence suggests that controlling for the variable is not necessary in the context of the study being assessed..

For each study: risk of bias assessment

Domain 1: Risk of bias due to confounding

Domain 1, Variant (a): ***If N/PN to C5 or Y/PY to C6 or N/PN to C7*** *(only baseline confounding needs to be addressed)*

Signalling questions	Response options	Comments
1.1 Did the authors control for all the important confounding factors for which this was necessary?	WN	The model controlled for many important factors (age, anthropometry, lifestyle, SES, diabetes, hypertension, lipid-lowering drugs), but not ancestry, family history, PSA screening intensity. Uncontrolled confounding is possible and likely most relevant for PSA screening and bias due to healthcare contact.
1.2 **If Y/PY/WN to 1.1**: Were confounding factors that were controlled for (and for which control was necessary) measured validly and reliably by the variables available in this study?	WN	Most controlled variables were measured at baseline using questionnaire or anthropometry. Anthropometry was likely valid but self reported lifestyle, medical history, and medication variables may have measurement error. Education only partly measures healthcare access.
1.3 **If Y/PY/WN to 1.1**: Did the authors control for any variables after the start of the exposure period being studied that could have been affected by the exposure?	N	Covariates used for adjustment were baseline characteristics measured at recruitment, not variables measured after the start of follow-up that were clearly affected by Lp(a).
1.4 Did the use of negative controls, or other considerations, suggest serious uncontrolled confounding?	PN	No negative-control analysis was reported. The authors performed sensitivity analyses for reverse causation and exclusions for lipid-lowering drugs/ liver or kidney disease, which did not indicate serious uncontrolled confounding for this result.
Risk of bias (due to confounding) in the estimated effect of exposure on the outcome	Some concerns	Main concern is residual confounding from unmeasured PSA screening, healthcare contact, ancestry, and family history. Extensive adjustment and prospective design reduce but do not remove this concern.
What is the predicted direction of bias due to confounding?	Insufficient information available	Bias could be towards apparent harm of higher Lp(a) if men with higher Lp(a) and CVD risk had more healthcare contact and PSA testing, but this mechanism was not directly measured.
Is the risk of bias (due to confounding) sufficiently high, in the context of its likely direction and the magnitude of the estimated exposure effect, to threaten conclusions about whether the exposure has an important effect on the outcome?	Cannot tell	The estimated association is modest (HR 1.43), so residual confounding bias could plausibly affect interpretation, but the available study data do not show that such bias was substantial.

Y = Yes; PY = Probably yes; PN = Probably no; N = No; SY = Strong yes; WY = Weak yes; SN = Strong no; WN = Weak no; NA = Not applicable; NI = No information

*Domain 1, variant (b): **If Y/PY to C7 and Y/PY to C8** (the analysis was based on splitting participants' follow up time according to exposure status and/or magnitude and changes in exposure status and/or magnitude likely to be related to factors that are predictive of the outcome, so both baseline and time-varying confounding need to be addressed)*

**Signalling questions**	**Response options**	**Comments**
1.1 Did the authors use an analysis method that was appropriate to control for time-varying as well as baseline confounding?	NA	Not applicable - exposure was measured at a single baseline time point and the analysis did not split follow-up by time-varying exposure status.
1.2 **If Y/PY to 1.1**: Did the authors control for all the important baseline and time-varying confounding factors for which this was necessary?	NA	
1.3 **If Y/PY/WN to 1.2**: Were confounding factors that were controlled for (and for which control was necessary) measured validly and reliably by the variables available in this study?	NA	
1.4 **If N/PN/NI to 1.1**: Did the authors control for time-varying factors or other variables measured after the start of the exposure window being studied?	NA	
1.5 Did the use of negative controls, or other considerations, suggest uncontrolled confounding?	NA	
Risk of bias (due to confounding) in the estimated effect of exposure on the outcome	NA	
What is the predicted direction of bias due to confounding?	NA	
Is the risk of bias (due to confounding) sufficiently high, in the context of its likely direction and the magnitude of the estimated exposure effect, to threaten conclusions about whether the exposure has an important effect on the outcome?	NA	

Y = Yes; PY = Probably yes; PN = Probably no; N = No; SY = Strong yes; WY = Weak yes; SN = Strong no; WN = Weak no; NA = Not applicable; NI = No information

Domain 2: Risk of bias arising from measurement of the exposure

*Domain 2, Variant (a): **If N/PN to C5** (exposure was measured at a single point in time)*

Signalling questions	Response options	Comments
Mismeasurement or misclassification of the exposure.		
2.1 Does the measured exposure well-characterize the exposure metric specified to be of interest in this study? [*This was specified in the answers to D2, D3 and D4*]	PY	Baseline serum Lp(a) measured before diagnosis reasonably characterizes usual Lp(a), particularly because Lp(a) is relatively stable. However, lifetime exposure and long-term fluctuation were not directly measured.
2.2 Was the exposure likely to be measured with error, or misclassified?	WY	Measurement used an automated clinical chemistry system, but the paper does not specify whether the assay was apo(a)-isoform insensitive. A small proportion (<10%) of analytes were not successfully measured.
Bias in the estimated effect of exposure arising from mismeasurement or misclassification of the exposure		
2.3 If SY/WY to 2.2: Could mismeasurement or misclassification of exposure have been differential (i.e. related to the outcome or risk of the outcome)?	PN	Exposure was measured in stored baseline samples, before prostate cancer diagnosis, hence differential misclassification by future outcome is unlikely.
2.4 If SY/WY to 2.2 and N/PN/WY to 2.3: Is non-differential measurement error likely to bias the estimated effect of exposure on outcome?	WY	Non-differential measurement error or quartile misclassification could weaken this association, though Lp(a) stability makes large error less likely.
Risk of bias (arising from measurement of exposure) in the estimated effect of exposure on the outcome	Some concerns	The assay and prospective measurement are acceptable, but single measurement and lack of information on insensitivity of apo(a) isoform justify some concerns.
What is the predicted direction of bias arising from measurement of exposure?	Towards null	Likely non-differential measurement error would most plausibly weaken a true association.
Is the risk of bias (arising from measurement of exposure) sufficiently high, in the context of its likely direction and the magnitude of the estimated exposure effect, to threaten conclusions about whether the exposure has an important effect on the outcome?	No	Any exposure measurement bias is unlikely to be large enough by itself to reverse conclusions.

Y = Yes; PY = Probably yes; PN = Probably no; N = No; SY = Strong yes; WY = Weak yes; SN = Strong no; WN = Weak no; NA = Not applicable; NI = No information

*Domain 2, Variant (b): **If Y/PY to C5 and Y/PY to C6** (each individual's exposure level was estimated from measurements made at multiple time points)*

**Signalling questions**	**Response options**	**Comments**
2.1 Does the measured exposure (derived from measurements at multiple time points) well-characterize the exposure metric specified to be of interest in this study? [*This was specified in the answers to D2, D3 and D4*]	NA	Not applicable: the assessed result used a single baseline Lp(a) measurement, not repeated exposure measurements over time.
2.2 Was there error in measurement, or misclassification, of the exposure, at each single time point?	NA	
2.3 If SY/WY to 2.2: Could mismeasurement or misclassification of exposure have been differential (i.e. related to the outcome or risk of the outcome)?	NA	
2.4 If SY/WY to 2.2 and N/PN/WY to 2.3: Is the nature of the (non-differential) measurement error likely to bias the estimated effect of exposure on outcome?	NA	
Risk of bias (arising from measurement of exposure) in the estimated effect of exposure on the outcome	NA	
What is the predicted direction of bias arising from measurement of exposure?	NA	
Is the risk of bias (arising from measurement of exposure) sufficiently high, in the context of its likely direction and the magnitude of the estimated exposure effect, to threaten conclusions about whether the exposure has an important effect on the outcome?	NA	

Y = Yes; PY = Probably yes; SN = Strong no; WN = Weak no; NA = Not applicable; NI = No information

*Domain 2, Variant (c): **If Y/PY to C5, N/PN to C6 and Y/PY to C7** (the analysis was based on splitting participants' follow up time according to exposure status and/or magnitude):*

Signalling questions	Response options	Comments
2.1 Does the measured exposure (including changes over time) well-characterize the exposure metric specified to be of interest in this study? [*This was specified in the answers to D2, D3 and D4*]	NA	Not applicable: the assessed result used a single baseline Lp(a) measurement, not repeated exposure measurements over time.
2.2 Was there error in measurement, or misclassification, of the exposure, at each single time point?	NA	
2.3 If SY/WY to 2.2: Could mismeasurement or misclassification of exposure have been differential (i.e. related to the outcome or risk of the outcome)?	NA	
2.4 If SY/WY to 2.2 and N/PN/WY to 2.3: Is the nature of the (non-differential) measurement error likely to bias the estimated effect of exposure on outcome?	NA	
Risk of bias (arising from measurement of exposure) in the estimated effect of exposure on the outcome	NA	
What is the predicted direction of bias<br><br>arising from measurement of exposure?	NA	
Is the risk of bias (arising from measurement of exposure) sufficiently high, in the context of its likely direction and the magnitude of the estimated exposure effect, to threaten conclusions about whether the exposure has an important effect on the outcome?	NA	

Y = Yes; PY = Probably yes; SN = Strong no; WN = Weak no; NA = Not applicable; NI = No information

Domain 3: Risk of bias in selection of participants into the study (or into the analysis)

Signalling questions	Response options	Comments
3.1 Did follow-up begin at (or close to) the start of the exposure window for most participants? [*The exposure window is specified in D3*]	Y	Follow-up began at baseline when the blood was drawn, which is the start of the exposure window for the measured exposure.
3.2 **If N/PN to 3.1**: Is the effect of exposure likely to be constant over the period of follow up analysed?	NA	Not applicable because follow-up began at or close to the start of the exposure.
3.3 Was selection of participants into the study (or into the analysis) based on participant characteristics observed after the start of the exposure window being studied? [*The exposure window is specified in D3*]	PY	The case-cohort analysis included all verified incident prostate cancer cases identified after baseline plus a random subcohort. Inclusion of cases which is dependent by outcome is therefore inherent to the case-cohort design.
3.4 **If Y/PY to 3.3**: Were these characteristics likely to be influenced by exposure or a cause of exposure?	Y	Incident prostate cancer is the outcome and could be influenced by the exposure under study.
3.5 **If Y/PY to 3.4**: Were these characteristics likely to be influenced by the outcome or a cause of the outcome?	Y	The characteristic used for case inclusion was the outcome itself.
3.6 **If N/PN to 3.2 or Y/PY to 3.5**: Is it likely that the analysis corrected for all of the potential selection biases identified in A and B above?	PY	The authors used the Prentice weighting scheme for case-cohort analyses, and the reference subcohort was randomly selected independently of outcome.
3.7 **If N/PN to 3.2 or Y/PY to 3.5**: Did sensitivity analyses demonstrate that the likely impact of the potential selection biases identified in A or B above was minimal?	NA	No separate sensitivity analysis for case-cohort sampling was necessary because the design was addressed analytically by Prentice weighting. Sensitivity analyses addressed reverse causation and exclusions.
Risk of bias (due to selection of participants into the study) in the estimated effect of exposure on the outcome	Low risk	The case-cohort design and random subcohort selection were handled with appropriate Prentice-weighted Cox modelling. Exclusion because of unavailable blood samples was a minor concern, but insufficient to change the domain-level judgement.
What is the predicted direction of bias<br><br>due to selection of participants into the study?	Insufficient information available	No clear direction of selection bias is evident.
Is the risk of bias (due to selection of participants into the study) sufficiently high, in the context of its likely direction and the magnitude of the estimated exposure effect, to	No	Selection bias is unlikely to be sufficiently large to threaten the result.

Signalling questions	Response options	Comments
threaten conclusions about whether the exposure has an important effect on the outcome?		

Y = Yes; PY = Probably yes; PN = Probably no; N = No; SN = Strong no; WN = Weak no; NA = Not applicable; NI = No information

Domain 4: Risk of bias due to post-exposure interventions

Signalling questions	Response options	Comments
4.1 Were there post-exposure interventions that were influenced by prior exposure during the follow-up period?	PN	Lp(a) was measured for research at baseline and is unlikely to have directly influenced subsequent interventions or clinical management. Post-baseline lipid treatment or PSA screening could occur, but there is no evidence these were driven by measured Lp(a).
4.2 **If Y/PY to 4.1**: Is it likely that the analysis corrected for the effect of post-exposure interventions that were influenced by prior exposure?	NA	Not applicable because exposure-influenced post-exposure interventions were not identified.
Risk of bias (due post-exposure interventions) in the estimated effect of exposure on the outcome	Low risk	No clear post-exposure intervention pathway from measured Lp(a) to prostate cancer ascertainment was identified.
What is the predicted direction of bias due to confounding?	Insufficient information available	No specific direction of bias is evident.
Is the risk of bias (due post-exposure interventions) sufficiently high, in the context of its likely direction and the magnitude of the estimated exposure effect, to threaten conclusions about whether the exposure has an important effect on the outcome?	No	This domain is unlikely to threaten conclusions.

Y = Yes; PY = Probably yes; PN = Probably no; N = No; NA = Not applicable; NI = No information

Domain 5: Risk of bias due to missing data

Signalling questions	Response options	Comments
5.1 Were complete data on exposure status available for all, or nearly all, participants?	PY	Exposure data were available for nearly all included participants, but the authors state that a small proportion (<10%) of analytes were not successfully measured and sample size could vary by model.
5.2 Were complete data on the outcome available for all, or nearly all, participants?	PY	Outcome follow-up used active follow-up plus registry linkage. Complete loss-to-follow-up information for the prostate cancer analysis was not detailed, but outcome verification appears to be complete.
5.3 Were complete data on confounding variables available for all, or nearly all, participants?	NI	The paper does not clearly report completeness for all adjustment variables in the prostate cancer model.
5.4 **If N/PN/NI to 5.1, 5.2 or 5.3**: Is the result based on a complete case analysis?	PY	No imputation was described. Varying numbers by statistical model imply complete case analysis for missing exposure data.
5.5 **If Y/PY/NI**: Was exclusion from the analysis because of missing data (in exposure, confounders or the outcome) likely to be related to the true value of the outcome?	PN	There is no clear evidence that exclusion due to missing Lp(a) or covariates was related to true prostate cancer outcome, but this cannot be fully verified.
5.6 **If N/PN to 5.5**: Were all or most predictors of missingness (in exposure, confounders or the outcome) included in the analysis model?	WY	The adjusted model included many predictors that could relate to missingness and outcome, but missingness predictors were not explicitly modelled.
5.7 **If N/PN to 5.4**: Was the analysis based on imputing missing values?	NA	Not applicable because the analysis appears to be complete case rather than imputed.
5.8 **If Y/PY to 5.7**: Was imputation performed appropriately?	NA	Not applicable. No imputation was reported.
5.9 **If N/PN to 5.7**: Was an appropriate alternative method used to correct for bias due to missing data?	NA	Not applicable. The result appears to be based on complete case analysis, and no alternative correction for missing data was reported.
5.10 **If PN/N/NI to 5.1, 5.2 or 5.3**: Is there evidence that the result was not biased by missing data?	PN	No sensitivity analysis for missing data was reported for the prostate cancer result.
Risk of bias (due to missing data) in the estimated effect of exposure on the outcome	Some concerns	Small exposure missingness and unclear covariate completeness lead to some concerns, but there is no evidence of substantial bias due to missing data.

Signalling questions	Response options	Comments
What is the predicted direction of bias due to missing data?	Insufficient information available	The direction of any bias due to missing data cannot be predicted.
Is the risk of bias (due to missing data) sufficiently high, in the context of its likely direction and the magnitude of the estimated exposure effect, to threaten conclusions about whether the exposure has an important effect on the outcome?	No	Available information does not suggest bias due to missing data which is large enough to threaten conclusions.

Y = Yes; PY = Probably yes; PN = Probably no; N = No; SY = Strong yes; WY = Weak yes; NA = Not applicable; NI = No information

Domain 6: Risk of bias arising from measurement of the outcome

**Signalling questions**	**Response options**	**Comments**
6.1 Could measurement or ascertainment of the outcome have differed between exposure groups or levels of exposure?	PN	Incident prostate cancer ascertainment was through active follow-up plus pathology registry linkage and ICD-10 coding. This is unlikely to differ by baseline Lp(a) quartile.
6.2 Were outcome assessors aware of study participants' exposure history?	PN	Registry or pathology ascertainment and coding were unlikely to be informed by the participant's baseline Lp(a) level.
6.3 **If Y/PY/NI to 6.2**: Could assessment of the outcome have been influenced by knowledge of participants' exposure history?	NA	Not applicable because outcome assessors were probably not aware of exposure history.
Risk of bias (arising from measurement of outcomes) in the estimated effect of exposure on the outcome	Low risk	Outcome definition and verification appear appropriate and not dependent on exposure.
What is the predicted direction of bias arising from measurement of outcomes?	Insufficient information available	No important outcome measurement bias expected.
Is the risk of bias (arising from measurement of outcomes) sufficiently high, in the context of its likely direction and the magnitude of the estimated exposure effect, to threaten conclusions about whether the exposure has an important effect on the outcome?	No	Outcome measurement bias is not likely to threaten conclusions.

Y = Yes; PY = Probably yes; PN = Probably no; N = No; SY = Strong yes; WY = Weak yes; NA = Not applicable; NI = No information

Domain 7: Risk of bias in selection of the reported result

Signalling questions	Response options	Comments
7.1 Was the result reported in accordance with an available, pre-determined analysis plan?	NI	No publicly available prespecified analysis plan or protocol was reported in the article.
7.2 **If N/PN/NI to 7.1**: Is the reported effect estimate likely to be selected, based on desirability of the magnitude (or statistical significance) of the estimated effect of exposure on outcome, from multiple *exposure measurements* within the exposure domain?	PN	Although several lipid biomarkers were measured, the paper reports results across biomarkers and cancer sites rather than only the significant Lp(a)-prostate cancer association.
7.3 Is the reported effect estimate likely to be selected, based on desirability of the magnitude (or statistical significance) of the estimated effect of exposure on outcome, from multiple *outcome measurements* within the outcome domain?	PN	The outcome domain for this result is incident prostate cancer, defined using ICD coding. No alternative prostate cancer outcome measurement was evident for the same result.
7.4 Is the reported effect estimate likely to be selected, based on desirability of the magnitude (or statistical significance) of the estimated effect of exposure on outcome, from multiple *analyses* of the exposure-outcome relationship?	PY	The study examined multiple biomarkers, quartiles, continuous models, clinical cut-off points and several cancer and CVD endpoints. Without a prespecified plan, selection of the highlighted result or model cannot be excluded, even though results were reported broadly.
7.5 Is the reported effect estimate likely to be selected, based on the basis of desirability of the results (e.g. statistical significance), from different *subgroups*?	PN	The assessed prostate cancer estimate was not presented as an estimate selected by subgroup. It was the main site-specific prostate cancer result.
Risk of bias (due to selection of the reported result) in the estimated effect of exposure on the outcome	Some concerns	Lack of prespecified plan and multiple comparisons for exposure, outcome and model raise some concerns, but the broad reporting of results reduces concern for selective reporting.
What is the predicted direction of bias due to selection of the reported result?	Towards harm of (higher) exposure	If selective emphasis occurred, it would plausibly favor the statistically significant harmful association of high Lp(a) with prostate cancer.
Is the risk of bias (due to selection of the reported result) sufficiently high, in the context of its likely direction and the magnitude of the estimated exposure effect, to	Cannot tell	Concerns due to selection of the reported results concerns are not severe, but the modest effect estimate means interpretation should be cautious.

Signalling questions	Response options	Comments
threaten conclusions about whether the exposure has an important effect on the outcome?		

Y = Yes; PY = Probably yes; PN = Probably no; N = No; NA = Not applicable; NI = No information

Overall risk of bias

	Response options	Comments
Overall risk of bias	Some concerns	Overall judgement: Some concerns. The study has strong features for ROBINS-E assessment: prospective case-cohort design, prediagnostic blood collection, registry and pathology outcome ascertainment, Prentice-weighted Cox modelling, broad adjustment, and sensitivity analyses for reverse causation and selected exclusions. Main concerns are residual confounding and detection bias from unmeasured PSA screening and healthcare access, ancestry and family history, single Lp(a) measurement with limited details of the assay, incomplete reporting of missing covariate data and absence of prespecified analysis plan despite multiple biomarker and outcome comparisons.
What is the predicted direction of bias?	Insufficient information available	Most potential biases are probably non-differential or unpredictable. Residual PSA-screening or confounding due to healthcare contact could bias toward apparent risk of higher Lp(a), but this was not directly measured.
Is the overall risk of bias sufficiently high, in the context of its likely direction and the magnitude of the estimated exposure effect, to threaten conclusions about whether the exposure has an important effect on the outcome?	Cannot tell	The risk of bias is not high enough to discard the result, but because the observed association is modest (HR 1.43, 95% CI 1.02 to 2.03), residual confounding or detection bias could affect conclusions about whether higher Lp(a) has an important causal effect on prostate cancer incidence.

This work is licensed under a Creative Commons Attribution-NonCommercial-NoDerivatives 4.0 International License.
