# Supplementary material for: Lipoprotein(a) and Prostate Cancer: A Systematic Review of Observational, Genetic and Mendelian Randomization Evidence
Source: Int J Mol Sci. 2026 Sep 17;27(18):8274. doi: 10.3390/ijms27188274 (PMC13607466; doi:10.3390/ijms27188274)
Supplement: Supplementary file 1 [file ijms-27-08274-s001.zip › Supplementary Material S4.pdf]

The Risk Of Bias In Non-randomized Studies – of Exposures (ROBINS-E) assessment tool
(for follow-up studies)

Template for completion

Version 24 March 2024

This work is licensed under a Creative Commons Attribution-NonCommercial-NoDerivatives 4.0 International License.

The ROBINS-E tool

At planning stage: list confounding factors and consider appropriateness criteria

P1. List the important confounding factors relevant to all or most studies on this topic. Specify whether these are particular to specific exposures-outcome combinations.

Age, ethnicity or genetic ancestry, family history and inherited susceptibility to prostate cancer, socioeconomic position, access to health care, PSA testing and screening behavior, BMI and adiposity, diabetes and metabolic health, smoking, alcohol intake, physical activity, diet, lipid lowering medication, cardiovascular disease and health care contact, liver disease, renal disease, inflammation, and endogenous hormone related factors. PSA testing and health care contact are important for incident prostate cancer because they may influence diagnosis. Ethnicity or genetic ancestry is particularly important because Lp(a) concentrations differ strongly by genetic background.

P2. Will the review use the ROBINS-E assessment of appropriateness (important aspects of “study sensitivity”)?

No

If Yes, complete sections Addressing appropriateness, Parts I and II in Appendix 1.

For each study result: preliminary considerations

A. Specify the result being assessed for risk of bias

A1. Specify the numerical result being assessed

Multivariable Cox proportional hazards estimate for baseline plasma Lp(a) quartiles and incident prostate cancer over 10 years among cancer free men. Main prostate cancer result from Table 5: Q2 compared with Q1 HR 1.51, 95% CI 0.93 to 2.44; Q3 compared with Q1 HR 1.24, 95% CI 0.76 to 2.05; Q4 compared with Q1 HR 0.84, 95% CI 0.49 to 1.45; P value 0.12. The assessment focuses on the Q4 compared with Q1 result and the overall quartile pattern for prostate cancer incidence.

B. Decide whether to proceed with a risk-of-bias assessment

	Response options	Comments
B1. Did the authors make any attempt to control for confounding?	Y	The authors used multivariable Cox proportional hazards models. The prostate cancer models were adjusted for centre, age group, smoking status, and alcohol consumption. Additional variables were considered elsewhere in the paper, but the site specific prostate model in Table 5 used the core adjustment set.
B2. **If N/PN to B1**: Is there sufficient potential for confounding that an unadjusted result should not be considered further?	NA	Not applicable because the authors made an attempt to control for confounding.
B3. Was the method of measuring exposure inappropriate?	N	Baseline fasting plasma Lp(a) was measured using a bi site immunoenzymatic assay in a central laboratory after standardized blood collection and processing. This is an appropriate exposure measurement method, although the paper gives limited information about isoform sensitivity.
B4. Was the method of measuring the outcome inappropriate?	N	Incident prostate cancer was identified during annual follow up, with medical inquiry for suspected cancers, systematic pursuit of histological confirmation, and cancer site coding using ICD 9 and FRANCIM classification. This is an appropriate outcome measurement approach.

If the answer to any of B2, B3 or B4 is 'Yes' or 'Probably yes', the result should be considered to be at very high risk of bias and no further assessment is required. Otherwise, proceed to section C.

C. Specify the analysis in the current study for which results are being assessed for risk of bias

C1. Specify the outcome to which this result relates.

Incident prostate cancer during 10 years of follow up.

C2. Specify the participant group on which this result was based.

Men aged 50 to 59 years from the Strasbourg and Lille centres of the PRIME cohort, living in the Bas Rhin county or the Urban Community of Lille, free from former or current cancer at baseline, with available baseline Lp(a) and covariate data. The analysis included 5196 men after excluding 41 men with missing Lp(a).

C3 to C8: Describe the exposure measurement(s) used to produce this result.

C3. What is the exposure being measured and how was it measured or assessed?	Plasma Lp(a) concentration measured once at baseline from fasting blood samples. Blood was collected according to a standardized protocol, plasma was separated within 15 minutes at 4 degrees C, stored cold, sent weekly to the central laboratory, and Lp(a) was measured by a bi site immunoenzymatic assay with a lowest detection limit of 10 mg per L. Lp(a) was analysed in quartiles in the prostate cancer model.
C4. Was exposure analysed as a quantitative (rather than a categorical) variable?	N
C5. Did repeated measurements of exposure over time (for each participant) contribute to the analysis that produced this result?	N
C6. **If Y/PY to C5**, was a single estimate of each participant’s exposure level derived from the repeated measurements of exposure over time?	NA
C7. **If N/PN to C6**, was the analysis based on splitting participants’ follow up time according to exposure status and/or magnitude?	NA
C8. **If Y/PY to C7**, were changes in exposure status and/or magnitude likely to be related to factors that are predictive of the outcome?	NA

C9. **If N/PN to C7**, how were repeat measurements used?

Not applicable. Lp(a) was measured once at baseline and no repeated measurements contributed to the prostate cancer analysis.

Y = Yes; PY = Probably yes; PN = Probably no; N = No; NA = Not applicable

C10. Specify the relationship analysed to produce this result. For example, this may be a quadratic relationship of cumulative exposure with the log odds of the outcome, or a risk ratio for the outcome comparing exposed with unexposed individuals.

Cox proportional hazards model estimating incident prostate cancer by baseline Lp(a) quartiles. The reference group was Q1, less than 10 mg per L. The highest exposure category was Q4, at least 170 mg per L. The main assessed contrast is Q4 compared with Q1, adjusted for centre, age group, smoking status, and alcohol consumption.

D: Specify the causal effect of exposure being estimated by this result

D1. Specify the population of interest	Describe eligible participants (to whom the causal effect applies). These may be different from the study participants on whom the result was based (specified in C2). Such differences may give rise to selection biases.	Cancer free middle aged men in a general population setting similar to the French PRIME centres. The causal contrast of interest applies to men without prostate cancer at baseline, before subsequent diagnosis during follow up.
Specification of the exposure metric of interest		
D2. Specify the exposure	This is the factor whose causal effect on the outcome of interest is the subject of the study result being assessed. It may be thought of as the 'true' exposure of interest. It is distinct from the method with which exposure was measured.	Higher usual circulating Lp(a) concentration, considered as a biological exposure before prostate cancer diagnosis.
D3. Specify the exposure window	The exposure window of interest is the exposure period for which the result being assessed estimates the effect of exposure on the outcome. Specification of the exposure window is judged by the ROBINS-E user, who should aim to define a window that is both meaningful in answering the review question and broadly in line with when the study measured exposure. Specification should include both the time of onset and period of exposure. For example, it may be lifetime exposure (from birth or from conception), during ages 50-55, the period from first employment in a particular occupation, time from birth to age 10, or during pregnancy.<br><br>The specified exposure window is used to determine whether exposure data adequately reflect exposure during the window. Exposure before the start of the exposure window is addressed during the assessment of risk of bias due to confounding	Usual circulating Lp(a) before prostate cancer diagnosis, represented by the fasting baseline blood sample collected at study entry. Since Lp(a) is largely genetically determined and relatively stable, one baseline measurement is intended to approximate usual exposure during the pre diagnosis period.
D4. Specify how exposure over time	This may, for example, be ever/never exposed, cumulative exposure, average exposure, or peak exposure during the exposure period, for each participant. Alternatively, there may be only a	Lp(a) was summarized as baseline plasma concentration grouped into quartiles. The main comparison assessed here is highest quartile against lowest quartile, with attention to the full quartile pattern.

should be summarized	single exposure event, or the exposure may be time invariant (such as a genetic variant or family history).	

E. Evaluation of confounding factors

Complete a row for each important confounding factor listed in advance (subsection (i)). In addition, consider any further confounding factors that are either relevant to the setting of this particular study or which the study authors identified as potentially important (subsection (ii)).

"Important" confounding factors are those for which, in the context of this study, adjustment is expected to lead to an important change in the estimated effect of the exposure.

(i) Important confounding factors listed in advance						
Confounding factor	Measured variable(s) for this factor, if any	Was this variable (or were these variables) controlled for in the analysis?<br>(Y / N)	If this confounding factor was controlled for, was it measured validly and reliably by this variable (or these variables)?*<br>(NA / Y / PY / PN / N / NI)	If this confounding factor was not controlled for, is there evidence that controlling for it was unnecessary?**<br>(NA / Y / PY / PN / N)	Is failure to adjust for this confounding factor expected to bias the effect estimate towards benefit or harm of (higher) exposure?***<br>(Benefit of (higher) exposure / Harm of (higher) exposure / Insufficient information available)	Comments
Age	Age group below 55 years and at least 55 years	Y	PY	NA	Insufficient information	Age is a strong determinant of prostate cancer incidence. The study restricted age to 50 to 59 years and adjusted for broad age group. Some residual confounding by age may remain within categories.
Ethnicity or genetic ancestry	Not measured	N	NA	PY	Insufficient information	The source population was from two French centres. The paper does not report ethnicity or ancestry. If the cohort was relatively homogeneous, failure to adjust may have limited impact, but this cannot be confirmed.

Family history and inherited susceptibility	Not measured	N	NA	N	Insufficient information	Family history is an important prostate cancer risk factor. The study did not measure or adjust for it, and no evidence was provided that adjustment was unnecessary.
Socioeconomic position and health care access	Educational level, economic activity, material conditions	N	NA	PN	Insufficient information	These variables were described and included in broader adjustment considerations, but the prostate cancer model in Table 5 was adjusted only for centre, age, smoking, and alcohol. Socioeconomic position may influence PSA testing and diagnosis.
PSA testing and screening behaviour	Not measured	N	NA	N	Insufficient information	This is a key source of detection bias for prostate cancer incidence. Screening behaviour may be related to socioeconomic position, medical contact, and cardiovascular risk monitoring, but it was not measured.

BMI and adiposity	Measured BMI at baseline	N	NA	PN	Insufficient information	BMI differed across Lp(a) quartiles and interacted with Lp(a) for all cancer incidence. The prostate cancer model in Table 5 did not include BMI. This raises some concern.
Smoking	Smoking status	Y	PY	NA	Insufficient information	Smoking was controlled using categories of never, former, and current smoking. Broad self report categories may contain some error.
Alcohol intake	Alcohol consumption categories	Y	PY	NA	Insufficient information	Alcohol intake was controlled using categories of total abstainer, below 30 g per day, and at least 30 g per day. Self report error is possible.
Physical activity	Physical activity categories	N	NA	PN	Insufficient information	Physical activity was measured but not included in the Table 5 prostate cancer model. It is probably a weaker confounder for the Lp(a) and prostate cancer question than PSA testing and family history.

Diabetes and metabolic health	No direct diabetes adjustment reported for the prostate cancer model	N	NA	PN	Insufficient information	Diabetes may be related to prostate cancer detection and lipid metabolism. The study recorded several cardiovascular risk variables, but diabetes was not part of the reported prostate model.
Lipid lowering medication	Use of lipid lowering drugs at baseline	N	NA	PY	Insufficient information	The authors examined cancer analyses after excluding 598 men using lipid lowering drugs and found no material change for overall cancer. This reduces concern, but a prostate specific sensitivity analysis was not presented.
Hormone related factors	Not measured	N	NA	N	Insufficient information	The authors noted that endogenous androgens may confound the Lp(a) and prostate cancer relation, but hormone levels were not measured.

(ii) Additional confounding factors relevant to the setting of this particular study, or identified by study authors and considered to be important, or which were identified since the protocol was written

Confounding factor	Measured variable(s) for this factor, if any	Was this variable (or were these variables) controlled for in the analysis?<br>(Y / N)	If this confounding factor was controlled for, was it measured validly and reliably by this variable (or these variables)?*<br>(NA / Y / PY / PN / N / NI)	If this confounding factor was not controlled for, is there evidence that controlling for it was unnecessary?**<br>(NA / Y / PY / PN / N)	Is failure to adjust for this confounding factor expected to bias the effect estimate towards benefit or harm of (higher) exposure?***<br>(Benefit of (higher) exposure / Harm of (higher) exposure / Insufficient information available)	Comments
Centre and local diagnostic practice	Strasbourg and Lille centre indicator	Y	PY	NA	Insufficient information	Centre was controlled. It may partly capture regional differences in recruitment, laboratory handling, medical care, and cancer diagnosis.
History of dyslipidaemia	Self reported history of dyslipidaemia	N	NA	PY	Insufficient information	History of dyslipidaemia differed modestly across Lp(a) quartiles and may reflect health care contact. It was not included in the prostate cancer model, although broader adjustment did not materially alter all cancer estimates.

Liver disease or liver function	Not measured	N	NA	N	Insufficient information	The authors noted that liver disease can affect Lp(a). They excluded liver cancers in sensitivity analysis for all cancer, but liver function data were unavailable.
Competing cardiovascular events and mortality	Deaths and follow up status recorded	N	NA	PN	Insufficient information	Higher Lp(a) is related to cardiovascular risk. Competing events could influence the chance of later prostate cancer diagnosis, but no competing risk analysis was reported.

* "Validity" refers to whether the confounding variable or variables accurately measure the confounding factor, while "reliability" refers to the precision of the measurement (more measurement error means less reliability).

** In the context of a particular study, variables need not be included in the analysis: (a) if they are measured validly and reliably and are not associated with the outcome, conditional on exposure (noting that lack of a statistically significant association is not evidence of a lack of association); (b) if they are measured validly and reliably and are not associated with exposure; (c) if they are measured validly and reliably and adjustment makes no or minimal difference to the estimated effect of the primary parameter; (d) because the confounder was addressed in the study design, for example by restricting to individuals with the same value of the confounder; (e) because a negative control demonstrates that there was unlikely to have been confounding due to this variable or that uncontrolled confounding was likely to be minimal; or (f) because external evidence suggests that controlling for the variable is not necessary in the context of the study being assessed..

For each study: risk of bias assessment

Domain 1: Risk of bias due to confounding

<u>*Domain 1, Variant (a): **If N/PN to C5 or Y/PY to C6 or N/PN to C7** (only baseline confounding needs to be addressed)*</u>

Signalling questions	Response options	Comments
1.1 Did the authors control for all the important confounding factors for which this was necessary?	WN	The authors controlled for age, centre, smoking, and alcohol consumption. Important factors for prostate cancer diagnosis were not controlled, including PSA testing, family history, socioeconomic position in the prostate model, BMI, health care contact, and hormone related factors. Uncontrolled confounding is possible, but its size is uncertain.
1.2 **If <u>Y/PY/WN</u> to 1.1**: Were confounding factors that were controlled for (and for which control was necessary) measured validly and reliably by the variables available in this study?	WN	Controlled confounders were measured with reasonable but broad categories. Age and centre were likely reliable. Smoking and alcohol were self reported and may have some error, but the error is unlikely to be the dominant limitation.
1.3 **If <u>Y/PY/WN</u> to 1.1**: Did the authors control for any variables after the start of the exposure period being studied that could have been affected by the exposure?	N	The reported prostate cancer model did not control for variables measured after the start of the exposure period that could clearly have been affected by Lp(a).
1.4 Did the use of negative controls, or other considerations, suggest serious uncontrolled confounding?	PN	No negative control was used. However, the prospective design, restriction to cancer free men, and sensitivity analyses excluding early cases reduce but do not remove concerns about confounding and reverse causation.
Risk of bias (due to confounding) in the estimated effect of exposure on the outcome	Some concerns	Confounding is a meaningful concern because PSA testing, family history, health care contact, BMI, and hormone related factors were not fully handled. The study still controlled for several basic factors and measured Lp(a) before diagnosis.
What is the predicted direction of bias due to confounding?	Insufficient information	Residual confounding could plausibly operate in either direction. Detection through PSA testing is especially difficult to predict.
Is the risk of bias (due to confounding) sufficiently high, in the context of its likely direction and the magnitude of the estimated exposure effect, to threaten conclusions about whether the exposure has an important effect on the outcome?	Cannot tell	Residual confounding could affect the estimate, but the prostate result was imprecise and not statistically significant. It is not possible to tell whether confounding fully explains the observed pattern.

Y = Yes; PY = Probably yes; PN = Probably no; N = No; SY = Strong yes; WY = Weak yes; SN = Strong no; WN = Weak no; NA = Not applicable; NI = No information

*Domain 1, variant (b): **If Y/PY to C7 and Y/PY to C8** (the analysis was based on splitting participants' follow up time according to exposure status and/or magnitude and changes in exposure status and/or magnitude likely to be related to factors that are predictive of the outcome, so both baseline and time-varying confounding need to be addressed)*

**Signalling questions**	**Response options**	**Comments**
1.1 Did the authors use an analysis method that was appropriate to control for time-varying as well as baseline confounding?	NA	Not applicable. The analysis used a single baseline Lp(a) measurement and did not split follow up time according to changing exposure status.
1.2 **If Y/PY to 1.1**: Did the authors control for all the important baseline and time-varying confounding factors for which this was necessary?	NA	
1.3 **If Y/PY/WN to 1.2**: Were confounding factors that were controlled for (and for which control was necessary) measured validly and reliably by the variables available in this study?	NA	
1.4 **If N/PN/NI to 1.1**: Did the authors control for time-varying factors or other variables measured after the start of the exposure window being studied?	NA	
1.5 Did the use of negative controls, or other considerations, suggest uncontrolled confounding?	NA	
Risk of bias (due to confounding) in the estimated effect of exposure on the outcome	NA	
What is the predicted direction of bias due to confounding?	NA	
Is the risk of bias (due to confounding) sufficiently high, in the context of its likely direction and the magnitude of the estimated exposure effect, to threaten conclusions about whether the exposure has an important effect on the outcome?	NA	

Y = Yes; PY = Probably yes; PN = Probably no; N = No; SY = Strong yes; WY = Weak yes; SN = Strong no; WN = Weak no; NA = Not applicable; NI = No information

Domain 2: Risk of bias arising from measurement of the exposure

Domain 2, Variant (a): ***If N/PN to C5*** *(exposure was measured at a single point in time)*

Signalling questions	Response options	Comments
Mismeasurement or misclassification of the exposure.		
2.1 Does the measured exposure well-characterize the exposure metric specified to be of interest in this study? [*This was specified in the answers to D2, D3 and D4*]	PY	The single fasting baseline measurement probably characterizes usual Lp(a) reasonably well because Lp(a) is largely genetically determined and relatively stable. However, no repeated Lp(a) measurements were used.
2.2 Was the exposure likely to be measured with error, or misclassified?	WY	Lp(a) was measured centrally with an immunoenzymatic assay, but the paper does not state whether the assay was insensitive to apo(a) isoform size. Older Lp(a) assays may be affected by apo(a) size variation. Some measurement error is therefore possible.
Bias in the estimated effect of exposure arising from mismeasurement or misclassification of the exposure		
2.3 If SY/WY to 2.2: Could mismeasurement or misclassification of exposure have been differential (i.e. related to the outcome or risk of the outcome)?	PN	Exposure measurement occurred at baseline before incident prostate cancer diagnosis, and laboratory measurement was unlikely to depend on future cancer status.
2.4 If SY/WY to 2.2 and N/PN/WY to 2.3: Is non-differential measurement error likely to bias the estimated effect of exposure on outcome?	WY	Non differential exposure error could weaken any true association. Categorizing Lp(a) into quartiles may also reduce information and widen uncertainty, especially with few prostate cancer events.
Risk of bias (arising from measurement of exposure) in the estimated effect of exposure on the outcome	Some concerns	The exposure measurement was appropriate and prospectively obtained, but there are some concerns due to one measurement, quartile categorization, and limited assay detail regarding apo(a) isoform sensitivity.
What is the predicted direction of bias arising from measurement of exposure?	Towards null	Non differential measurement error and broad exposure categories would most likely attenuate a true association.
Is the risk of bias (arising from measurement of exposure) sufficiently high, in the context of its likely direction and the magnitude of the estimated exposure effect, to threaten conclusions about whether the exposure has an important effect on the outcome?	No	The exposure measurement concerns are not judged large enough to invalidate the result.

Y = Yes; PY = Probably yes; PN = Probably no; N = No; SY = Strong yes; WY = Weak yes; SN = Strong no; WN = Weak no; NA = Not applicable; NI = No information

*Domain 2, Variant (b): **If Y/PY to C5 and Y/PY to C6** (each individual's exposure level was estimated from measurements made at multiple time points)*

Signalling questions	Response options	Comments
2.1 Does the measured exposure (derived from measurements at multiple time points) well-characterize the exposure metric specified to be of interest in this study? [*This was specified in the answers to D2, D3 and D4*]	NA	Not applicable. The prostate cancer analysis used baseline Lp(a) measured at one time point.
2.2 Was there error in measurement, or misclassification, of the exposure, at each single time point?	NA	Not applicable. The prostate cancer analysis used baseline Lp(a) measured at one time point.
2.3 If SY/WY to 2.2: Could mismeasurement or misclassification of exposure have been differential (i.e. related to the outcome or risk of the outcome)?	NA	Not applicable. The prostate cancer analysis used baseline Lp(a) measured at one time point.
2.4 If SY/WY to 2.2 and N/PN/WY to 2.3: Is the nature of the (non-differential) measurement error likely to bias the estimated effect of exposure on outcome?	NA	Not applicable. The prostate cancer analysis used baseline Lp(a) measured at one time point.
Risk of bias (arising from measurement of exposure) in the estimated effect of exposure on the outcome	NA	Not applicable. The prostate cancer analysis used baseline Lp(a) measured at one time point.
What is the predicted direction of bias arising from measurement of exposure?	NA	Not applicable. The prostate cancer analysis used baseline Lp(a) measured at one time point.
Is the risk of bias (arising from measurement of exposure) sufficiently high, in the context of its likely direction and the magnitude of the estimated exposure effect, to threaten conclusions about whether the exposure has an important effect on the outcome?	NA	Not applicable. The prostate cancer analysis used baseline Lp(a) measured at one time point.

Y = Yes; PY = Probably yes; SN = Strong no; WN = Weak no; NA = Not applicable; NI = No information

*Domain 2, Variant (c): **If Y/PY to C5, N/PN to C6 and Y/PY to C7** (the analysis was based on splitting participants' follow up time according to exposure status and/or magnitude):*

Signalling questions	Response options	Comments
2.1 Does the measured exposure (including changes over time) well-characterize the exposure metric specified to be of interest in this study? [*This was specified in the answers to D2, D3 and D4*]	NA	Not applicable. The prostate cancer analysis used baseline Lp(a) measured at one time point.
2.2 Was there error in measurement, or misclassification, of the exposure, at each single time point?	NA	
2.3 If SY/WY to 2.2: Could mismeasurement or misclassification of exposure have been differential (i.e. related to the outcome or risk of the outcome)?	NA	
2.4 If SY/WY to 2.2 and N/PN/WY to 2.3: Is the nature of the (non-differential) measurement error likely to bias the estimated effect of exposure on outcome?	NA	
Risk of bias (arising from measurement of exposure) in the estimated effect of exposure on the outcome	NA	
What is the predicted direction of bias<br><br>arising from measurement of exposure?	NA	
Is the risk of bias (arising from measurement of exposure) sufficiently high, in the context of its likely direction and the magnitude of the estimated exposure effect, to threaten conclusions about whether the exposure has an important effect on the outcome?	NA	

Y = Yes; PY = Probably yes; SN = Strong no; WN = Weak no; NA = Not applicable; NI = No information

Domain 3: Risk of bias in selection of participants into the study (or into the analysis)

Signalling questions	Response options	Comments
3.1 Did follow-up begin at (or close to) the start of the exposure window for most participants? [*The exposure window is specified in D3*]	Y	Follow up began at baseline, close to the fasting blood draw used to measure Lp(a).
3.2 **If N/PN to 3.1**: Is the effect of exposure likely to be constant over the period of follow up analysed?	NA	Not applicable because follow up began at or close to the measured exposure time.
3.3 Was selection of participants into the study (or into the analysis) based on participant characteristics observed after the start of the exposure window being studied? [*The exposure window is specified in D3*]	PN	Selection into the analysis was based mainly on baseline recruitment, absence of former or current cancer, and availability of Lp(a). Excluding 41 men without Lp(a) was unlikely to be based on later prostate cancer status.
3.4 **If Y/PY to 3.3**: Were these characteristics likely to be influenced by exposure or a cause of exposure?	NA	Not applicable because selection into the analysis was probably not based on participant characteristics observed after the start of the measured exposure window.
3.5 **If Y/PY to 3.4**: Were these characteristics likely to be influenced by the outcome or a cause of the outcome?	NA	Not applicable.
3.6 **If N/PN to 3.2 or Y/PY to 3.5**: Is it likely that the analysis corrected for all of the potential selection biases identified in A and B above?	NA	Not applicable.
3.7 **If N/PN to 3.2 or Y/PY to 3.5**: Did sensitivity analyses demonstrate that the likely impact of the potential selection biases identified in A or B above was minimal?	NA	Not applicable.
Risk of bias (due to selection of participants into the study) in the estimated effect of exposure on the outcome	Low risk	Selection into the analysis is unlikely to have introduced major bias for the prostate cancer association. Recruitment from health screening and occupational settings may limit generalizability, but it is less likely to distort the within cohort Lp(a) association.
What is the predicted direction of bias<br><br>due to selection of participants into the study?	Insufficient information	No important selection bias direction is evident.
Is the risk of bias (due to selection of participants into the study) sufficiently high, in the context of its likely direction and the magnitude of the estimated exposure effect, to	No	Selection bias is unlikely to threaten the study conclusion.

Signalling questions	Response options	Comments
threaten conclusions about whether the exposure has an important effect on the outcome?		

Y = Yes; PY = Probably yes; PN = Probably no; N = No; SN = Strong no; WN = Weak no; NA = Not applicable; NI = No information

Domain 4: Risk of bias due to post-exposure interventions

Signalling questions	Response options	Comments
4.1 Were there post-exposure interventions that were influenced by prior exposure during the follow-up period?	PY	Post baseline medical care, PSA testing, and lipid lowering treatment could have occurred during follow up. These may have been influenced by baseline cardiovascular risk profile, dyslipidaemia, or health care contact, which could be related to Lp(a).
4.2 **If Y/PY to 4.1**: Is it likely that the analysis corrected for the effect of post-exposure interventions that were influenced by prior exposure?	PN	The analysis did not adjust for post baseline PSA testing, changes in lipid lowering therapy, or medical surveillance during follow up.
Risk of bias (due post-exposure interventions) in the estimated effect of exposure on the outcome	Some concerns	There is some concern that post baseline PSA testing and medical surveillance could influence prostate cancer diagnosis. The measured Lp(a) value itself was probably not returned for cancer decision making, but broader cardiovascular risk monitoring could matter.
What is the predicted direction of bias due to confounding?	Insufficient information	The direction is uncertain. More medical contact could increase diagnosis, but the relation between Lp(a) and later surveillance is not known.
Is the risk of bias (due post-exposure interventions) sufficiently high, in the context of its likely direction and the magnitude of the estimated exposure effect, to threaten conclusions about whether the exposure has an important effect on the outcome?	Cannot tell	This concern could influence prostate cancer incidence estimates, but the available information is insufficient to judge its magnitude.

Y = Yes; PY = Probably yes; PN = Probably no; N = No; NA = Not applicable; NI = No information

Domain 5: Risk of bias due to missing data

Signalling questions	Response options	Comments
5.1 Were complete data on exposure status available for all, or nearly all, participants?	Y	Of 5237 recruited men, 41 had missing Lp(a) and were excluded. Thus Lp(a) data were available for nearly all participants.
5.2 Were complete data on the outcome available for all, or nearly all, participants?	PY	Annual follow up was conducted for 10 years. During follow up, 209 men, equal to 4.0 percent, were lost to follow up. Outcome data were therefore available for most participants.
5.3 Were complete data on confounding variables available for all, or nearly all, participants?	Y	Total missing data for non biological covariates were reported as only 0.2 percent.
5.4 **If N/PN/NI to 5.1, 5.2 or 5.3**: Is the result based on a complete case analysis?	NA	Not applicable because exposure, outcome, and confounder data were available for all or nearly all participants.
5.5 **If Y/PY/NI**: Was exclusion from the analysis because of missing data (in exposure, confounders or the outcome) likely to be related to the true value of the outcome?	NA	Not applicable.
5.6 **If N/PN to 5.5**: Were all or most predictors of missingness (in exposure, confounders or the outcome) included in the analysis model?	NA	Not applicable.
5.7 **If N/PN to 5.4**: Was the analysis based on imputing missing values?	NA	Not applicable.
5.8 **If Y/PY to 5.7**: Was imputation performed appropriately?	NA	Not applicable.
5.9 **If N/PN to 5.7**: Was an appropriate alternative method used to correct for bias due to missing data?	NA	Not applicable.
5.10 **If PN/N/NI to 5.1, 5.2 or 5.3**: Is there evidence that the result was not biased by missing data?	Y	The amount of missing exposure and covariate data was small, and loss to follow up was limited. There is no evidence that missing data materially biased the prostate cancer result.
Risk of bias (due to missing data) in the estimated effect of exposure on the outcome	Low risk	Missing data were limited: fewer than 1 percent missing Lp(a), 0.2 percent missing non biological covariates, and 4 percent lost to follow up.

Signalling questions	Response options	Comments
What is the predicted direction of bias due to missing data?	Insufficient information	No important direction of missing data bias is evident.
Is the risk of bias (due to missing data) sufficiently high, in the context of its likely direction and the magnitude of the estimated exposure effect, to threaten conclusions about whether the exposure has an important effect on the outcome?	No	Missing data are unlikely to threaten the conclusion.

Y = Yes; PY = Probably yes; PN = Probably no; N = No; SY = Strong yes; WY = Weak yes; NA = Not applicable; NI = No information

Domain 6: Risk of bias arising from measurement of the outcome

Signalling questions	Response options	Comments
6.1 Could measurement or ascertainment of the outcome have differed between exposure groups or levels of exposure?	PN	Cancer case identification used annual questionnaires followed by medical inquiry, with histological confirmation systematically sought. The method is unlikely to have differed by baseline Lp(a) quartile, although underlying PSA testing frequency could differ and is considered under confounding and post baseline interventions.
6.2 Were outcome assessors aware of study participants' exposure history?	PN	Trained medical staff confirming cancer diagnoses were unlikely to be aware of or influenced by baseline Lp(a) levels.
6.3 **If Y/PY/NI to 6.2**: Could assessment of the outcome have been influenced by knowledge of participants' exposure history?	NA	Not applicable because outcome assessors were probably not aware of Lp(a) exposure status.
Risk of bias (arising from measurement of outcomes) in the estimated effect of exposure on the outcome	Low risk	Outcome measurement was appropriate. Histological confirmation was obtained for 96.1 percent of cancer cases, and cancer sites were classified using ICD 9 and FRANCIM methods.
What is the predicted direction of bias arising from measurement of outcomes?	Insufficient information available	No important bias from outcome measurement is expected.
Is the risk of bias (arising from measurement of outcomes) sufficiently high, in the context of its likely direction and the magnitude of the estimated exposure effect, to threaten conclusions about whether the exposure has an important effect on the outcome?	No	Outcome measurement is unlikely to threaten the conclusion.

Y = Yes; PY = Probably yes; PN = Probably no; N = No; SY = Strong yes; WY = Weak yes; NA = Not applicable; NI = No information

Domain 7: Risk of bias in selection of the reported result

Signalling questions	Response options	Comments
7.1 Was the result reported in accordance with an available, pre-determined analysis plan?	NI	No publicly available result specific analysis plan was reported for the Lp(a) and prostate cancer result. The PRIME cohort methods were described elsewhere, but the cancer analysis appears to have been conducted as an observational secondary analysis.
7.2 **If N/PN/NI to 7.1**: Is the reported effect estimate likely to be selected, based on desirability of the magnitude (or statistical significance) of the estimated effect of exposure on outcome, from multiple *exposure measurements* within the exposure domain?	PN	The study reported all main site specific cancer results, including the non significant prostate cancer finding. This reduces concern that the prostate result was selectively reported because it was desirable.
7.3 Is the reported effect estimate likely to be selected, based on desirability of the magnitude (or statistical significance) of the estimated effect of exposure on outcome, from multiple *outcome measurements* within the outcome domain?	PN	Incident prostate cancer was one of the main cancer sites and was classified using standard cancer coding. There is no clear evidence that the outcome definition was selected based on the result.
7.4 Is the reported effect estimate likely to be selected, based on desirability of the magnitude (or statistical significance) of the estimated effect of exposure on outcome, from multiple *analyses* of the exposure-outcome relationship?	PY	Several analyses were conducted, including all cancer, main cancer sites, tobacco and alcohol related cancers, BMI interaction, exclusion of early cases, and exclusion of lipid lowering drug users. No formal multiple testing correction was reported, and the selected emphasis in the discussion could be influenced by observed patterns.
7.5 Is the reported effect estimate likely to be selected, based on the basis of desirability of the results (e.g. statistical significance), from different *subgroups*?	PY	Quartiles and tertiles were based on the observed Lp(a) distribution, and multiple model choices and subgroup analyses were explored. For the prostate result, Table 5 provides the full quartile estimates, but concerns about analysis selection remain.
Risk of bias (due to selection of the reported result) in the estimated effect of exposure on the outcome	Some concerns	There is some concern because no result specific analysis plan was available, multiple cancer sites and subgroup analyses were examined, and no formal multiple testing correction was reported. However, the prostate cancer result itself was reported transparently despite being non significant.
What is the predicted direction of bias due to selection of the reported result?	Insufficient information available	The direction of bias from selection of the reported result is uncertain.

Signalling questions	Response options	Comments
Is the risk of bias (due to selection of the reported result) sufficiently high, in the context of its likely direction and the magnitude of the estimated exposure effect, to threaten conclusions about whether the exposure has an important effect on the outcome?	No	This domain is not judged high enough to invalidate the reported prostate cancer result.

Y = Yes; PY = Probably yes; PN = Probably no; N = No; NA = Not applicable; NI = No information

Overall risk of bias

	Response options	Comments
Overall risk of bias	Some concerns	Overall judgement: Some concerns. The study has important strengths, including prospective design, baseline fasting Lp(a) measurement, exclusion of participants with prior cancer, 10 years of follow up, low missingness, and medically confirmed cancer diagnoses. Main concerns are residual confounding and detection bias from unmeasured PSA testing, family history, hormone related factors, and health care contact; limited adjustment in the prostate cancer model; possible post baseline diagnostic surveillance effects; limited assay detail for Lp(a); and multiple comparisons without a prespecified analysis plan.
What is the predicted direction of bias?	Insufficient information available	Measurement error would most likely bias towards the null, but detection bias and residual confounding could act in either direction. The observed highest quartile estimate was below the null but imprecise.
Is the overall risk of bias sufficiently high, in the context of its likely direction and the magnitude of the estimated exposure effect, to threaten conclusions about whether the exposure has an important effect on the outcome?	Cannot tell	The overall risk of bias is not so high that the study should be excluded, but the concerns are important for interpretation. The prostate cancer result was imprecise, non significant, and based on 126 cases, so it should be interpreted as limited evidence rather than definitive evidence for absence of association.

This work is licensed under a Creative Commons Attribution-NonCommercial-NoDerivatives 4.0 International License.
