# Supplementary material for: Lipoprotein(a) and Prostate Cancer: A Systematic Review of Observational, Genetic and Mendelian Randomization Evidence
Source: Int J Mol Sci. 2026 Sep 17;27(18):8274. doi: 10.3390/ijms27188274 (PMC13607466; doi:10.3390/ijms27188274)
Supplement: Supplementary file 1 [file ijms-27-08274-s001.zip › Supplementary Material S5.pdf]

The Risk Of Bias In Non-randomized Studies – of Exposures (ROBINS-E) assessment tool
(for follow-up studies)

Template for completion

Version 24 March 2024

This work is licensed under a Creative Commons Attribution-NonCommercial-NoDerivatives 4.0 International License.

The ROBINS-E tool

At planning stage: list confounding factors and consider appropriateness criteria

P1. List the important confounding factors relevant to all or most studies on this topic. Specify whether these are particular to specific exposures-outcome combinations.

Age, ethnicity or genetic ancestry, family history and inherited susceptibility to prostate cancer, socioeconomic position, access to health care, PSA testing and screening behaviour, BMI and adiposity, diabetes and metabolic health, smoking, alcohol intake, physical activity, diet, lipid lowering medication, cardiovascular disease and health care contact, renal disease, liver disease, and inflammation. PSA testing and health care contact are particularly important for prostate cancer diagnosis because they may influence detection. Ethnicity or genetic ancestry is important for Lp(a) because circulating concentrations differ strongly by genetic background.

P2. Will the review use the ROBINS-E assessment of appropriateness (important aspects of "study sensitivity")?

No

If Yes, complete sections Addressing appropriateness, Parts I and II in Appendix 1.

For each study result: preliminary considerations

A. Specify the result being assessed for risk of bias

A1. Specify the numerical result being assessed

Fully adjusted Cox regression estimate for baseline serum Lp(a) concentration and incident prostate cancer, corrected for regression dilution bias. HR per 1 SD higher Lp(a): 1.02, 95% CI 0.99 to 1.05. A prostate cancer mortality estimate was also reported, HR per 1 SD higher Lp(a): 1.03, 95% CI 0.91 to 1.16, but this assessment focuses on incident prostate cancer.

B. Decide whether to proceed with a risk-of-bias assessment

	Response options	Comments
B1. Did the authors make any attempt to control for confounding?	Y	The authors used Cox proportional hazards models with age as the underlying time variable. The fully adjusted model was stratified by geographical region and age group and adjusted for Townsend deprivation score, ethnicity, living with a wife or partner, BMI, smoking, physical activity, alcohol consumption, and diabetes.
B2. **If N/PN to B1**: Is there sufficient potential for confounding that an unadjusted result should not be considered further?	NA	Not applicable because the authors made an attempt to control for confounding.
B3. Was the method of measuring exposure inappropriate?	N	The exposure was serum Lp(a) concentration measured from baseline blood samples collected at recruitment. UK Biobank laboratory methods and quality control procedures were referenced, and repeat biomarker data from a subsample were used to correct for regression dilution bias.
B4. Was the method of measuring the outcome inappropriate?	N	Incident prostate cancer was defined as first diagnosis of prostate cancer using ICD 10 code C61 or death from prostate cancer, whichever occurred first. Outcomes were obtained through linked health administrative datasets, cancer registries, and death records.

If the answer to any of B2, B3 or B4 is 'Yes' or 'Probably yes', the result should be considered to be at very high risk of bias and no further assessment is required. Otherwise, proceed to section C.

C. Specify the analysis in the current study for which results are being assessed for risk of bias

C1. Specify the outcome to which this result relates.

Incident prostate cancer, defined as first diagnosis of prostate cancer using ICD 10 code C61 or death from prostate cancer, whichever occurred first.

C2. Specify the participant group on which this result was based.

Men in UK Biobank included in the biomarker analysis after exclusions, recruited from 2006 to 2010 at ages 40 to 69 years. The full analytic cohort included 211,754 men, with fewer participants in the Lp(a) analysis because of biomarker availability. Follow up for prostate cancer incidence used linked health administrative data.

C3 to C8: Describe the exposure measurement(s) used to produce this result.

C3. What is the exposure being measured and how was it measured or assessed?	Baseline serum Lp(a) concentration measured from blood collected at recruitment. Samples were nonfasting and processed through the UK Biobank central laboratory procedures. Lp(a) was reported in nmol per L. Repeat blood samples in a subsample were used to estimate regression dilution and correct risk estimates.
C4. Was exposure analysed as a quantitative (rather than a categorical) variable?	Y
C5. Did repeated measurements of exposure over time (for each participant) contribute to the analysis that produced this result?	PY
C6. **If Y/PY to C5**, was a single estimate of each participant's exposure level derived from the repeated measurements of exposure over time?	PN
C7. **If N/PN to C6**, was the analysis based on splitting participants' follow up time according to exposure status and/or magnitude?	N
C8. **If Y/PY to C7**, were changes in exposure status and/or magnitude likely to be related to factors that are predictive of the outcome?	NA

C9. **If N/PN to C7**, how were repeat measurements used?

Repeat measurements were not used to split follow up time or to derive participant specific time varying exposure values. They were used in a subsample to estimate regression dilution ratios and to correct HRs for usual biomarker concentration.

Y = Yes; PY = Probably yes; PN = Probably no; N = No; NA = Not applicable

C10. Specify the relationship analysed to produce this result. For example, this may be a quadratic relationship of cumulative exposure with the log odds of the outcome, or a risk ratio for the outcome comparing exposed with unexposed individuals.

Fully adjusted Cox proportional hazards model estimating the HR for incident prostate cancer per 1 SD higher log transformed usual serum Lp(a) concentration. Models used age as the underlying time variable, were stratified by geographical region and age at recruitment, and adjusted for socioeconomic, anthropometric, lifestyle, and diabetes variables. The reported estimate was corrected for regression dilution bias.

D: Specify the causal effect of exposure being estimated by this result

D1. Specify the population of interest	Describe eligible participants (to whom the causal effect applies). These may be different from the study participants on whom the result was based (specified in C2). Such differences may give rise to selection biases.	Adult men without known prostate cancer at baseline in a general population cohort setting similar to UK Biobank. The effect of interest is the association between higher usual circulating Lp(a) before diagnosis and subsequent prostate cancer diagnosis.
Specification of the exposure metric of interest		
D2. Specify the exposure	This is the factor whose causal effect on the outcome of interest is the subject of the study result being assessed. It may be thought of as the 'true' exposure of interest. It is distinct from the method with which exposure was measured.	Higher usual circulating Lp(a) concentration. This represents the biological exposure of interest, distinct from a single laboratory measurement.
D3. Specify the exposure window	The exposure window of interest is the exposure period for which the result being assessed estimates the effect of exposure on the outcome. Specification of the exposure window is judged by the ROBINS-E user, who should aim to define a window that is both meaningful in answering the review question and broadly in line with when the study measured exposure. Specification should include both the time of onset and period of exposure. For example, it may be lifetime exposure (from birth or from conception), during ages 50-55, the period from first employment in a particular occupation, time from birth to age 10, or during pregnancy.<br><br>The specified exposure window is used to determine whether exposure data adequately reflect exposure during the window. Exposure before the start of the exposure window is addressed during the assessment of risk of bias due to confounding	Usual circulating Lp(a) before prostate cancer diagnosis, beginning before UK Biobank recruitment and represented by the baseline blood sample. Because Lp(a) is largely genetically determined and relatively stable, the baseline measurement is intended to reflect usual exposure before diagnosis.
D4. Specify how exposure over time	This may, for example, be ever/never exposed, cumulative exposure, average exposure, or peak exposure during the exposure period, for each participant. Alternatively, there may be only a	Usual serum Lp(a) concentration summarized as a continuous log transformed biomarker, with the main assessed contrast expressed per 1

should be summarized	single exposure event, or the exposure may be time invariant (such as a genetic variant or family history).	SD higher concentration. The study also presented fifths of biomarker concentration for incidence analyses.

E. Evaluation of confounding factors

Complete a row for each important confounding factor listed in advance (subsection (i)). In addition, consider any further confounding factors that are either relevant to the setting of this particular study or which the study authors identified as potentially important (subsection (ii)).

"Important" confounding factors are those for which, in the context of this study, adjustment is expected to lead to an important change in the estimated effect of the exposure.

(i) Important confounding factors listed in advance						
Confounding factor	Measured variable(s) for this factor, if any	Was this variable (or were these variables) controlled for in the analysis?<br>(Y / N)	If this confounding factor was controlled for, was it measured validly and reliably by this variable (or these variables)?*<br>(NA / Y / PY / PN / N / NI)	If this confounding factor was not controlled for, is there evidence that controlling for it was unnecessary?**<br>(NA / Y / PY / PN / N)	Is failure to adjust for this confounding factor expected to bias the effect estimate towards benefit or harm of (higher) exposure?***<br>(Benefit of (higher) exposure / Harm of (higher) exposure / Insufficient information available)	Comments
Age	Age at recruitment and attained age in Cox model	Y	Y	NA	Insufficient information available	Age is a strong determinant of prostate cancer incidence and was handled through age as the underlying time variable and stratification by age group.
Ethnicity or genetic ancestry	Self reported ethnic group categories	Y	PY	NA	Insufficient information available	Important because Lp(a) concentrations and prostate cancer risks differ by genetic background. Broad categories were controlled, but residual ancestry differences may remain.
Socioeconomic position and access to health care	Townsend deprivation score; living with a wife or partner	Y	PY	NA	Insufficient information	Controlled partly. These variables may not fully capture health care access or use of PSA testing.

PSA testing and screening behaviour	Not measured	N	NA	N	Risk of higher exposure	A key concern for prostate cancer diagnosis. If men with higher Lp(a) had more cardiovascular care or health monitoring, they might have more opportunity for PSA testing and cancer detection.
Family history and inherited susceptibility	Not measured	N	NA	N	Insufficient information	Family history is a strong prostate cancer risk factor and may correlate with health seeking behaviour. No evidence was provided that adjustment was unnecessary.
BMI and adiposity	BMI category; height	Y	Y	NA	Insufficient information	Anthropometric measurements were collected at baseline by trained staff, with low missingness handled by an unknown category.
Smoking	Self reported smoking status and cigarettes per day	Y	PY	NA	Insufficient information	Controlled in the fully adjusted model. Some self report error is possible but unlikely to be substantial.

Alcohol intake	Self reported alcohol consumption in grams of ethanol per day	Y	PY	NA	Insufficient information	Controlled in the fully adjusted model. Measurement error is possible but probably limited for broad categories.
Physical activity	Physical activity in METs per week	Y	PY	NA	Insufficient information	Controlled in the fully adjusted model. Self reported activity may have some error.
Diabetes and metabolic health	Self reported or recorded diabetes status	Y	PY	NA	Insufficient information	Diabetes was controlled and is relevant because it may influence prostate cancer diagnosis patterns and biomarker profiles.
Lipid lowering medication	Not included in the fully adjusted model	N	NA	PY	Insufficient information	Most standard lipid lowering medicines have limited direct effect on Lp(a), but medication use may reflect health care contact. Failure to adjust is a minor concern.

(ii) Additional confounding factors relevant to the setting of this particular study, or identified by study authors and considered to be important, or which were identified since the protocol was written						
Confounding factor	Measured variable(s) for this factor, if any	Was this variable (or were these variables) controlled for in the analysis?<br>(Y / N)	If this confounding factor was controlled for, was it measured validly and reliably by this variable (or these variables)?*<br>(NA / Y / PY / PN / N / NI)	If this confounding factor was not controlled for, is there evidence that controlling for it was unnecessary?**<br>(NA / Y / PY / PN / N)	Is failure to adjust for this confounding factor expected to bias the effect estimate towards benefit or harm of (higher) exposure?*** (Benefit of (higher) exposure / Harm of (higher) exposure / Insufficient information available)	Comments
Region of recruitment	Ten UK recruitment regions	Y	Y	NA	Insufficient information	Models were stratified by geographical region of recruitment.
Renal and liver disease	Some related biomarkers were measured; clinical disease status not controlled for Lp(a) model	N	NA	PN	Insufficient information	Renal and liver function can influence biomarker concentrations and health status. The impact on Lp(a) is probably limited but not fully addressed.
Inflammation and general health status	C reactive protein measured but not used as a confounder in the Lp(a) model	N	NA	PN	Insufficient information	Inflammation may relate to cancer detection or general health. It is unclear whether adjustment would be appropriate or necessary.

* “Validity” refers to whether the confounding variable or variables accurately measure the confounding factor, while “reliability” refers to the precision of the measurement (more measurement error means less reliability).

** In the context of a particular study, variables need not be included in the analysis: (a) if they are measured validly and reliably and are not associated with the outcome, conditional on exposure (noting that lack of a statistically significant association is not evidence of a lack of association); (b) if they are measured validly and reliably and are not associated with exposure; (c) if they are measured validly and reliably and

adjustment makes no or minimal difference to the estimated effect of the primary parameter; (d) because the confounder was addressed in the study design, for example by restricting to individuals with the same value of the confounder; (e) because a negative control demonstrates that there was unlikely to have been confounding due to this variable or that uncontrolled confounding was likely to be minimal; or (f) because external evidence suggests that controlling for the variable is not necessary in the context of the study being assessed..

For each study: risk of bias assessment

Domain 1: Risk of bias due to confounding

*Domain 1, Variant (a): **If N/PN to C5 or Y/PY to C6 or N/PN to C7** (only baseline confounding needs to be addressed)*

Signalling questions	Response options	Comments
1.1 Did the authors control for all the important confounding factors for which this was necessary?	WN	The authors controlled for many important confounders including age, region, socioeconomic position, ethnicity, partner status, BMI, smoking, physical activity, alcohol consumption, and diabetes. However, PSA testing, family history, and lipid lowering medication were not fully controlled. These omissions are important for prostate cancer detection, although their relation to Lp(a) is uncertain.
1.2 **If Y/PY/WN to 1.1**: Were confounding factors that were controlled for (and for which control was necessary) measured validly and reliably by the variables available in this study?	WN	Most controlled factors were measured at baseline using questionnaires, interviews, or trained staff measurements. Anthropometry was likely reliable. Lifestyle variables and diabetes status may have some measurement error, but the extent is probably not substantial.
1.3 **If Y/PY/WN to 1.1**: Did the authors control for any variables after the start of the exposure period being studied that could have been affected by the exposure?	N	The adjustment variables were baseline covariates measured at recruitment, not variables measured after the start of follow up that would plausibly be affected by measured Lp(a).
1.4 Did the use of negative controls, or other considerations, suggest serious uncontrolled confounding?	PN	No negative control analysis was reported. Sensitivity analyses addressed reverse causation by excluding the first 4 years of follow up, but did not directly address PSA testing or family history.
Risk of bias (due to confounding) in the estimated effect of exposure on the outcome	Some concerns	Residual confounding and detection bias remain possible, mainly through unmeasured PSA testing behaviour, family history, and health care contact. The extensive baseline adjustment reduces but does not remove this concern.
What is the predicted direction of bias due to confounding?	Insufficient information available	Potential bias could operate in different directions. More health care contact among men with high cardiovascular risk might increase prostate cancer detection, but the relation between Lp(a) and such contact was not directly evaluated.

Signalling questions	Response options	Comments
Is the risk of bias (due to confounding) sufficiently high, in the context of its likely direction and the magnitude of the estimated exposure effect, to threaten conclusions about whether the exposure has an important effect on the outcome?	No	The concern is important for interpretation, but the Lp(a) estimate for incident prostate cancer was close to the null, so confounding is not judged high enough by itself to threaten the conclusion that there is little evidence of association in this study.

Y = Yes; PY = Probably yes; PN = Probably no; N = No; SY = Strong yes; WY = Weak yes; SN = Strong no; WN = Weak no; NA = Not applicable; NI = No information

*Domain 1, variant (b): **If Y/PY to C7 and Y/PY to C8** (the analysis was based on splitting participants' follow up time according to exposure status and/or magnitude and changes in exposure status and/or magnitude likely to be related to factors that are predictive of the outcome, so both baseline and time-varying confounding need to be addressed)*

Signalling questions	Response options	Comments
1.1 Did the authors use an analysis method that was appropriate to control for time-varying as well as baseline confounding?	NA	Not applicable. The analysis did not split follow up according to changing Lp(a) status or magnitude.
1.2 **If Y/PY to 1.1**: Did the authors control for all the important baseline and time-varying confounding factors for which this was necessary?	NA	Not applicable. The analysis did not split follow up according to changing Lp(a) status or magnitude.
1.3 **If Y/PY/WN to 1.2**: Were confounding factors that were controlled for (and for which control was necessary) measured validly and reliably by the variables available in this study?	NA	Not applicable. The analysis did not split follow up according to changing Lp(a) status or magnitude.
1.4 **If N/PN/NI to 1.1**: Did the authors control for time-varying factors or other variables measured after the start of the exposure window being studied?	NA	Not applicable. The analysis did not split follow up according to changing Lp(a) status or magnitude.
1.5 Did the use of negative controls, or other considerations, suggest uncontrolled confounding?	NA	Not applicable. The analysis did not split follow up according to changing Lp(a) status or magnitude.
Risk of bias (due to confounding) in the estimated effect of exposure on the outcome	NA	Not applicable. The analysis did not split follow up according to changing Lp(a) status or magnitude.
What is the predicted direction of bias due to confounding?	NA	Not applicable. The analysis did not split follow up according to changing Lp(a) status or magnitude.
Is the risk of bias (due to confounding) sufficiently high, in the context of its likely direction and the magnitude of the estimated exposure effect, to threaten conclusions about whether the exposure has an important effect on the outcome?	NA	Not applicable. The analysis did not split follow up according to changing Lp(a) status or magnitude.

Y = Yes; PY = Probably yes; PN = Probably no; N = No; SY = Strong yes; WY = Weak yes; SN = Strong no; WN = Weak no; NA = Not applicable; NI = No information

Domain 2: Risk of bias arising from measurement of the exposure

Domain 2, Variant (a): ***If N/PN to C5*** *(exposure was measured at a single point in time)*

Signalling questions	Response options	Comments
Mismeasurement or misclassification of the exposure.		
2.1 Does the measured exposure well-characterize the exposure metric specified to be of interest in this study? [*This was specified in the answers to D2, D3 and D4*]	PY	Baseline serum Lp(a) is a reasonable marker of usual Lp(a), especially because Lp(a) is relatively stable over time. Repeat samples in a subsample were used to correct regression dilution bias. However, one baseline measurement may not perfectly reflect usual concentration for every participant.
2.2 Was the exposure likely to be measured with error, or misclassified?	WY	Laboratory measurement used UK Biobank biomarker procedures, but the article does not provide full assay details in the main text, including whether the Lp(a) assay was fully insensitive to apo(a) isoform size. Some laboratory error or biological variation is possible.
Bias in the estimated effect of exposure arising from mismeasurement or misclassification of the exposure		
2.3 If SY/WY to 2.2: Could mismeasurement or misclassification of exposure have been differential (i.e. related to the outcome or risk of the outcome)?	PN	Lp(a) was measured from blood samples collected before prostate cancer diagnosis, and laboratory measurement was unlikely to depend on later outcome status.
2.4 If SY/WY to 2.2 and N/PN/WY to 2.3: Is non-differential measurement error likely to bias the estimated effect of exposure on outcome?	WY	Any non differential measurement error would be expected mainly to attenuate associations towards the null. Correction for regression dilution bias reduces this concern but may not remove all measurement error.
Risk of bias (arising from measurement of exposure) in the estimated effect of exposure on the outcome	Some concerns	Exposure measurement was prospective and supported by repeat measures for regression dilution correction. Some concerns remain because of reliance on a baseline measurement and limited assay detail in the main article.
What is the predicted direction of bias arising from measurement of exposure?	Towards null	Non differential measurement error would most likely weaken any true association.
Is the risk of bias (arising from measurement of exposure) sufficiently high, in the context of its likely direction and the magnitude of the estimated exposure effect, to threaten conclusions about whether the exposure has an important effect on the outcome?	No	The exposure measurement concerns are not judged large enough to threaten the main conclusion for Lp(a).

Y = Yes; PY = Probably yes; PN = Probably no; N = No; SY = Strong yes; WY = Weak yes; SN = Strong no; WN = Weak no; NA = Not applicable; NI = No information

*Domain 2, Variant (b): **If Y/PY to C5 and Y/PY to C6** (each individual's exposure level was estimated from measurements made at multiple time points)*

Signalling questions	Response options	Comments
2.1 Does the measured exposure (derived from measurements at multiple time points) well-characterize the exposure metric specified to be of interest in this study? [*This was specified in the answers to D2, D3 and D4*]	NA	Not applicable for this assessment. The participant exposure used for the main association was the baseline Lp(a) measurement; repeated samples were used only to correct regression dilution bias.
2.2 Was there error in measurement, or misclassification, of the exposure, at each single time point?	NA	Not applicable for this assessment. The participant exposure used for the main association was the baseline Lp(a) measurement; repeated samples were used only to correct regression dilution bias.
2.3 If SY/WY to 2.2: Could mismeasurement or misclassification of exposure have been differential (i.e. related to the outcome or risk of the outcome)?	NA	Not applicable for this assessment. The participant exposure used for the main association was the baseline Lp(a) measurement; repeated samples were used only to correct regression dilution bias.
2.4 If SY/WY to 2.2 and N/PN/WY to 2.3: Is the nature of the (non-differential) measurement error likely to bias the estimated effect of exposure on outcome?	NA	Not applicable for this assessment. The participant exposure used for the main association was the baseline Lp(a) measurement; repeated samples were used only to correct regression dilution bias.
Risk of bias (arising from measurement of exposure) in the estimated effect of exposure on the outcome	NA	Not applicable for this assessment. The participant exposure used for the main association was the baseline Lp(a) measurement; repeated samples were used only to correct regression dilution bias.
What is the predicted direction of bias arising from measurement of exposure?	NA	Not applicable for this assessment. The participant exposure used for the main association was the baseline Lp(a) measurement; repeated samples were used only to correct regression dilution bias.
Is the risk of bias (arising from measurement of exposure) sufficiently high, in the context of its likely direction and the magnitude of the estimated exposure effect, to threaten conclusions about whether the exposure has an important effect on the outcome?	NA	Not applicable for this assessment. The participant exposure used for the main association was the baseline Lp(a) measurement; repeated samples were used only to correct regression dilution bias.

Y = Yes; PY = Probably yes; SN = Strong no; WN = Weak no; NA = Not applicable; NI = No information

*Domain 2, Variant (c): **If Y/PY to C5, N/PN to C6 and Y/PY to C7** (the analysis was based on splitting participants' follow up time according to exposure status and/or magnitude):*

Signalling questions	Response options	Comments
2.1 Does the measured exposure (including changes over time) well-characterize the exposure metric specified to be of interest in this study? [*This was specified in the answers to D2, D3 and D4*]	NA	Not applicable for this assessment. The participant exposure used for the main association was the baseline Lp(a) measurement; repeated samples were used only to correct regression dilution bias.
2.2 Was there error in measurement, or misclassification, of the exposure, at each single time point?	NA	Not applicable for this assessment. The participant exposure used for the main association was the baseline Lp(a) measurement; repeated samples were used only to correct regression dilution bias.
2.3 If SY/WY to 2.2: Could mismeasurement or misclassification of exposure have been differential (i.e. related to the outcome or risk of the outcome)?	NA	Not applicable for this assessment. The participant exposure used for the main association was the baseline Lp(a) measurement; repeated samples were used only to correct regression dilution bias.
2.4 If SY/WY to 2.2 and N/PN/WY to 2.3: Is the nature of the (non-differential) measurement error likely to bias the estimated effect of exposure on outcome?	NA	Not applicable for this assessment. The participant exposure used for the main association was the baseline Lp(a) measurement; repeated samples were used only to correct regression dilution bias.
Risk of bias (arising from measurement of exposure) in the estimated effect of exposure on the outcome	NA	Not applicable for this assessment. The participant exposure used for the main association was the baseline Lp(a) measurement; repeated samples were used only to correct regression dilution bias.
What is the predicted direction of bias arising from measurement of exposure?	NA	Not applicable for this assessment. The participant exposure used for the main association was the baseline Lp(a) measurement; repeated samples were used only to correct regression dilution bias.
Is the risk of bias (arising from measurement of exposure) sufficiently high, in the context of its likely direction and the magnitude of the estimated exposure effect, to threaten conclusions about whether the exposure has an important effect on the outcome?	NA	Not applicable for this assessment. The participant exposure used for the main association was the baseline Lp(a) measurement; repeated samples were used only to correct regression dilution bias.

Y = Yes; PY = Probably yes; SN = Strong no; WN = Weak no; NA = Not applicable; NI = No information

Domain 3: Risk of bias in selection of participants into the study (or into the analysis)

Signalling questions	Response options	Comments
3.1 Did follow-up begin at (or close to) the start of the exposure window for most participants? [*The exposure window is specified in D3*]	Y	Follow up for prostate cancer incidence began at recruitment, close to the baseline blood draw used to define measured Lp(a).
3.2 **If N/PN to 3.1**: Is the effect of exposure likely to be constant over the period of follow up analysed?	NA	Not applicable because follow up began at or close to the measured exposure time.
3.3 Was selection of participants into the study (or into the analysis) based on participant characteristics observed after the start of the exposure window being studied? [*The exposure window is specified in D3*]	PY	Selection into UK Biobank depended on volunteering and attending assessment centres after many years of prior usual Lp(a) exposure. Selection into the Lp(a) analysis also depended on biomarker availability.
3.4 **If Y/PY to 3.3**: Were these characteristics likely to be influenced by exposure or a cause of exposure?	PY	Usual Lp(a) may influence cardiovascular morbidity and health care contact, which could influence willingness or ability to participate. This pathway is plausible but not demonstrated.
3.5 **If Y/PY to 3.4**: Were these characteristics likely to be influenced by the outcome or a cause of the outcome?	PY	Participant selection may also be related to factors influencing prostate cancer diagnosis, including health consciousness and health service use. The low UK Biobank participation rate increases this concern.
3.6 **If N/PN to 3.2 or Y/PY to 3.5**: Is it likely that the analysis corrected for all of the potential selection biases identified in A and B above?	PN	The analysis adjusted for several baseline characteristics, but it could not fully correct for healthy volunteer selection or selection related to biomarker availability.
3.7 **If N/PN to 3.2 or Y/PY to 3.5**: Did sensitivity analyses demonstrate that the likely impact of the potential selection biases identified in A or B above was minimal?	WN	Sensitivity analyses were performed for reverse causation and sample aliquot, but not specifically for volunteer selection or missing Lp(a) measurement. There is no clear evidence of substantial impact on the Lp(a) estimate.
Risk of bias (due to selection of participants into the study) in the estimated effect of exposure on the outcome	Some concerns	Selection into the cohort and into biomarker analysis raises some concern, especially because UK Biobank had a low participation rate. However, there is limited evidence that selection strongly distorted the Lp(a) and prostate cancer association.
What is the predicted direction of bias<br><br>due to selection of participants into the study?	Insufficient information available	The direction of any selection bias is uncertain.

Signalling questions	Response options	Comments
Is the risk of bias (due to selection of participants into the study) sufficiently high, in the context of its likely direction and the magnitude of the estimated exposure effect, to threaten conclusions about whether the exposure has an important effect on the outcome?	No	Selection concerns should be noted but are not judged high enough to overturn the interpretation of the null Lp(a) result.

Y = Yes; PY = Probably yes; PN = Probably no; N = No; SN = Strong no; WN = Weak no; NA = Not applicable; NI = No information

Domain 4: Risk of bias due to post-exposure interventions

Signalling questions	Response options	Comments
4.1 Were there post-exposure interventions that were influenced by prior exposure during the follow-up period?	PN	The Lp(a) measurement was obtained for research. It is unlikely that participants or clinicians used the measured Lp(a) value to guide PSA testing, biopsy, treatment, or other interventions during follow up.
4.2 **If Y/PY to 4.1**: Is it likely that the analysis corrected for the effect of post-exposure interventions that were influenced by prior exposure?	NA	Not applicable because no clear intervention pathway influenced by measured Lp(a) was identified.
Risk of bias (due post-exposure interventions) in the estimated effect of exposure on the outcome	Low risk	No important bias due to interventions after exposure was identified for the Lp(a) and incident prostate cancer result.
What is the predicted direction of bias due to confounding?	Insufficient information available	No specific direction of bias is evident.
Is the risk of bias (due post-exposure interventions) sufficiently high, in the context of its likely direction and the magnitude of the estimated exposure effect, to threaten conclusions about whether the exposure has an important effect on the outcome?	No	This domain is unlikely to threaten the conclusion.

Y = Yes; PY = Probably yes; PN = Probably no; N = No; NA = Not applicable; NI = No information

Domain 5: Risk of bias due to missing data

Signalling questions	Response options	Comments
5.1 Were complete data on exposure status available for all, or nearly all, participants?	PN	Lp(a) results were available for fewer men than the full analytic cohort. The figure for incident prostate cancer reports 4440 cases with Lp(a) data, whereas the full study reported more incident cases overall. Therefore exposure data were not complete for all or nearly all participants.
5.2 Were complete data on the outcome available for all, or nearly all, participants?	PY	Outcome follow up used linked cancer registration and death records. Outcome data were probably complete for nearly all participants, although routine data linkage may miss a small number of events.
5.3 Were complete data on confounding variables available for all, or nearly all, participants?	Y	The main covariates had low missingness, and the authors used unknown categories for variables with missing data. This reduces loss of participants due to missing covariates.
5.4 **If N/PN/NI to 5.1, 5.2 or 5.3**: Is the result based on a complete case analysis?	PY	For Lp(a), participants without biomarker data appear to have been excluded from that exposure specific analysis. Covariate missingness was largely handled through unknown categories rather than imputation.
5.5 **If Y/PY/NI**: Was exclusion from the analysis because of missing data (in exposure, confounders or the outcome) likely to be related to the true value of the outcome?	PN	There is no clear evidence that missing Lp(a) measurement was related to the true prostate cancer outcome. Missingness likely reflected laboratory availability, sample processing, or assay coverage rather than later cancer status.
5.6 **If N/PN to 5.5**: Were all or most predictors of missingness (in exposure, confounders or the outcome) included in the analysis model?	WY	The model included many predictors that might relate to missingness and prostate cancer diagnosis, including age, region, ethnicity, socioeconomic position, BMI, and lifestyle factors. Missingness itself was not explicitly modelled.
5.7 **If N/PN to 5.4**: Was the analysis based on imputing missing values?	NA	Not applicable because the result appears to rely on available Lp(a) measurements rather than imputed exposure values.
5.8 **If Y/PY to 5.7**: Was imputation performed appropriately?	NA	Not applicable.
5.9 **If N/PN to 5.7**: Was an appropriate alternative method used to correct for bias due to missing data?	WN	No specific inverse probability weighting or other correction for missing Lp(a) data was reported. However, there is no clear evidence that the missing biomarker data caused substantial bias.

Signalling questions	Response options	Comments
5.10 **If PN/N/NI to 5.1, 5.2 or 5.3**: Is there evidence that the result was not biased by missing data?	PN	The paper does not provide direct evidence comparing participants with and without Lp(a) data for prostate cancer risk. The likely impact is uncertain but probably limited.
Risk of bias (due to missing data) in the estimated effect of exposure on the outcome	Some concerns	Incomplete Lp(a) data create some concern, but the missingness mechanism is not clearly related to later prostate cancer diagnosis.
What is the predicted direction of bias due to missing data?	Insufficient information available	The direction of missing data bias cannot be determined from the available information.
Is the risk of bias (due to missing data) sufficiently high, in the context of its likely direction and the magnitude of the estimated exposure effect, to threaten conclusions about whether the exposure has an important effect on the outcome?	No	Missing data concerns are not judged high enough to threaten the overall conclusion for the Lp(a) result.

Y = Yes; PY = Probably yes; PN = Probably no; N = No; SY = Strong yes; WY = Weak yes; NA = Not applicable; NI = No information

Domain 6: Risk of bias arising from measurement of the outcome

**Signalling questions**	**Response options**	**Comments**
6.1 Could measurement or ascertainment of the outcome have differed between exposure groups or levels of exposure?	PN	Outcome measurement used linked cancer registration and death records with ICD coding. The process was unlikely to differ by baseline Lp(a) level. Differential PSA testing is better considered under confounding and detection bias rather than outcome measurement itself.
6.2 Were outcome assessors aware of study participants' exposure history?	PN	Registry and administrative outcome assessment was unlikely to be informed by the participant's baseline Lp(a) concentration measured for research.
6.3 **If Y/PY/NI to 6.2**: Could assessment of the outcome have been influenced by knowledge of participants' exposure history?	NA	Not applicable because outcome assessors were probably not aware of Lp(a) exposure history.
Risk of bias (arising from measurement of outcomes) in the estimated effect of exposure on the outcome	Low risk	Prostate cancer diagnosis was measured using routine linked health data with standardized coding and is unlikely to have been assessed differently by Lp(a) level.
What is the predicted direction of bias arising from measurement of outcomes?	Insufficient information available	No important outcome measurement bias is expected.
Is the risk of bias (arising from measurement of outcomes) sufficiently high, in the context of its likely direction and the magnitude of the estimated exposure effect, to threaten conclusions about whether the exposure has an important effect on the outcome?	No	Outcome measurement bias is unlikely to threaten the conclusion.

Y = Yes; PY = Probably yes; PN = Probably no; N = No; SY = Strong yes; WY = Weak yes; NA = Not applicable; NI = No information

Domain 7: Risk of bias in selection of the reported result

Signalling questions	Response options	Comments
7.1 Was the result reported in accordance with an available, pre-determined analysis plan?	NI	The paper references the UK Biobank protocol and states that covariate categories were defined a priori based on previous analyses, but a specific publicly available analysis plan for this Lp(a) and prostate cancer result was not reported.
7.2 **If N/PN/NI to 7.1**: Is the reported effect estimate likely to be selected, based on desirability of the magnitude (or statistical significance) of the estimated effect of exposure on outcome, from multiple *exposure measurements* within the exposure domain?	PN	The study reports results across the full panel of biomarkers rather than only favourable findings. The Lp(a) estimate for prostate cancer incidence was null and was reported in the figure together with other cardiovascular biomarkers.
7.3 Is the reported effect estimate likely to be selected, based on desirability of the magnitude (or statistical significance) of the estimated effect of exposure on outcome, from multiple *outcome measurements* within the outcome domain?	PN	Incident prostate cancer was defined consistently using ICD 10 code C61 or prostate cancer death. No alternative prostate cancer outcome definition appears to have been selected because of result desirability.
7.4 Is the reported effect estimate likely to be selected, based on desirability of the magnitude (or statistical significance) of the estimated effect of exposure on outcome, from multiple *analyses* of the exposure-outcome relationship?	PN	The authors presented fifth based results, continuous per SD estimates, correction for regression dilution bias, and sensitivity analyses. Multiple testing was addressed using false discovery rate control.
7.5 Is the reported effect estimate likely to be selected, based on the basis of desirability of the results (e.g. statistical significance), from different *subgroups*?	PN	The Lp(a) result was part of the planned cardiovascular biomarker group and was not a selected subgroup result.
Risk of bias (due to selection of the reported result) in the estimated effect of exposure on the outcome	Some concerns	The absence of a result specific registered analysis plan leaves some concern, but broad reporting of biomarkers and formal multiple testing correction reduce the likelihood of selective reporting.
What is the predicted direction of bias due to selection of the reported result?	Insufficient information available	If any selective reporting occurred, the direction is not clear. The assessed Lp(a) result was close to the null.

Signalling questions	Response options	Comments
Is the risk of bias (due to selection of the reported result) sufficiently high, in the context of its likely direction and the magnitude of the estimated exposure effect, to threaten conclusions about whether the exposure has an important effect on the outcome?	No	This domain is not judged high enough to threaten the interpretation of the Lp(a) result.

Y = Yes; PY = Probably yes; PN = Probably no; N = No; NA = Not applicable; NI = No information

Overall risk of bias

	Response options	Comments
Overall risk of bias	Some concerns	Overall judgement: Some concerns. The study has strong features for ROBINS E assessment, including prospective design, large sample size, baseline biomarker measurement, linked registry outcomes, extensive adjustment, correction for regression dilution bias, sensitivity analyses, and multiple testing control. Main remaining concerns are residual detection bias from unmeasured PSA testing and health care contact, incomplete Lp(a) data, low UK Biobank participation, and limited detail on the Lp(a) assay in the main article.
What is the predicted direction of bias?	Insufficient information available	Potential biases could operate in different directions. Measurement error would most likely bias towards the null, whereas detection bias through health care contact could bias away from the null if associated with Lp(a).
Is the overall risk of bias sufficiently high, in the context of its likely direction and the magnitude of the estimated exposure effect, to threaten conclusions about whether the exposure has an important effect on the outcome?	No	The overall risk of bias is not judged sufficiently high to invalidate the result. The Lp(a) association with incident prostate cancer was close to the null, and the available data provide little evidence that higher Lp(a) concentration was associated with prostate cancer incidence in this cohort.

This work is licensed under a Creative Commons Attribution-NonCommercial-NoDerivatives 4.0 International License.
