# Supplementary material for: Lipoprotein(a) and Prostate Cancer: A Systematic Review of Observational, Genetic and Mendelian Randomization Evidence
Source: Int J Mol Sci. 2026 Sep 17;27(18):8274. doi: 10.3390/ijms27188274 (PMC13607466; doi:10.3390/ijms27188274)
Supplement: Supplementary file 1 [file ijms-27-08274-s001.zip › Supplementary Material S6.pdf]

**Author and year of publication**	Wang 2019			
**Study identifier**<br>**Reviewer**				
**Biases**	**Issues to consider for judging overall rating of "Risk of bias"**	**Study Methods & Comments**	**Rating of reporting**	**Rating of "Risk of bias"**
Instructions to assess the risk of each potential bias:	These issues will guide your thinking and judgment about the overall risk of bias within each of the 6 domains. Some 'issues' may not be relevant to the specific study or the review research question. These issues are taken together to inform the overall judgment of potential bias for each of the 6 domains.	Provide comments or text exerpts in the white boxes below, as necessary, to facilitate the consensus process that will follow.	Click on each of the blue cells and choose from the drop down menu to rate the adequacy of reporting as yes, partial, no or unsure.	Click on the green cells; choose from the drop-down menu to rate potential risk of bias for each of the 6 domains as High, Moderate, or Low considering all relevant issues
**1. Study Participation**	**Goal: To judge the risk of selection bias (likelihood that relationship between *PF* and *outcome* is different for participants and eligible non-participants).**			
Source of target population	The source population or population of interest is adequately described for key characteristics (LIST).	The population was Chinese Han men with primary diagnosed, pathologically confirmed sporadic prostate cancer treated at the Department of Urology, Affiliated Hospital of Qingdao University. The broader referral population and catchment area were not described in detail.	Partial	
Method used to identify population	The sampling frame and recruitment are adequately described, including methods to identify the sample sufficient to limit potential bias (number and type used, e.g., referral patterns in health care)	Patients were said to be consecutive, which supports reduced selection bias. However, the paper did not report the number screened, the number eligible, referral patterns, or reasons for non inclusion beyond listed criteria.	Partial	
Recruitment period	Period of recruitment is adequately described	Recruitment period was clearly reported as January 2011 to October 2018.	Yes	
Place of recruitment	Place of recruitment (setting and geographic location) are adequately described	Recruitment took place at a single hospital department in Qingdao, China. The setting was adequately described, although the single centre design may limit representativeness.	Yes	
Inclusion and exclusion criteria	Inclusion and exclusion criteria are adequately described (e.g., including explicit diagnostic criteria or "zero time" description).	Inclusion required primary diagnosed, pathologically confirmed sporadic prostate cancer. Exclusions were other malignancy history, severe liver or renal insufficiency, and incomplete clinicopathological information.	Yes	
Adequate study participation	There is adequate participation in the study by eligible individuals	The study included 376 patients, but no response rate or denominator for all eligible patients was reported. The proportion of high risk disease was large, with 322 high risk cases and 54 low to intermediate risk cases.	Partial	
Baseline characteristics	The baseline study sample (i.e., individuals entering the study) is adequately described for key characteristics (LIST).	Age, body mass index, hypertension, diabetes, coronary artery disease, lipid lowering drug use, lipid variables, surgery type, PSA group, stage, nodal status, metastasis and ISUP grade were reported.	Yes	
**Summary Study participation**	**The study sample represents the population of interest on key characteristics, sufficient to limit potential bias of the observed relationship between PF and outcome.**			Moderate
**2. Study Attrition**	**Goal: To judge the risk of attrition bias (likelihood that relationship between *PF* and *outcome* are different for completing and non-completing participants).**			
Proportion of baseline sample available for analysis	Response rate (i.e., proportion of study sample completing the study and providing outcome data) is adequate.	This was not a longitudinal follow up study. The analysis appears to use all 376 eligible patients with available clinicopathological information and Lp(a) results.	Partial	
Attempts to collect information on participants who dropped out	Attempts to collect information on participants who dropped out of the study are described.	Not applicable to the main analysis because no follow up period was used for the assessed association. The paper did not describe any follow up.	Partial	
Reasons and potential impact of subjects lost to follow-up	Reasons for loss to follow-up are provided.	Loss to follow up was not relevant for the analysed cross sectional clinical association. However, patients with incomplete clinicopathological information were excluded and the number of such patients was not reported.	Partial	
Outcome and prognostic factor information on those lost to follow-up	Participants lost to follow-up are adequately described for key characteristics (LIST).	No participants lost to follow up were described.	Partial	
	There are no important differences between key characteristics (LIST) and outcomes in participants who completed the study and those who did not.	The study did not report the characteristics of patients excluded because of incomplete clinicopathological information.	Partial	
**Study Attrition Summary**	**Loss to follow-up (from baseline sample to study population analyzed) is not associated with key characteristics (i.e., the study data adequately represent the sample) sufficient to limit potential bias to the observed relationship between PF and outcome.**			Moderate

**3. Prognostic Factor Measurement**	**Goal: To judge the risk of measurement bias related to how PF was measured (differential measurement of PF related to the level of outcome).**			
Definition of the PF	A clear definition or description of 'PF' is provided (e.g., including dose, level, duration of exposure, and clear specification of the method of measurement).	The prognostic factor was serum Lp(a). It was analysed across quartiles: less than 104 mg/L, 104 to 216 mg/L, 216 to 381 mg/L, and greater than 381 mg/L. The main result compared quartile 4 with quartile 1	Yes	
Valid and Reliable Measurement of PF	Method of PF measurement is adequately valid and reliable to limit misclassification bias (e.g., may include relevant outside sources of information on measurement properties, also characteristics, such as blind measurement and limited reliance on recall).	Venous blood was collected after overnight fasting before surgery, androgen deprivation, or other therapies. Lp(a) was measured using a turbidimetric immunoassay with a validated standard and reported coefficient of variation below 10%. It was not stated whether the assay was insensitive to apo(a) isoform size or whether laboratory staff were blinded to clinical risk group.	Partial	
	Continuous variables are reported or appropriate cut-points (i.e., not data-dependent) are used.	Lp(a) was reported as medians and quartiles. The quartile thresholds were derived from this study sample, so the cut points were data driven rather than prespecified clinical thresholds.	Partial	
Method and Setting of PF Measurement	The method and setting of measurement of PF is the same for all study participants.	The same blood sampling protocol and laboratory method were reported for all participants.	Yes	
Proportion of data on PF available for analysis	Adequate proportion of the study sample has complete data for PF variable.	The paper reports 376 eligible patients with Lp(a) values distributed across four quartiles. Missing Lp(a) data were not described.	Partial	
Method used for missing data	Appropriate methods of imputation are used for missing 'PF' data.	No imputation was described. Patients with incomplete clinicopathological information were excluded, and missing Lp(a) data were not discussed.	No	
**PF Measurement Summary**	***PF* is adequately measured in study participants to sufficiently limit potential bias.**			Moderate
**4. Outcome Measurement**	**Goal: To judge the risk of bias related to the measurement of outcome (differential measurement of outcome related to the baseline level of PF).**			
Definition of the Outcome	A clear definition of outcome is provided, including duration of follow-up and level and extent of the outcome construct.	The outcome was high risk prostate cancer. It was defined by PSA greater than 20 ng/mL, ISUP grade 4 or higher, or clinical stage T2c or higher. Low to intermediate risk was defined as PSA 20 ng/ mL or lower, ISUP grade 3 or lower, and clinical stage T2b or lower.	Yes	
Valid and Reliable Measurement of Outcome	The method of outcome measurement used is adequately valid and reliable to limit misclassification bias (e.g., may include relevant outside sources of information on measurement properties, also characteristics, such as blind measurement and confirmation of outcome with valid and reliable test).	Pathological data came from TRUS guided biopsy or radical prostatectomy specimens processed using standard procedures. Stage was assessed using AJCC TNM 2002 and grade using ISUP 2014. Risk stratification was conducted by two surgeons blinded to Lp(a) levels.	Yes	
Method and Setting of Outcome Measurement	The method and setting of outcome measurement is the same for all study participants.	The setting was the same hospital department. Some pathological information came from biopsy and some from prostatectomy specimens, which may differ in precision, but all specimens were processed using standard procedures.	Partial	
**Outcome Measurement Summary**	***Outcome of interest* is adequately measured in study participants to sufficiently limit potential bias.**			Low
**5. Study Confounding**	**Goal: To judge the risk of bias due to confounding (i.e. the effect of PF is distorted by another factor that is related to PF and outcome).**			
Important Confounders Measured	All important confounders, including treatments (key variables in conceptual model: LIST), are measured.	The model adjusted for age, body mass index, hypertension, diabetes, coronary artery disease, and lipid lowering drugs. Important factors for this review, including smoking, alcohol, socioeconomic status, family history, PSA testing intensity, diet, physical activity, inflammation markers, renal function severity beyond exclusion criteria, liver function severity beyond exclusion criteria, and ancestry variation beyond restriction to Chinese Han men, were not fully addressed.	Partial	
Definition of the confounding factor	Clear definitions of the important confounders measured are provided (e.g., including dose, level, and duration of exposures).	Age and body mass index were measured. Hypertension, diabetes, coronary artery disease, and lipid lowering drug use were obtained from electronic records and medical charts. Detailed	Partial	

		definitions, timing, and severity of comorbidities were not provided.		
Valid and Reliable Measurement of Confounders	Measurement of all important confounders is adequately valid and reliable (e.g., may include relevant outside sources of information on measurement properties, also characteristics, such as blind measurement and limited reliance on recall).	Confounder information came from electronic records and medical charts, which is acceptable for basic clinical variables, but reliability for comorbidities and medication history was not described.	Partial	
Method and Setting of Confounding Measurement	The method and setting of confounding measurement are the same for all study participants.	Confounders appear to have been obtained from the same records system for all participants.	Partial	
Method used for missing data	Appropriate methods are used if imputation is used for missing confounder data.	No imputation or missing data method was reported. Patients with incomplete clinicopathological information were excluded.	No	
Appropriate Accounting for Confounding	Important potential confounders are accounted for in the study design (e.g., matching for key variables, stratification, or initial assembly of comparable groups).	The design restricted participants to Chinese Han men with primary sporadic prostate cancer and excluded severe liver or renal insufficiency and other malignancies. There was no matching or stratification for key metabolic or detection related factors.	Partial	
	Important potential confounders are accounted for in the analysis (i.e., appropriate adjustment).	Adjustment included several clinical factors, but the analysis did not address several plausible confounders and detection factors. The timing of Lp(a) measurement at diagnosis also leaves uncertainty about whether aggressive disease influenced Lp(a) rather than the reverse.	Partial	
**Study Confounding Summary**	**Important potential confounders are appropriately accounted for, limiting potential bias with respect to the relationship between *PF* and *outcome*.**			High

**6. Statistical Analysis and Reporting**	**Goal: To judge the risk of bias related to the statistical analysis and presentation of results.**			
Presentation of analytical strategy	There is sufficient presentation of data to assess the adequacy of the analysis.	The paper described descriptive testing, quartile grouping, univariate logistic regression, and multivariable logistic regression. Tables reported the main odds ratios, confidence intervals, and p values.	Yes	
Model development strategy	The strategy for model building (i.e., inclusion of variables in the statistical model) is appropriate and is based on a conceptual framework or model.	The model strategy was partly derived from the dataset. The authors stated that Lp(a) was entered into multivariable analysis after being significant in univariate analysis, with adjustment for selected clinical factors. A prespecified conceptual model was not presented.	Partial	
	The selected statistical model is adequate for the design of the study.	Logistic regression was suitable for the binary high risk prostate cancer classification. However, the low to intermediate risk group was small with 54 patients, and confidence intervals were wide.	Partial	
Reporting of results	There is no selective reporting of results.	Results were reported for multiple lipid variables and several clinicopathological comparisons. There was no protocol or statistical analysis plan, and no adjustment for multiple testing was described, so selective reporting cannot be ruled out.	Partial	
**Statistical Analysis and Presentation Summary**	**The statistical analysis is appropriate for the design of the study, limiting potential for presentation of invalid or spurious results.**			Moderate
