# Supplementary material for: Lipoprotein(a) and Prostate Cancer: A Systematic Review of Observational, Genetic and Mendelian Randomization Evidence
Source: Int J Mol Sci. 2026 Sep 17;27(18):8274. doi: 10.3390/ijms27188274 (PMC13607466; doi:10.3390/ijms27188274)
Supplement: Supplementary file 1 [file ijms-27-08274-s001.zip › Supplementary Material S7.pdf]

Quality of genetic association studies (Q-Genie)

Please indicate your selection for each question as follows:

☐ 1	☐ 2	☒ 3	☐ 4	☐ 5	☐ 6	☐ 7
Poor		Good		Very Good		Excellent

1. Rationale for study

Please rate the study on the adequacy of the presented hypothesis and rationale.

When rating the study, please consider the following:

- *Was a scientific rationale for chosen genes presented to avoid selective reporting of positive results?*
  If this is a GWAS design, where a hypothesis-free approach is taken, a rationale for selecting this design should be presented.

☐ 1	☐ 2	☐ 3	☐ 4	☐ 5	☒ 6	☐ 7
Poor		Good		Very Good		Excellent

2. Selection and definition of outcome of interest. The outcome can be cases/disease status or a quantitative trait.

Please rate the study on the classification of the outcome (e.g. disease status or quantitative trait).

When rating the study, please consider the following:

- *Were the cases appropriately defined?*
  Outcome definitions will vary from independent adjudication or reliable laboratory measures (strong) to self-report (moderate) to no-description (poor)
- *Were participants appropriately sampled?*
  Participants should be sampled in a way to avoid selection bias as appropriate to the study objectives (e.g. such as selecting the most sick cases if the objective is not to enrich cases). Included participants should reflect the entire population of interest.
- *Were the case/outcome assessors blinded to the genotype status?*
- *If applicable, was follow-up length appropriate for outcome to occur and was the attrition rate acceptable?*

☐ 1	☐ 2	☐ 3	☐ 4	☐ 5	☒ 6	☐ 7
Poor		Good		Very Good		Excellent

3. Selection and comparability of comparison groups (if applicable)

Please rate the study on appropriateness of comparison groups (e.g. control groups).

When rating the study, please consider the following:

- *Were the controls appropriately defined?*
- *Were the controls sampled in a way to minimize selection bias?*
- *Was a detailed description of selection procedure (i.e. eligibility criteria, sources and methods of ascertainment, methods of matching if applicable) outlined or referenced?*
- *Were the assessors of control status blinded to the genotype status?*

Please note: In multi-ethnic studies, allele frequencies and disease risks may differ. Consequently, confounding may occur if these sub-populations are unevenly distributed across exposure groups (or between cases and controls); therefore, details of the sub-populations (e.g. ethnicity) should be reported.

☐ 1 ☐ 2 ☐ 3 ☐ 4 ☒ 5 ☐ 6 ☐ 7

Poor Good Very Good Excellent

4. Technical classification of the exposure

Please rate the study on the technical classification of the genetic variant.

When rating the study, please consider the following:

- *Was the source (e.g. buffy coat) and method of storage for the DNA sample appropriate?*
- *Were the methods of DNA ascertainment similar for comparison groups (if applicable)?*
- *Was the genotyping platform and allele-calling algorithm appropriate?*
- *Were the genotyping error & call rates appropriate? Call rates below 95% indicate poor genotyping quality.*
- *Were the genotype call rates and SNP missingness similar between the comparison groups?*
- *Was agreement with the Hardy Weinberg equilibrium tested in controls?*
- *If applicable, did the authors check for samples with outlying heterozygocity to assess quality of genotyping?*

Please note: if genotypes are imputed, authors should describe methods and rationale for imputing

☐ 1 ☐ 2 ☐ 3 ☐ 4 ☒ 5 ☐ 6 ☐ 7

Poor Good Very Good Excellent

5. Non-technical classification of the exposure

Please rate the study on the non-technical classification of the genetic variant.

When rating the study, please consider the following:

- *Did a blinded assessor conduct the genotyping?*
- *Was genotyping conducted in all the participants from the study simultaneously or in smaller batches? If so, were methods across batches same?*

- *If applicable, were samples randomized prior to genotyping (e.g. not all controls on one plate and cases on another)?*

☐ 1 ☐ 2 ☐ 3 ☒ 4 ☐ 5 ☐ 6 ☐ 7

Poor Good Very Good Excellent

6. Other sources of bias

Please rate the study on the disclosure and discussion of sources of bias.

In addition to selection and classification bias previously discussed, many other potential sources of bias exist (e.g. time-lag bias, attrition bias, et cetera). Please consider whether all sources of bias were disclosed and their effect on the results discussed.

☐ 1 ☐ 2 ☐ 3 ☐ 4 ☒ 5 ☐ 6 ☐ 7

Poor Good Very Good Excellent

7. Sample size and power

Please rate whether the study was adequately powered.

- *Was the sample size appropriate?*
- *Was an a priori power analysis conducted?*

☐ 1 ☐ 2 ☐ 3 ☐ 4 ☒ 5 ☐ 6 ☐ 7

Poor Good Very Good Excellent

8. A priori planning of analyses

Please rate the study on the planned analyses.

- *Was the analysis plan appropriate and sufficiently described?*
- *Was selective and/or inappropriate reporting avoided (i.e. all results from tests conducted were reported)? Authors should identify where additional results can be found if not included in the primary paper (e.g. supplementary tables).*
- *Were the tested subgroups, interactions, and sensitivity analyses described and reported?*
- *Was the statistical software used identified?*

☐ 1 ☐ 2 ☐ 3 ☐ 4 ☒ 5 ☐ 6 ☐ 7

Poor Good Very Good Excellent

9. Statistical methods and control for confounding

Please rate the study on statistical methods.

- *Were important confounders appropriately controlled?*

- *Were missing data for samples and genetic variant was appropriately handled? >10% missing genotype data is often unacceptable.*
- *Were the results adjusted for multiple testing to avoid false positive results? Please note this is particularly important in analyses of large datasets.*

Please note: For multiethnic studies or those with sub-populations, statistical methods, such as principle components analysis, should control for presence of resultant confounding.

☐ 1	☐ 2	☐ 3	☐ 4	☐ 5	☒	☐ 7
Poor		Good		Very Good		Excellent

10. Testing of assumptions and inferences for genetic analyses

Please rate the study on the description and test of all assumptions and inferences.

- *Were all assumptions concerning the genetic analysis tested?*
  Specifically,
  - *i) Haplotypes may be inferred as a result of lack of availability of family data. Numerous methods exist for inferring haplotypes; authors should specifically report how this inference was made.*
  - *ii) In non-family based studies, some individuals may be distantly related or part of a consanguineous group, which may lead to inaccurate results and should be tested with appropriate measures.*
  - *iii) Reported sex and ethnicity should also be checked prior to conducting analyses.*

☐ 1	☐ 2	☐ 3	☐ 4	☒	☐ 6	☐ 7
Poor		Good		Very Good		Excellent

11. Appropriateness of inferences drawn from results

Please rate the study on whether conclusions drawn by the authors were supported by the results and appropriate methods.

☐ 1	☐ 2	☐ 3	☐ 4	☐ 5	☒	☐ 7
Poor		Good		Very Good		Excellent

Scoring

Please add the total score from each question.

For studies with control groups: Scores ≤35 indicate poor quality studies, >35 and ≤45 indicate studies of moderate quality, and >45 indicate good quality studies.

For studies without control groups: Scores ≤32 indicate poor quality studies, >32 and ≤40 indicate studies of moderate quality, and >40 indicate good quality studies.
