# Supplementary material for: Lipoprotein(a) and Prostate Cancer: A Systematic Review of Observational, Genetic and Mendelian Randomization Evidence
Source: Int J Mol Sci. 2026 Sep 17;27(18):8274. doi: 10.3390/ijms27188274 (PMC13607466; doi:10.3390/ijms27188274)
Supplement: Supplementary file 1 [file ijms-27-08274-s001.zip › Supplementary Material S8.pdf]

Risk of bias assessment (RoB)

RoB for Mendellian Randomization (MR) studies

Author	Weak instrument bias	Genetic confounding bias	‘Other’ confounding bias	Additional direct effects between IV and outcome (exclusion restriction assumption)	Bias due to selection of participants
An et al.	Some concerns	Some concerns	Low	Low	Low
Chen et al.	Low	Some concerns	Low	Low	Low
Fang et al.	Low	Some concerns	Low	Low	Low
Ioannidou et al.	Low	Low	Low	Some concerns	Low
Li et al.	Low	Low	Low	Low	Some concerns
Lin et al.	Low	Some concerns	Low	Some concerns	Some concerns

Adopted from (1). We used following criteria in Risk of Bias (RoB) in MR studies according to Mamluk et al. See supplementary Material.

1. Mamluk L, Jones T, Ijaz S, Edwards HB, Savović J, Leach V, et al. Evidence of detrimental effects of prenatal alcohol exposure on offspring birthweight and neurodevelopment from a systematic review of quasi-experimental studies. Int J Epidemiol. 2021 Jan 23;49(6):1972–95. doi:10.1093/IJE/DYZ272 PubMed PMID: 31993631.
