# Supplementary material for: Lipoprotein(a) and Prostate Cancer: A Systematic Review of Observational, Genetic and Mendelian Randomization Evidence
Source: Int J Mol Sci. 2026 Sep 17;27(18):8274. doi: 10.3390/ijms27188274 (PMC13607466; doi:10.3390/ijms27188274)
Supplement: Supplementary file 1 [file ijms-27-08274-s001.zip › Supplementary Table S1.pdf]

Supplementary Table S1. Summary of observational evidence on circulating Lp(a) and prostate cancer outcomes.

Study	Population	Factors analyzed	Lp(a) measurment	Correlation with PCa	No correlation with PCa/ insigificant correlation
Katzke	Heidelberg cohort	PCa incidence	Serum based Lp(a) measurements	1. Q1 vs Q4 serum Lp(a) concentrations (adjusted HR 1,43 1.02-2.03), with Q4 serum cut-off at 42.25.	1. Quartile-median trend (p= 0.085)<br>2. Continuous model (per doubling of Lp(a)) (HR 1.06, 95% CI 1.00–1.13; p = 0.051)
Perez-tornago	UK-Biobank	PCa incidence and mortality	Serum based Lp(a) measurements	NA	1. PCa incidence (HR 1.02 CI 0.99-1.05)<br>2. PCa death - HR, lowest, medium and highest third of Lp(a) concentration no significant association;<br>3. p-trend = 0.68)<br>4. Continuous model (per 1 SD higher Lp(a), corrected for regression dilution bias), HR 1.03 (0.91–1.16)
Marrer	PRIME-study cohort	PCa incidence	Serum based Lp(a) measurements	NA	Q4 vs Q1 Lp(a) concentrations: HR 0.84, 95% CI 0.49–1.45), after excluding cases diagnosed within first 2 years (Q4 vs Q1: HR 0.87, 95% CI 0.50–1.51), p = 0.12, 0.10 consequently
