# Supplementary material for: Lipoprotein(a) and Prostate Cancer: A Systematic Review of Observational, Genetic and Mendelian Randomization Evidence
Source: Int J Mol Sci. 2026 Sep 17;27(18):8274. doi: 10.3390/ijms27188274 (PMC13607466; doi:10.3390/ijms27188274)
Supplement: Supplementary file 1 [file ijms-27-08274-s001.zip › Supplementary Table S2.pdf]

Supplementary Table S2. Summary of Mendelian randomization evidence on genetically predicted Lp(a) and prostate cancer risk.

Study	Database	Lp(a) – PCa correlation
Ioannidou et a.	UK Biobank - GWAS	Main analysis:<br>-OR $_{IVW}$ = 1.066; 95% CI = [0.909,1.249]; p = 0.431<br>-OR $_{weighted\ median\ approach}$ = 1.091(95% CI = [1.028,1.157]; Pp= 0.004<br>Sensitivity analysis 1:<br>-OR $_{IVW}$ = 1.076; 95% CI = [1.016,1.114]; p = 0.012);<br>-OR $_{weighted\ median}$ = 1.066; 95% CI = [1.012,1.123]; p = 0.016<br>Sensitivity analysis 2:<br>-OR $_{IVW}$ = 1.037; 95% CI = [1.009,1.066]; p = 0.010,<br>-OR $_{weighted\ median}$ = 1.044; 95% CI = [1.026,1.061]; p = 6.58 × 10−7<br>Sensitivity analysis 3:<br>-OR $_{IVW}$ = 1.228; 95% CI = [0.960,1.570]; p = 0.104<br>-OR $_{weighted\ median}$ = 1.439; 95% CI = [1.280,1.619]; p = 1.80 × 10^{-9};<br>Multivariable analysis:<br>-OR = 1.068; 95% CI = [1.005,1.134]; p = 0.034
Fang et al.	UK-Biobank - GWAS	-OR $_{IVW}$ = 1.06, 95% CI = [0.95, 1.20], p = 0.305<br>-OR $_{weighted\ median}$= 1.07, 95% CI = [1.004, 1.13], p = 0.036<br>- rs73596816 variant OR = 1.07, 95% CI = [1.00, 1.14], p = 0.056<br>-multivariable MR OR =1.05, 95% CI = [1.01, 1.08], p = 0.013
Chen et al.	GWAS	- OR = 1.061, 95% CI = [1.018,1.105], p = 0.005
Lin et al.	Uk biobank, Finngen	PCa forward MR:<br>-OR$_{IVW}$ = 1.120348291, p = 0.038093372
Li et al.	Uk biobank, Finngen	Main two sample MR analysis:<br>-OR$_{RE\ IVW}$ = 1.12, 95% CI = [1.01-1.25]; p=0.038<br>MR egger:<br>-OR = 1.22, 95% CI = [1.04-1.43]; p=0.035<br>Weighted median MR:<br>-OR = 1.11, 95% CI = [1.00, 1.23]; p=0.053<br>Robust adjusted profile score<br>-OR = 1.15, 95% CI=[1.00,1.32]; p=0.04<br>Debiased inverse-variance weighted method<br>-OR = 1.12, 95% CI= [1.01-1.24]; p=0.032<br>Constrained maximum likelihood<br>-OR = 1.12, 95% CI = [1.03-1.22]; p=0.011<br>Bayesian Weighted Mendelian Randomization<br>-OR = 1.13, 95% CI = [1.02, 1.26]; p=0.02
An et al.	Uk biobank, Finngen	Inverse variance weighted:<br>-OR 1.12 (95% CI 1.00,1.24); p=0.040<br>Bayesian Weighted Mendelian Randomization:<br>-OR 1.09 (95% CI 0.97, 1.22); p=0.136<br>MR-Egger:<br>-OR 1.08 (95% CI 0.92, 1.28); p=0.344<br>Weighted median:<br>OR 1.16 (95% CI 1.06, 1.26); p= 0.001<br>Simple mode:

		-OR 1.19 (95% CI 1.00, 1.41); p=0.067<br>Weighted mode:<br>-OR 1.19 (95% CI 1.09,1.29); p= 0.001
